# Protein Structure-Based Organic Chemistry-Driven Ligand Design from Ultralarge Chemical Spaces

**DOI:** 10.1021/acscentsci.3c01521

**Published:** 2024-02-13

**Authors:** François Sindt, Anthony Seyller, Merveille Eguida, Didier Rognan

**Affiliations:** Laboratoire d’innovation thérapeutique, UMR7200 CNRS-Université de Strasbourg, Illkirch 67400, France

## Abstract

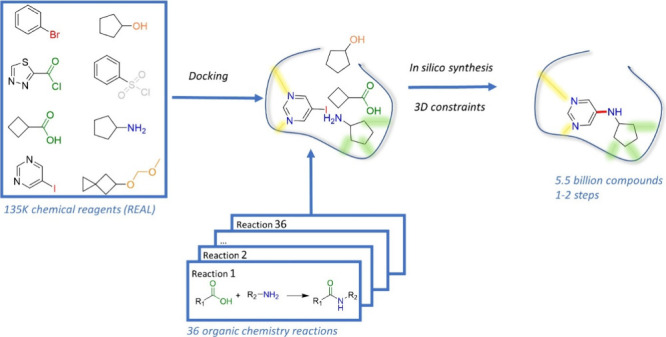

Ultralarge chemical spaces describing several billion compounds are revolutionizing hit identification in early drug discovery. Because of their size, such chemical spaces cannot be fully enumerated and require ad-hoc computational tools to navigate them and pick potentially interesting hits. We here propose a structure-based approach to ultralarge chemical space screening in which commercial chemical reagents are first docked to the target of interest and then directly connected according to organic chemistry and topological rules, to enumerate drug-like compounds under three-dimensional constraints of the target. When applied to bespoke chemical spaces of different sizes and chemical complexity targeting two receptors of pharmaceutical interest (estrogen β receptor, dopamine D3 receptor), the computational method was able to quickly enumerate hits that were either known ligands (or very close analogs) of targeted receptors as well as chemically novel candidates that could be experimentally confirmed by *in vitro* binding assays. The proposed approach is generic, can be applied to any docking algorithm, and requires few computational resources to prioritize easily synthesizable hits from billion-sized chemical spaces.

## Abstract 2

A fast, structure-based method is described to screen billions of easily synthesizable molecules.

## Introduction

Identifying the first hit compounds able to target a macromolecule of interest is often achieved by screening experimentally or computationally a library of drug-like compounds,^1^ thereby enabling a hit to lead follow-up using classical medicinal chemistry strategies.^2^ Until recently, the commercially available chemical space describing drug-like compounds amenable to screening has been restricted to 10–15 million compounds with a yearly growth of ca. half a million compounds.^3^ On-demand compound libraries^4,5^ have completely changed this situation by proposing billions of compounds not yet available but easily synthesizable in a few steps and reproducible parallel synthesis. Early approaches to virtually screen subsets of ultralarge chemical spaces led to spectacular successes,^6−9^ notably unexpected high hit rates, very high potencies, and fine selectivity.^10,11^ Today, ca. 70 billion compounds are accessible on-demand with fast delivery (6–8 weeks) and high-purity grade (>95%).^12^ Due to their huge size, compounds describing these ultralarge chemical spaces cannot be fully enumerated and require dedicated computational tools for registration, storage, and navigation.^13^ Usually, large chemical spaces are described in a combinatorial manner from the building blocks and organic chemistry reactions required to synthesize them.^4^ If ligand-based approaches are now available to efficiently query these large chemical spaces,^14−16^ structure-based approaches including macromolecular target information (e.g., topology of a binding site) still need to be developed to exhaustively mine multibillion chemical spaces. Several computational methods have indeed been described for such a task,^17−23^ albeit with moderate to severe restrictions. One the one hand, exhaustive docking of 1.4 billion compounds^18^ has been successfully described with the help of costly dedicated platforms,^18,24^ but will soon reach its limits with next-to-come trillion-sized chemical spaces^25^ since full atomistic docking just scales linearly with the number of compounds to be screened. A workaround consists of the proper selection of seed fragments/scaffolds to screen a representative subset of the entire space. The seed fragment may originate from the early docking of fragment-based representative synthons,^23^ X-ray diffraction screening data,^22^ or medicinal chemistry knowledge.^20^ Once a seed fragment has been identified, scaffold-focused two-dimensional (2D) libraries, exploring the corresponding chemical space via a set of organic chemistry reactions,^26^ can be enumerated, converted in three-dimensional (3D) atomic coordinates and physically docked to propose novel hits. This approach has been applied with success to a few targets^20,22,27,23^ but still requires hardware settings enabling docking a significant subset (a few million) of the entire chemical space. Last, fast machine learning approaches may be first trained on a set of representative ligand-annotated docking poses to simply predict docking scores^17,19,21,28,29^ and next be applied to predict docking scores for the remaining space. Even if only a small fraction of the full space (1–5%) has to be docked at the atomic level, this strategy cannot be further applied to trillion-sized chemical spaces since it would require gathering the first billion of docking scores on a single target. Moreover, this approach has led to very mitigated results with respect to hit rate and hit potencies^30^ and deserves further experimental validations.

Herein, we present a simple and fast computational approach (SpaceDock) avoiding the above-cited drawbacks. It first requires docking commercially available chemical reagents to the target of interest in order to couple them according to standard organic chemistry reactions to propose multibillion compound libraries in one or two synthetic steps. When applied to two targets of pharmaceutical interest, the method was able to quickly retrieve hits that are chemically identical (or very close) to existing ligands but also to propose chemically novel and potent ligands.

## Results

Since the SpaceDock method heavily relies on the possibility to accurately dock chemical reagents, we first investigated the best docking protocols for the latter task by setting up a dedicated benchmarking study. We then describe how chemical reagents are annotated by reactive groups and organic chemistry reactions to define a chemical space of 5.5 billion synthesizable compounds. Last, we present two concrete applications of the SpaceDock workflow to two receptors of pharmaceutical interest.

### Setting up the Conditions for Accurate Docking of Chemical Reagents

**Figure 1:**
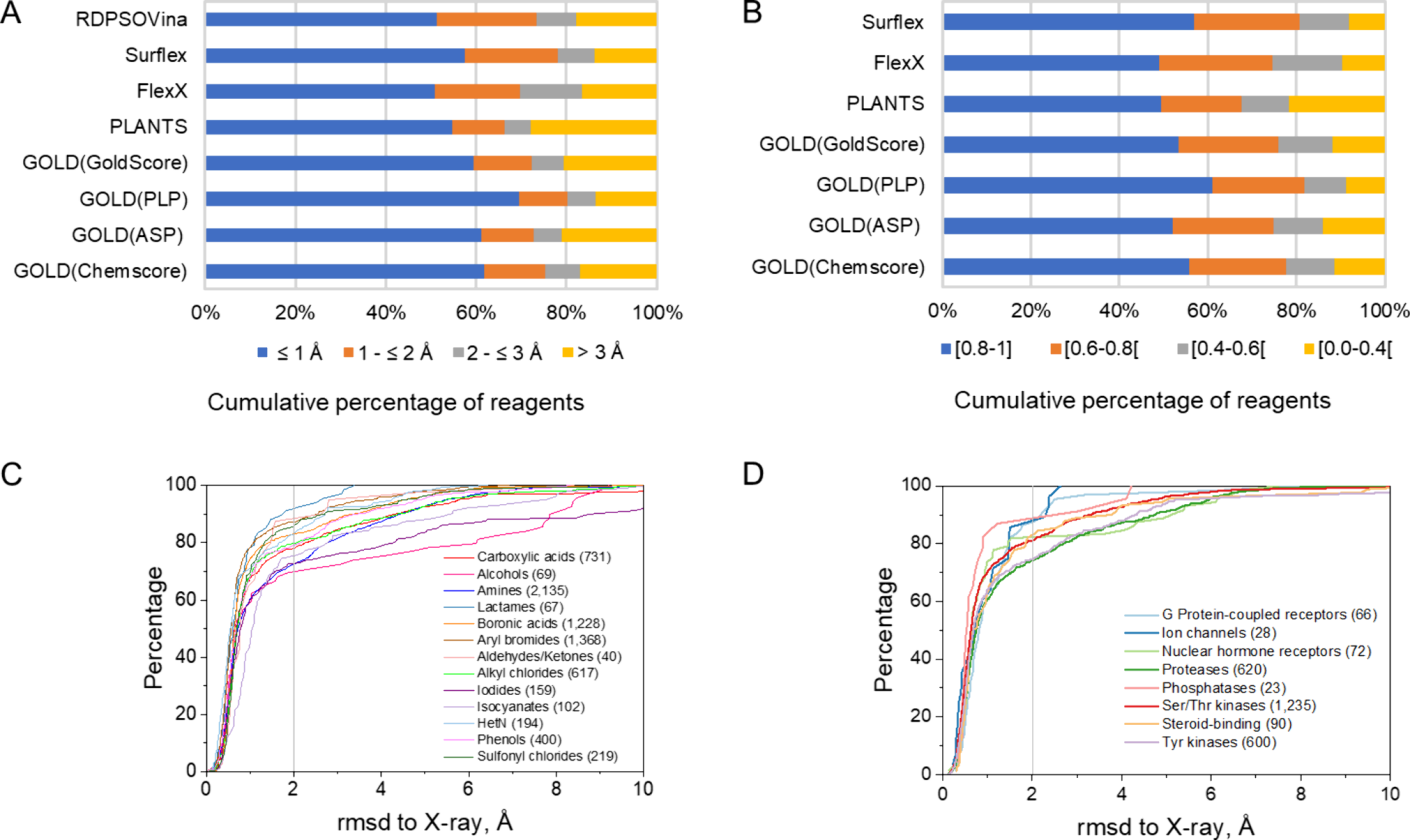
Accuracy of state-of-the-art docking tools to dock 5,845 sc-PDB reagents in their cognate targets. (A) Root-mean square deviation (rmsd) of the best pose (lowest rmsd, heavy atoms only) to the surrogate X-ray structure, (B) similarity of protein-reagent interaction fingerprints between the best pose (highest interaction fingerprint similarity) and surrogate X-ray structures, measured by a Tanimoto coefficient. Fingerprints could not be measured for RDPSOVina poses in pdbqt format, (C) cumulative rmsd of the best pose (GOLD-PLP docking) for each of the 13 chemical functions. Numbers in brackets indicate the absolute number of each chemical function, (D) cumulative rmsd of the best pose (GOLD-PLP docking), according to protein class. Numbers in brackets indicate the absolute number of samples from each protein family.

To evaluate the feasibility of the SpaceDock approach, we first needed to set up an archive of reference 3D structures for protein-bound chemical reagents. Since experimental data for such a data set are missing, we fragmented in 3D space drug-like ligands from known protein–ligand X-ray structures (sc-PDB data set)^31^ using a set of 12 common organic chemistry reactions, then added the 3D atomic coordinates of the missing reactive moieties (e.g., boronic acid, halide; Figure S1), and last created on-the-fly “surrogate X-ray poses” for the corresponding reagents expected to yield the parent ligands with the above-described reactions. The final archive of 5,845 reagents was selected after appropriate filtering (Table S1) and exhibited 13 chemical functions with a prevalence of reactive groups (e.g., amines, aryl halides, boronic acids) reflecting the frequent usage of simple organic chemistry reactions in drug discovery.^32^ With a set of reference reagents in hand, we next verified whether state-of-the-art docking algorithms were able to reproduce the surrogate X-ray poses. Five algorithms relying on different principles (FlexX:^33^ incremental construction, GOLD:^34^ genetic algorithm, PLANTS:^35^ ant colony optimization, RDPSOVina:^36^ random drift particle swarm optimization, Surflex:^37^ surface-based molecular similarity) were used for that purpose. Since the SpaceDock strategy just needs a single pair of complementary reagents to be properly docked to reconstitute a full ligand, the docking performance was measured by computing the root-mean square deviation (rmsd) of the pose found to be the closest (best pose) to that of the surrogate X-ray structure (Figure 1). All docking tools exhibit an excellent docking performance, with 70–80% of chemical reagents being docked within 2 Å rmsd accuracy (Figure 1A). Up to 70% of very high-quality poses (rmsd < 1 Å) could be generated by the apparently best docking/scoring scheme (GOLD docking, PLP scoring; Figure 1A). The observed docking accuracy is therefore independent of the chosen docking algorithm and remains in agreement with docking benchmarks on low molecular weight fragments.^38,39^ Since the rmsd is a global measure that does not take into account whether key protein-reagent interactions are verified or not, we additionally computed the similarity of protein-reagent interaction fingerprints (IFPs)^40^ between docked and surrogate X-ray poses. Again, an excellent performance could be noticed using this orthogonal quality descriptor, with 75–85% of chemical reagents for which the IFP similarity to the X-ray pose is deemed acceptable (Tc-IFP > 0.60;^40^Figure 1B). To ascertain that all chemical functions are equally suitable for docking, the same analysis was repeated for each of the 13 chemical groups (Figure 1C) present in our library, focusing on the best docking strategy (GOLD docking and PLP scoring). Reassuringly, the docking performance appears to be relatively independent of the chemical function of the reagent (Figure 1C) as well as of the target protein family (Figure 1D).

### Defining a Readily Accessible Ultralarge Chemical Space from Simple Organic Chemistry Reactions

**Figure 2:**
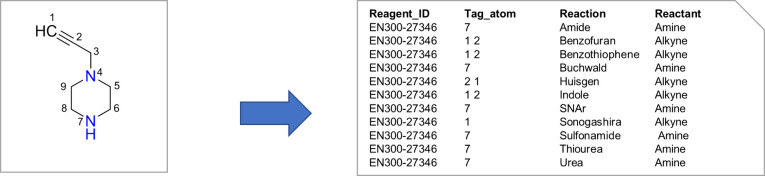
Annotation of chemical reagents by reaction type, reactant role, and reactive atoms.

Starting from the pioneering work of Hartenfeller et al.,^26^ we selected 36 robust, stereo- and regioselective organic chemistry reactions to define a chemical space of 5.5 billion compounds readily accessible in one or two synthesis steps (Table S2, Figure S2). Contrary to previous similar approaches,^26,41,42^ chemical reagents were here carefully chosen from specific SMARTS strings in a list of 145,705 commercial chemical reagents contributing to Enamine’s REAL space^43^ of 36 billion compounds. Moreover, possible side reactions affecting synthesis yields were minored by selecting reagents that are monofunctional for a particular chemical function (e.g., monocarboxylic acid) and lacking additional chemical functions (e.g., nucleophilic groups for an electrophilic reactant) that would decrease the reaction yield (Table S2). Altogether, 134,331 commercial reactants could be unambiguously annotated by reaction type, reactant role, and reactive atoms, yielding a total of 713,155 atomic tags (Figure 2). Conversion in 3D atomic coordinates provided a total of 176,824 ready-to-dock unique reagents, ionized at pH 7.4, including stereoisomers for reactants bearing up to two undefined chiral centers.

### Retrospective Chemical Space Docking of 97 Million Compounds for Human Estrogen Receptor Beta Agonists

**Figure 3:**
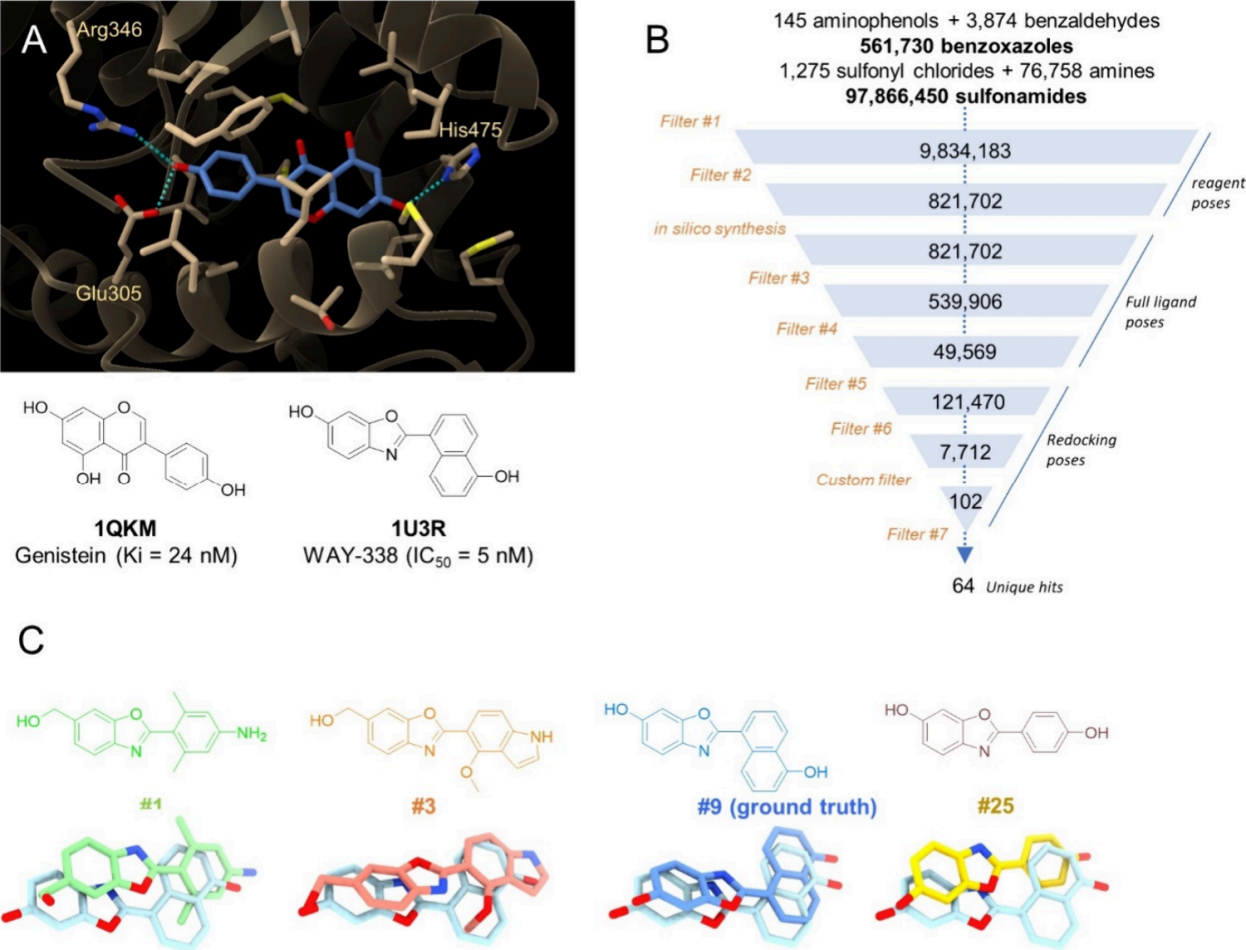
Space docking of benzoxazole and sulfonamide chemical spaces to human estrogen receptor beta (ERβ). (A) X-ray structure of human ERβ (tan ribbons, PDB entry 1QKM) in complex with the agonist genistein (blue sticks). The genistein binding site is delimited by ERβ residues displayed as tan sticks with main receptor–ligand hydrogen bonds indicated by cyan broken lines. The known benzoxazole agonist (WAY-338) is taken as the ground truth ligand to recover. (B) SpaceDock flowchart affording 64 potential ERβ agonists according to a series of filters (Table 1). The custom filter (H-bond either Glu305 or Arg346, and to His475) is target-specific. (C) Structures and rank (#) of 4 representative benzoxazoles. The proposed binding poses are overlaid to the X-ray pose of the ground truth ligand (WAY-338, cyan), the protein being masked for the sake of clarity.

For a first proof-of-concept, we selected as a target the activated form of the human estrogen receptor beta (ERβ) for the following two reasons: (i) the ligand-binding cavity is nicely druggable with a good hydrophobicity/hydrophilicity balance, (ii) the receptor has been cocrystallized with many high-affinity low molecular-weight agonists, notably compounds sharing a 2-aryl-benzoxazole scaffold^44^ whose one-step synthesis from 2-aminophenols and benzaldehydes is one of the 36 reactions that we have encoded. To avoid a possible chemotype bias, we selected an X-ray receptor structure cocrystallized with genistein (PDB 1QKM), a nonbenzoxazole high-affinity agonist used from here on as the “reference ligand” (Figure 3A) and asked whether we could recover a “ground truth” benzoxazole agonist (WAY-338, Figure 3A) or any close analog, by first docking the necessary reactants (2-aminophenols, benzaldehydes) and then enabling the benzoxazole ring formation within the protein binding site. To this end, 145 commercial 2-aminophenols and 3,874 benzaldehydes were generated in 3D and docked into the 1QKM structure, in order to explore a combinatorial space of 561,730 possible benzoxazoles. Since the later space is small, we additionally considered a much larger space of 97 million sulfonamide decoys synthesizable from 1,275 sulfonyl chlorides and 76,758 amines, thereby strongly minoring the benzoxazole space (0.57%) in the full chemical space to scan. After docking all reagents necessary to mine both chemical spaces according to the previously found best protocol (GOLD docking, PLP scoring), a series of filters of increasing complexity (Table 1) was iteratively passed to a decreasing number of possible solutions, first starting with pairs of potentially reacting reagent poses, then with successfully enumerated ligand poses, and last with quality checked redocking poses.

**Table 1:** Incremental Series of Filters Applied to Prioritize SpaceDock Hits

filter	type	criteria	applies to	software used
1	Geometry	Distances, angles, clashes	Pair of reactant poses	this work
2	Interaction	Interaction fingerprint similarity to reference	Pair of reactant poses	IChem^45^
3	Energy, geometry	Rmsd of refined pose to nonrefined pose	Fully enumerated ligand	Szybki^46^
				Surflex-Dock^37^
4	Interaction, structure	Interaction fingerprint similarity (IFP) to reference	Fully enumerated ligand	IChem^45^
		Number of stereocenters, number of rotatable bonds		Filter^46^
		Drug-likeness		
5	Redocking	Rmsd to energy-minimized SpaceDock pose	Docking poses	GOLD^34^
		IFP similarity to energy-minimized SpaceDock pose		Surflex-Dock^37^
				IChem^45^
6	Quality check	Number of strained torsions, local and global strain energy	Docking poses	Torsion_analyzer^47^ Freeform^46^
		Number of unsatisfied H-bond donors and acceptors, number of unsatisfied ionic bonds		this work
7	Final selection	Duplicates removal	Docking poses	This work
		Absolute binding free energy (HYDEscore)		Hydescorer^48^

The SpaceDock flowchart is displayed Figure 3. In a first step, pure chemical and topological filters (Figures S3 and S4) are passed to all docking poses of possible reactant pairs to quickly remove impossible reactions (filter #1). To stay on a safe side, we only considered pairs of bound reactants exhibiting a total interaction fingerprint (IFP) similarity^40^ to the genistein X-ray pose above an acceptable threshold^40^ (IFP ≥ 0.60 considering all nonbonded interactions, IFP ≥ 0.50 considering polar interactions only; filter #2). The 821,702 remaining pairs of reactants were then converted, in the protein 3D space, into the corresponding benzoxazoles and sulfonamides, respectively, and the fully enumerated ligands were quickly minimized in the protein binding site. Only 539,906 poses deviated by less than 1.0 Å rmsd from the nonrefined poses after energy refinement (filter #3). The remaining minimized poses were filtered again according to IFP similarity to the genistein X-ray pose (IFP ≥ 0.60 considering all nonbonded interactions, IFP ≥ 0.60 considering polar interactions only; filter #4). Compounds with more than 2 stereocenters and 8 rotatable bonds were removed at this stage, leaving 49,569 poses for further processing. To ensure that the selected SpaceDock poses might be recovered by classical docking, all remaining hits were redocked to the ERβ structure, as previously done for the reagents. Only 121,470 poses close to the corresponding energy-minimized SpaceDock poses (rmsd ≤ 2.0 Å; IFP ≥ 0.60 considering all nonbonded interactions, IFP ≥ 0.60 considering polar interactions only) were retained (filter #5). A quality check of remaining poses (filter #6) was next applied to remove unlikely solutions (≥1 strained torsion, local strain energy >4 kcal/mol, global strain energy >8 kcal/mol, no unsatisfied ionic bond, >2 unsatisfied H-bond donors, >4 unsatisfied h-bond acceptors).^49,20^ The number of plausible solutions (7,712) being still important, a custom filter was finally applied to keep only poses anchored at both sides of the binding pocket (H-bond either Glu305 or Arg346, and to His475), as seen for all potent ERβ agonists (recall genistein X-ray pose, Figure 3A). The final hit list comprises 102 poses from 64 unique ligands (filter #7), including 54 benzoxazoles and 10 sulfonamides (Figure 3B, Table S3) ranked by decreasing full IFP similarity to the reference ligand, then by decreasing polar IFP similarity, and last by increasing absolute binding free energy predicted by the HYDE scoring function.^48^

Despite being in the minority in the initial space (0.57%), it is reassuring that the ground truth chemotype was considerably enriched (84%) in the final hit list. Inspecting the structures and binding poses of the hits, we observed that SpaceDock was indeed able to recover, among the top-ranked hits, the ground truth ligand (rank #9), a known ERβ agonist ChEMBL187673^50^ (IC_50_ = 50 nM, rank #25) and 52 other 2-arylbenzoxazoles, with almost perfect binding modes (rmsd = 1.15 Å for the ground-truth ligand, Figure 3C). About half of the hits (30 out 64; all from the benzoxazole space) were considered chemically similar (according to a Tanimoto coefficient measured on circular ECFP4 fingeprints) to existing ERβ ligands (Figure S5), evidencing that SpaceDock can propose both known ligands (or very close analogs thereof) and new chemical entities. However, only a lower number of compounds (17, out of which 10 share the sulfonamide space) strictly intersected the Enamine REAL space (Figure S5). This observation does not preclude for their synthesizability but just illustrates that these hits, despite the commercial availability of their starting building blocks, cannot be obtained within the scope of 167 parallel synthesis protocols defining REAL space.

From this preliminary proof-of-concept, it appears that the herein presented method is able to perform a complex organic chemistry reaction (ring cyclization) from suitably posed and chemically compatible chemical reagents, under the 3D constraints of the target’s structure, to generate and prioritize fully enumerated ligands for meaningful reasons. We therefore decided to apply SpaceDock to the prospective screening of a much larger chemical space.

### Prospective Chemical Space Docking of 670 Million Compounds for Human Dopamine D3 Receptor Antagonists

**Figure 4:**
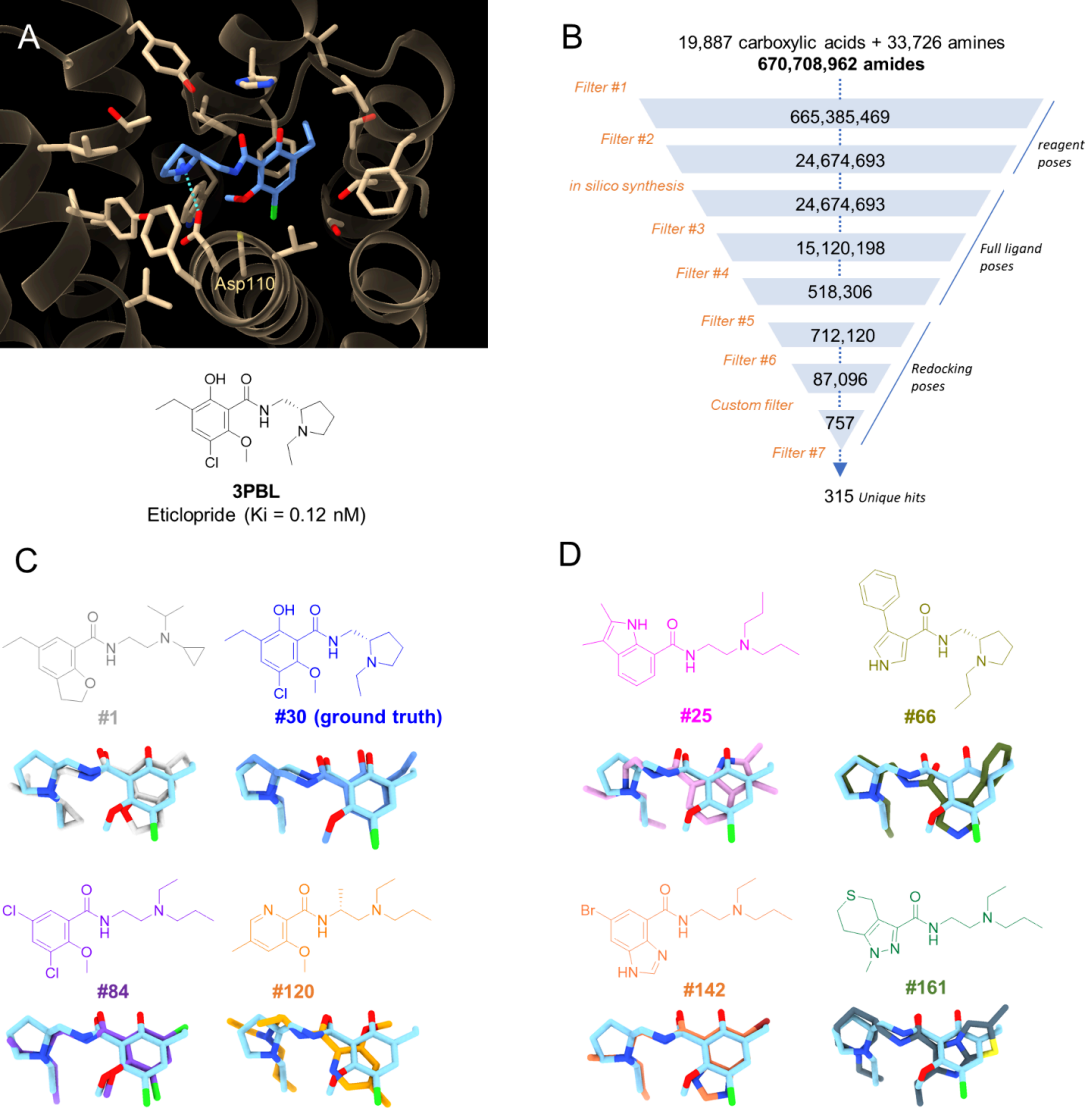
Space docking of an amide in chemical space to the human dopamine D3 receptor (DRD3). (A) X-ray structure of human DRD3 (tan ribbons, PDB entry 3PBL) in complex with the antagonist eticlopride (blue sticks). The eticlopride binding site is delimited by DRD3 residues displayed as tan sticks with the main receptor–ligand ionic bond indicated by cyan broken lines. Eticlopride is taken as both the reference and the ground truth ligand to recover. (B) SpaceDock flowchart affording 315 potential DRD3 antagonists according to a series of filters (Table 1). The custom filter (IFP similarity to an eticlopride X-ray pose) is target-specific. (C) Structures and rank of 4 representative orthomethoxybenzamides. The proposed binding poses are overlaid to the X-ray pose of the ground truth ligand (eticlopride, cyan), the protein being masked for the sake of clarity. (D) Structure and binding poses of other hits aligned to the X-ray pose of eticlopride.

We next applied the method to a much larger chemical space of 670 million carboxamides targeting the human dopamine D3 receptor (DRD3). Since the only available high-resolution DRD3 receptor structure (PDB 3PBL) has been obtained in complex with the antagonist eticlopride (Figure 4A),^51^ the latter orthomethoxybenzamide (OMB) ligand was used as both reference and ground-truth ligand to recover. Commercially available carboxylic acids and primary/secondary amines (Table S2) were first filtered to remove reagents that, upon amide bond formation, would lead to nondrug-like ligands (Table S4), thereby keeping 19,887 acids and 33,726 amines (in 3D coordinates) to explore a chemical space of 670 million carboxamides (Figure 4B). The resulting 53,613 chemical reagents were then docked to the eticlopride-free DRD3 structure using GOLD docking and PLP scoring, as previously described. Since 20 poses were saved for each reactant, a total of 268 billion (19,887*20*33,726*20) possible reactions were passed to the SpaceDock flowchart (Figure 4B), removing first impossible amide bond formation according to geometrical criteria (Figure S6) while keeping only amine poses exhibiting the crucial ionic bond to the key Asp110 residue^51^ (filter #1, Figure 4B), then retaining a pair of reactant poses for which the IFP similarity to the reference ligand is higher than 0.60 for all interactions and 0.50 for polar interactions only (filter #2).^40^ A total of 24,674,693 reactions were conducted *in silico* to generate the corresponding carboxamides inside the receptor pocket, which were later energy-minimized. Keeping only minimized poses that did not deviate much from the initial pose (rmsd < 1.0 Å) afforded 15,120,198 plausible solutions (filter #3, Figure 4B). At this stage, hits bearing a cis-amide bond or more than 2 chiral centers or more than 9 rotatable bonds were removed to keep only drug-like compounds. The resulting number of hits being still very high, we pruned the hit list by keeping only minimized poses with a high full IFP similarity to the reference ligand (IFP similarity > 0.60) while exhibiting a perfect IFP similarity to eticlopride (IFP = 1) with respect to polar interactions (H-bond and ionic bond to Asp110). This filter (filter #4, Figure 4B) yielded 518,306 SpaceDock poses (corresponding to 500,041 unique compounds) that had to be confirmed by full atomistic docking (GOLD docking, PLP scoring, 20 poses saved) of the corresponding ligands and comparison with the minimized SpaceDock poses. Only docking poses verifying the following three criteria (rmsd ≤ 2.0 Å and IFP_full ≥ 0.60 and IFP_polar = 1) were retained, leaving 712,120 good docking poses (filter #5, Figure 4B) for sanity check (no strained torsion, local strain energy ≤4 kcal/mol, global strain energy ≤8 kcal/mol, no unsatisfied ionic bond, ≤ 2 unsatisfied H-bond donors, ≤ 4 unsatisfied H-bond acceptors, filter #6, Figure 4B). The number of remaining poses being still important (97,096), a custom filter (not implemented by default, Table 1) was added to remove poses for compounds with no aromatic ring (always present in known DRD3 antagonists),^52^ exhibiting a predicted absolute binding free energy (HYDEscore) lower than 30 kJ/mol and further restricting the deviation to the original SpaceDock poses (rmsd ≤ 1.0 Å and IFP_full ≥ 0.75). A reasonable number of 757 docking poses from 315 unique ligands (filter #7, Figure 4B) defined the final hit list. Compounds were ranked by decreasing full IFP similarity to the reference ligand, then by decreasing polar IFP similarity, and last by increasing the HYDE binding free energy (Table S5).

**Figure 5:**
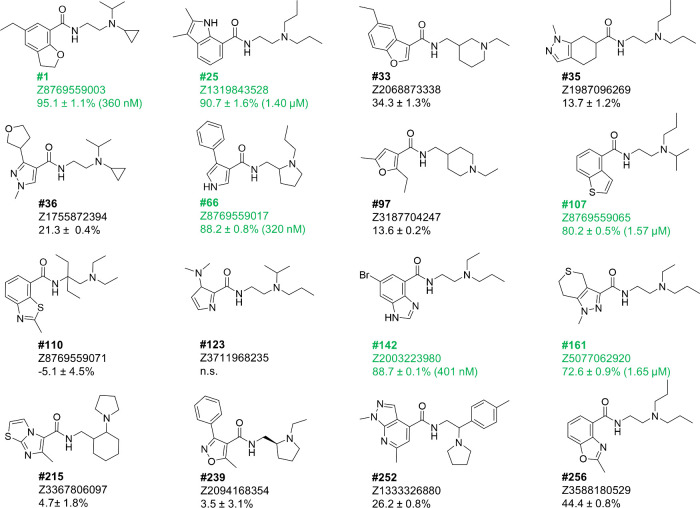
Structure and binding to human DRD3 of 15 SpaceDock hits from amide space. Hits are labeled according to their SpaceDock rank, Enamine’s catalog identifiers, and purchased as racemates, unless specified. Binding affinities to human DRD3 are expressed as the percentage of inhibition of [^3^H]-methylspiperone binding to human recombinant DRD3 expressed in CHO cells (Eurofins Discovery assay #48) at a single concentration of a 10 μM competitor (mean of two independent experiments). The inhibition constant (*K*_i_) was determined from dose–response curves for six strong binders (in green). Compound #123 could not be synthesized (n.s.).

As for the first attempt on ERβ ligands, we first checked whether the ground-truth ligand and its corresponding OMB scaffold were present in the list. Indeed, 15 OMBs including eticlopride (rank 30) were part of the list with binding poses very similar to that observed for the reference ligand (rmsd of eticlopride = 0.73 Å, Figure 4C). Interestingly, 300 additional hits not sharing the OBM scaffold were prioritized with poses and protein–ligand interaction patterns quite close to those seen for eticlopride (Figure 4D). Most ligands were scaffold hops for which the orthomethoxybenzamide has been replaced by a bicyclic heteroaryl-amide, connected by 2–3 carbon atoms to a basic amine. By comparison to the ERβ hit list, the DRD3 hits deviate more from known ChEMBL ligands (24% considered as chemically similar) but are more easily obtainable in REAL space (53% being directly purchasable and an additional 38% being very close to REAL space compounds; Figure S7). Sixteen chemically diverse and representative hits were directly purchased at Enamine, out of which 15 could be synthesized in 6 weeks (5 mg quantity, >90% purity) and further tested for binding to human DRD3 (Figure 5).

Out of the tested 15 compounds, ten exhibited detectable binding (>20% inhibition) to the DRD3 receptor at the single concentration of 10 μM (Figure 5). The six strongest binders (#1, #25, #66, #107, #142, and #161) were selected for dose-curve responses for inhibition constants (*K*_i_) determination (Figure 5, Figure S8). Three of them (#1, #66, #142) exhibited *K*_i_ values in the 300–400 nM range, the three others at 1.4–1.6 μM. The remarkable hit rates (66% at 10 μM, 20% at 500 nM) are in line with previous observations from docking ultralarge libraries^10,11^ and suggests that SpaceDock competes rather well with much more demanding full atomistic docking when screening large chemical spaces.

Interestingly, novel heteroamatic-carboxamide scaffolds were disclosed for 4 of the strong binders (#66, #107, #142, and #161) that could not be found in any of 6,714 dopamine DRD2/DRD3 ligands from ChEMBL (Table 2). SpaceDock proposals should still be considered as primary hits. As such, their potency is lower than that of the closest dopamine D2/D3 antagonists from ChEMBL, albeit with a higher ligand efficiency.

**Table 2:**
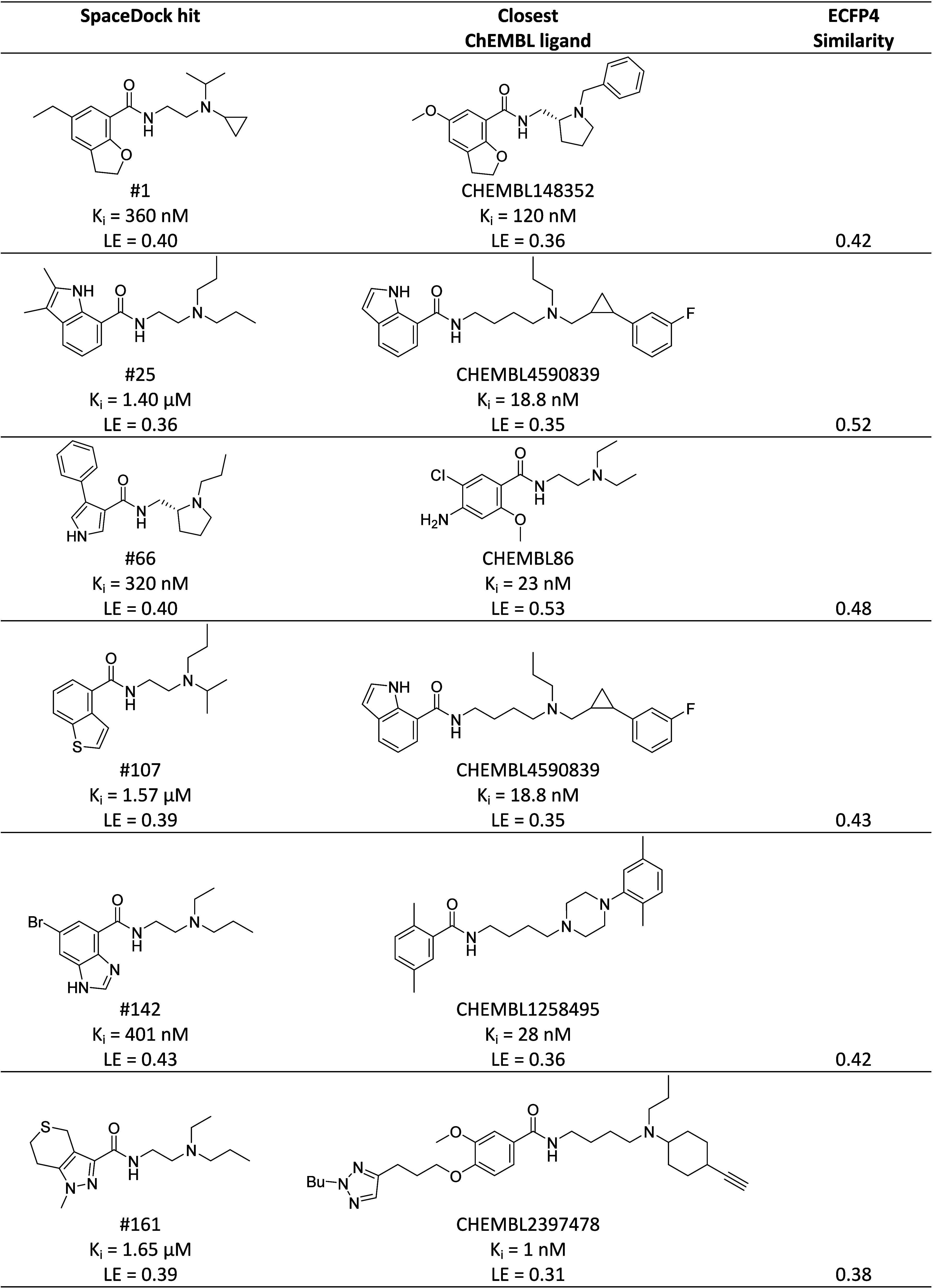
Chemical Similarity between SpaceDock Hits and Their Closest ChEMBL Ligands^a^

a Inhibition constants for human DRD3 (*K*_i_)^50^ and ligand efficiency (LE)^53^ are given for comparison. Similarity is expressed by the Tanimoto coefficient computed on ECFP4 circular fingerprints.

## Conclusion

We herein describe a novel computational method (SpaceDock) to exhaustively browse ultralarge chemical spaces under specific constraints of a target protein and known binders. When applied to two nicely druggable targets (estrogen receptor β, dopamine D3 receptor) and chemical spaces of up to 670 million compounds, it enabled the fast recovery of known ligands/scaffolds (in both cases) and the identification of novel and potent new chemical entities (dopamine D3 receptor).

SpaceDock departs from existing methods^20,22,23^ by two major differences: (i) fully unmodified chemical reagents and not synthons (scaffolds with chemistry-informed exit vectors) are used as primary sources of hits, (ii) most promising ligands are directly obtained within the protein binding site, by 3D *in silico* synthesis according to geometrical and chemical cross-compatibility of previously posed reagents pairs.

Indeed, direct docking of chemical reagents has, to the best of our knowledge, never been reported. Interestingly, our preliminary benchmark demonstrates that docking chemical reagents is as accurate as docking low-molecular weight fragments^39^ with ca. 75% of chemicals properly posed with respect to their corresponding substructures in full PDB ligands. Noteworthy, the docking accuracy is independent of the docking tool used, of the reactive moiety of the reactants and of the target protein family; therefore, opening the method to any druggable target and set of commercial building blocks. To enable an easy synthetic access to most SpaceDock hits, the method relies on chemical reagents contributing to Enamine’s REAL space and generate hits in the binding site 3D space using a set of 36 robust two-component organic chemistry reactions. Given the 70% average docking accuracy of reactants, we therefore expect the likelihood of properly coupling two chemically compatible reactants into a fully enumerated and suitably posed ligand at ca. 50%. Of course, the chemical moieties engaged in the organic chemistry reaction are considered during the initial docking step. In case a function is wrongly posed and/or strongly interacting with the target, it might not be available for further linking if topological and chemical compatibility with the second posed reactant is no more verified. Docking the starting chemical reagents is clearly the most time-consuming step of the entire flowchart (ca. 15 s/reagent), meaning that SpaceDock scales with the number of reactants and not the number of products defining the chemical space to be screened. To optimize the speed of the further processing, a series of filters of increasing complexity is applied, step by step, to a decreasing number of plausible solutions. Just checking the relative position of compatible reactants to be paired by fast distance/angle measures permits removal of 99.8% of possible solutions. Although not mandatory, we applied IFP similarity to a reference pose to remove topologically valid ligands that do not fulfill expected interactions with key residues. This filter permits reducing the number of full ligand poses to the third most time-consuming but necessary energy-minimization step (ca. 1 s/recombined pose) and remove local strains around the newly created bonds. We assume that a SpaceDock proposal is all the more interesting if it does not vary (in terms of rmsd and IFP similarity) upon energy minimization within the protein binding site and if it can be recovered by full atomistic docking of the corresponding ligand. Although not necessary, we recommend this redocking step to ensure that SpaceDock and any state-of-the-art docking tool (we here used GOLD, but other tools may be used as well) agree on the final poses to be sent to the very important quality check. A particular importance is given to local and global strain energies (≤4 and 8 kcal/mol, respectively), as well as to the number of unsatisfied ionic bonds (none) and of unsatisfied hydrogen-bond donors/acceptors (≤2 and 4, respectively). In the DRD3 test case, omitting this step drastically enriched the final hit list in false positives, which could not be confirmed experimentally (data not shown). The herein proposed chemical space docking approach could yield, at least for the present case of a G protein-coupled receptor, to experimentally validated hits with a high hit rate and nanomolar potencies that agree with tendencies already noticed upon full atomistic docking of ultralarge library virtual screens.^10,11^

SpaceDock remains a relatively light computational procedure, since browsing a chemical space of 100 million compounds can be achieved within 2 days on a 16-core Intel^(R)^ Xeon^(R)^ Silver 4210 processor. Mining the entire 5.5 billion chemical space has been made possible for the fourth international CACHE challenge^54^ with still limited resources (1 week on 400 cores). Preliminary attempts to scan even larger chemical spaces (e.g., by adding three-component reactions) suggest that the method can be easily applied up to a trillion compounds.

## Methods

### Setting up a Library of Chemical Reagents from Fragmented Protein-Bound Ligands

37,922 ligands from the sc-PDB database of druggable protein–ligand 3D structures^55,31^ were fragmented using a set of 12 RECAP^56^-inspired retrosynthetic rules to yield 97,024 chemical reagents (Figure S1) with standard topologies (bond length, angle bending, torsion angles) retrieved from the TRIPOS force-field.^57^ The resulting building blocks were then filtered using the following rules: (i) IChem v.5.2.8^45^ detection of at least four noncovalent interactions (one of which being a ionic bond or an hydrogen-bond) with the original sc-PDB target protein, (ii) a total number of heavy atoms between 3 and 23, (iii) a total number of rotatable bonds inferior or equal to 6, (iv) a heteroatom to carbon ratio between 0.05 and 4.5, (v) no more than two fused cycles, (vi) a number of aromatic rings inferior to 3. The final library comprised 5,845 reagents (mol2 file format) derived from 4,656 unique sc-PDB ligands. Although the building blocks have not been explicitly crystallized with their target, the corresponding poses will be further annotated as the “surrogate X-ray” pose.

### Docking sc-PDB Building Reagents to Their Cognate Targets

The above-described reagents were docked to the sc-PDB target originally bound to the ligand they were derived of, after randomizing their initial orientation and dihedral angles with the Surflex^37^*ran_archive* routine, using 5 state-of-the-art docking tools (FlexX v.5.2.0,^33^ GOLD v.2022,^34^ PLANTS v1.2,^35^ RDPSOVina v.2.0,^36^ Surflex v.4.5.4.3^37^) with almost standard parameters (Tables S6–S8). Since the boron atom is not parametrized in some docking tools, it was replaced by either a dummy atom (FlexX, GOLD, PLANTS, and Surflex) or a carbon (RDPSOvina) while keeping the trigonal planar geometry of the boronic acid unchanged. Up to 20 poses were preferentially saved in mol2 file format whenever possible (GOLD, PLANTS, Surflex), in sd file format (FlexX), or in pdbqt file format (RDPSOVina). For each docking pose, the root-mean-square deviation (rmsd) of heavy atoms to the corresponding surrogate X-ray pose was computed thanks to the Surflex *rms* routine when comparing mol2 files, or the ADFRsuite-1.0^58^*obrms* routine when comparing files of different formats (mol2 vs pdbqt, mol2 vs sd). In addition, we measured the similarity of protein–ligand interactions between docked and X-ray poses with the IFP module of the IChem v.5.2.8 package.^45^

### Preparation of Bespoke Chemical Spaces Encoded by 36 Robust Organic Chemistry Reactions

The global stock of commercially available building blocks (250,355 compounds, sd file format, date: 2022-12-28) was downloaded from Enamine’s Web site^59^ and filtered by catalog identification number to retain 145,707 reagents contributing to the REAL space.^43^ Building blocks were then filtered to remove unsuitable entries as previously described.^41^ For each of 36 different one- or two-step organic chemistry reactions (Table S2), the corresponding reactants were retrieved using SMARTS strings^41^ queries in PipelinePilot v.22.1.0.2935^60^ (Figure S9). In order to avoid side reactions, building blocks need to be monofunctional for the reactive group of interest and free of any possible poisoning chemical function for the reaction of interest (Table S2). For each retained building block and possible reaction, an annotation triplet is provided: (i) reaction type, reactant role, and reactive atoms. The final annotation table comprises 713,155 annotation triplets for 134,331 REAL building blocks. Selected building blocks were finally ionized at their most likely ionization state at pH 7.4 using PipelinePilot and converted into 3D atomic coordinates with Corina v.3.40,^61^ allowing the generation of up to 4 diastereoisomers by entry, in a single ready-to-dock mol2 file format.

### Docking of Chemical Reagents to Human Estrogen Receptor Beta

The X-ray structure of the human estrogen receptor beta in complex with the agonist genistein^62^ was downloaded from the Protein Data Bank (PDB 1QKM). Hydrogen atoms and simultaneous optimization of protonation states of protein, water, and ligand atoms were performed with Protoss v.4.0.^63^ All water molecules and genistein were removed, keeping only the remaining protein atoms of chain A, which were saved in mol2 file format. The commercial building blocks selected for a possible benzoxazole ring or sulfonamide bond formation (145 aminophenols and 3,874 benzaldehydes; 1,275 sulfonyl chlorides and 76,758 amines) were docked to the ERβ atomic coordinates with GOLD using previously reported parameter settings (Table S7). The cavity was detected from the X-ray atomic coordinates of genistein. Up to 20 poses, scored by the PLP scoring function, were retained for each building block.

### Docking of Chemical Reagents to the Human Dopamine D3 Receptor (DRD3)

The X-ray structure of the human dopamine D3 receptor in complex with the antagonist eticlopride^51^ was downloaded from the Protein Data Bank (PDB 3PBL). Hydrogen atoms and simultaneous optimization of protonation states of protein, water, and ligand atoms was performed with Protoss v.4.0.^63^ The inserted T4-lysozyme sequence (Asn1002-Tyr1161), all water molecules, and eticlopride were removed, keeping only remaining protein atoms of chain A, which were saved in mol2 file format. The commercial building blocks were initially filtered based on their capacity to form a drug-like molecule through an amide bond formation (Table S4) and their inclusion in the pool of reagents utilized in the REAL Space. The reagents selected for a possible amide bond formation (33,726 amines and 19,887 carboxylic acids) were docked to the DRD3 atomic coordinates with GOLD using previously reported parameter settings (Table S7). The cavity was detected from the X-ray atomic coordinates of eticlopride. Up to 20 poses, scored by the PLP scoring function, were retained for each building block. To decrease the number of possible recombinations, only docking poses of amines exhibiting an ionic bond to the key residue Asp110, detected on the fly with IChem, were further retained for amide bond formation.

### Ligand Enumeration by Reagents Coupling

Given two poses of chemically compatible reagents, a ligand is generated within the protein binding site according to their respective location and chemical compatibility. Reagent poses are initially loaded using an in-house mol2 parser and annotated for at least one reaction based on the tag table shown in Figure 2. Atomic coordinates of reactive atoms and their immediate neighbors are extracted and stored for subsequent calculations. This process is repeated for each reaction following a similar workflow. A subsequent set of filters is applied to pairs of reagent poses, including the distance between their center of mass to promptly eliminate distant pairs, the distance between connectable atoms, examination of certain angles of the future formed bond/ring to ensure a suitable geometry, and consideration of clashes (≤4 between nonreacting atoms) to prevent overlapping substituents. If a pair satisfies all of the rules, a bond is created between the connectable atoms. The hybridization of reacting atoms is then updated to reflect the newly created bonds, and exit atoms (to be removed after the reaction) are deleted. The fully enumerated molecule is then saved into a single mol2 file. An optional step is also available at this stage. If a reference ligand exists, the molecule is initially written to a temporary mol2 file to assess its IFP similarity (default values are ≥0.60 for all nonbonded interactions and ≥0.50 for polar interactions) to the reference pose using IChem v.5.2.8. If the similarity threshold is reached, the molecule is transferred to the final mol2 file. Detailed rules of these filters can be found in Figures S3, S4, and S6. The fully enumerated molecule, in the presence of the target protein, is last energy-minimized in Szybki v2.4.0.0,^46^ using standard settings and the MMFF94 force-field.^64^

### Comparisons to Reference Ligands

Interaction fingerprint similarity search between any pose (before and after energy refinement) and a reference X-ray ligand was done using standard parameters of the IFP module implemented in the IChem v.5.2.8 package.^45^ Likewise, root-mean square deviations were computed with the *rms* routine of Surflex-Dock v.4.5.4.3.^37^

### Redocking of SpaceDock Poses

The coupling of two reagent poses, followed by protein constraint refinement (referred to as the “SpaceDock” pose), was redocked into the target protein structure using GOLD. The scoring function employed was PLP, with 20 generated poses, and the same parameter file as described in Table S7. To eliminate structural biases, input ligand structures were converted to SMILES format using the OEChem Toolkit v.3.4.0.1^46^ and further transformed into 3D structures with Corina v.3.40.^61^ Up to four diastereoisomers were generated in a single mol2 file. The resulting full atomistic docking pose, exhibiting a rmsd (computed with Surflex rms) below 2 Å, all nonbonded interactions IFP similarity ≥0.60, and precisely the same polar IFP as the corresponding SpaceDock pose, was considered as confirmation and retained for subsequent investigations. If multiple docking poses satisfy these rules for each SpaceDock pose, then all of them are retained.

### Quality Check of Redocked Poses

The number of torsion strains in every redocking pose was estimated with TorsionAnalyzer v.2.0.0.^47^ Any pose with at least one torsion annotated as “strained” was discarded from further analysis. Local strain (distortion of the specific conformation from the nearest local minima) and global strain (energy required to select the specific conformation from the full conformational ensemble of the corresponding compound in water) energies were then computed with a standard parameter of Freeform v.2.4.0.0.^46^ Any pose with local and global strain energies higher than 4 and 8 kcal/mol, respectively, were discarded.

Last, remaining poses were inspected, in their protein-bound state, for counting the number of unsatisfied ionic bonds, hydrogen-bond donors, and acceptors. First, protein–ligand ionic and hydrogen bonds were registered with IChem. Any charged atom or hydrogen-bond donor/acceptor atom of the ligand (according to IChem definitions)^40^ not present in the above list was annotated as an “unsatisfied” atom. Unsatisfied heavy atoms being both donors and acceptors (e.g., hydroxyl oxygen atom) were counted only once. Ligand atoms participating in intramolecular hydrogen bonds were considered as satisfied. Altogether, ligand poses with more than 2 unsatisfied donors and 4 unsatisfied acceptors were removed from the final hit list.

### Similarity to ChEMBL and REAL Space Ligands

Known ligands of the human estrogen receptor beta (CHEMBL242) and human dopamine D2 (CHEMBL217) and D3 (CHEMBL234) receptors were retrieved from the ChEMBL database (release 33)^50^ as SMILES strings for ligand entries fulfilling the following criteria: *K*_i_ < 1 μM, assay_type = B. Pairwise chemical similarity between SpaceDock hits and ChEMBL ligands was computed with PipelinePilot v.22.1.0.2935^60^ from ECFP4 circular fingerprints and scored by the value of the Tanimoto coefficient.

Maximum common substructure (MCS) similarity of SpaceDock hits (converted from mol2 to SMILES strings, thanks to Open Babel v.3.1.0)^65^ to 36 billion REAL space ligands (version REALSpace_36bn_2023-03.space^12^) was computed with SpaceMACS v.0.9.2,^15^ to save the top 15 REAL space compounds ranked by decreasing MCS-Tanimoto similarity value.

## Data Availability

List of reactants to build benzoxazole, sulfonamide, and amide chemical spaces, docked poses of test reactants (ERβ, DRD3 test cases), annotation table of Enamine REAL reactants, IChem configuration files for IFP filtering. All data and SpaceDock processing scripts are available at https://github.com/litfsindt/LIT-SpaceDock (accessed 01-23-2024). Code availability: Filter v.4.2.1.1, Szbyki v2.5.1.1, OEChem Toolkit v.3.4.0.1; Freeform v.2.5.1.1: OpenEye Scientific, Santa Fe, N.M., USA, https://www.eyesopen.com/ (accessed 01-23-2024) FlexX v.5.2.0, Hyde v.1.5.0, SpaceMACS v.0.9.2, REAL space in fragment space format: BioSolveIT GmbH, Sankt Augustin, Germany, www.biosolveit.de (accessed 01-23-2024) GOLD v.2022: CCDC Software Ltd., Cambridge CB2 1EZ, United Kingdom, www.ccdc.cam.ac.uk (accessed 01-23-2024) Open Babel v.3.1.0, https://github.com/openbabel/openbabel (accessed 01-23-2024) PLANTS v1.2: University of Konstanz, Germany, http://www.tcd.uni-konstanz.de/research/plants.php (accessed 01-23-2024) RDPSOVina v2.0: Jiangnan University, Jiangsu, China, https://github.com/li-jin-xing/RDPSOVina (accessed 01-23-2024) SpaceDock v.1.0.0: https://github.com/litfsindt/LIT-SpaceDock (accessed 01-23-2024) Surflex-Dock v4.5.4.3: BioPharmics LLC, https://www.biopharmics.com (accessed 01-23-2024).

## Supporting Information Available

Set of 12 organic chemistry rules to process specific bonds in sc-PDB ligands and generate building blocks with defined functional groups, cumulative size of the accessible chemical space for 36 organic chemistry reactions, chemical and topological rules to form a benzoxazole ring, chemical and topological rules to form a sulfonamide bond, overlap of Erβ SpaceDock hits to ChEMBL and REAL space, chemical and topological rules to form an amide bond, overlap of DRD3 SpaceDock hits to ChEMBL and REAL space, binding of six SpaceDock hits to the human dopamine D3 receptor, workflow to select reaction-specific reactants from SMARTS strings, rules to filter chemical reagents from fragmented sc-PDB ligands, set of 36 organic chemistry reactions to prepare a combinatorial space of 5.5 billion compounds, SpaceDock hits as potential estrogen receptor beta agonists, rules to filter commercial reagents for drug-likeness of amides to be synthesized, SpaceDock hits as potential dopamine D3 receptor antagonists, parameter settings for PLANTS docking, parameter settings for GOLD docking, parameter settings for RDPSOVina docking, Surflex-Dock and FlexX docking (PDF)
Transparent Peer Review report available (PDF)

## References

[ref1] Bleicher K. H.; Bohm H. J.; Muller K.; Alanine A. I. Hit and Lead Generation: Beyond High-Throughput Screening. Nat. Rev. Drug Discovery 2003, 2, 369–378. 10.1038/nrd1086. 12750740

[ref2] Hughes J. P.; Rees S.; Kalindjian S. B.; Philpott K. L. Principles of Early Drug Discovery. Br. J. Pharmacol. 2011, 162, 1239–1249. 10.1111/j.1476-5381.2010.01127.x. 21091654 PMC3058157

[ref3] Lucas X.; Gruning B. A.; Bleher S.; Gunther S. The Purchasable Chemical Space: A Detailed Picture. J. Chem. Inf. Model. 2015, 55, 915–924. 10.1021/acs.jcim.5b00116. 25894297

[ref4] Grygorenko O. O.; Radchenko D. S.; Dziuba I.; Chuprina A.; Gubina K. E.; Moroz Y. S. Generating Multibillion Chemical Space of Readily Accessible Screening Compounds. iScience 2020, 23, 101681 10.1016/j.isci.2020.101681. 33145486 PMC7593547

[ref5] Tingle B. I.; Tang K. G.; Castanon M.; Gutierrez J. J.; Khurelbaatar M.; Dandarchuluun C.; Moroz Y. S.; Irwin J. J. Zinc-22 - a Free Multi-Billion-Scale Database of Tangible Compounds for Ligand Discovery. J. Chem. Inf. Model. 2023, 63, 1166–1176. 10.1021/acs.jcim.2c01253. 36790087 PMC9976280

[ref6] Lyu J.; Wang S.; Balius T. E.; Singh I.; Levit A.; Moroz Y. S.; O’Meara M. J.; Che T.; Algaa E.; Tolmachova K.; et al. Ultra-Large Library Docking for Discovering New Chemotypes. Nature 2019, 566, 224–229. 10.1038/s41586-019-0917-9. 30728502 PMC6383769

[ref7] Sadybekov A. A.; Brouillette R. L.; Marin E.; Sadybekov A. V.; Luginina A.; Gusach A.; Mishin A.; Besserer-Offroy E.; Longpre J.-M.; Borshchevskiy V.; Cherezov V.; Sarret P.; Katritch V. Structure-Based Virtual Screening of Ultra-Large Library Yields Potent Antagonists for a Lipid GPCR. Biomolecules 2020, 10, 1634 10.3390/biom10121634. 33287369 PMC7761830

[ref8] Stein R. M.; Kang H. J.; McCorvy J. D.; Glatfelter G. C.; Jones A. J.; Che T.; Slocum S.; Huang X. P.; Savych O.; Moroz Y. S.; et al. Virtual Discovery of Melatonin Receptor Ligands to Modulate Circadian Rhythms. Nature 2020, 579, 609–614. 10.1038/s41586-020-2027-0. 32040955 PMC7134359

[ref9] Alon A.; Lyu J.; Braz J. M.; Tummino T. A.; Craik V.; O’Meara M. J.; Webb C. M.; Radchenko D. S.; Moroz Y. S.; Huang X. P.; et al. Structures of the Sigma2 Receptor Enable Docking for Bioactive Ligand Discovery. Nature 2021, 600, 759–764. 10.1038/s41586-021-04175-x. 34880501 PMC8867396

[ref10] Lyu J.; Irwin J. J.; Shoichet B. K. Modeling the Expansion of Virtual Screening Libraries. Nat. Chem. Biol. 2023, 19, 712–718. 10.1038/s41589-022-01234-w. 36646956 PMC10243288

[ref11] Sadybekov A. V.; Katritch V. Computational Approaches Streamlining Drug Discovery. Nature 2023, 616, 673–685. 10.1038/s41586-023-05905-z. 37100941

[ref12] Readily-Accessible on-Demand Chemical Spaces, https://www.biosolveit.de/Infinisee (accessed 11-16-2023).

[ref13] Warr W. A.; Nicklaus M. C.; Nicolaou C. A.; Rarey M. Exploration of Ultralarge Compound Collections for Drug Discovery. J. Chem. Inf. Model. 2022, 62, 2021–2034. 10.1021/acs.jcim.2c00224. 35421301

[ref14] Bellmann L.; Penner P.; Rarey M. Topological Similarity Search in Large Combinatorial Fragment Spaces. J. Chem. Inf. Model. 2021, 61, 238–251. 10.1021/acs.jcim.0c00850. 33084338

[ref15] Schmidt R.; Klein R.; Rarey M. Maximum Common Substructure Searching in Combinatorial Make-on-Demand Compound Spaces. J. Chem. Inf. Model. 2022, 62, 2133–2150. 10.1021/acs.jcim.1c00640. 34478299

[ref16] Meyenburg C.; Dolfus U.; Briem H.; Rarey M. Galileo: Three-Dimensional Searching in Large Combinatorial Fragment Spaces on the Example of Pharmacophores. J. Comput. Aided Mol. Des. 2023, 37, 1–16. 10.1007/s10822-022-00485-y. 36418668 PMC10032335

[ref17] Gentile F.; Agrawal V.; Hsing M.; Ton A. T.; Ban F.; Norinder U.; Gleave M. E.; Cherkasov A. Deep Docking: A Deep Learning Platform for Augmentation of Structure Based Drug Discovery. ACS Cent. Sci. 2020, 6, 939–949. 10.1021/acscentsci.0c00229. 32607441 PMC7318080

[ref18] Gorgulla C.; Boeszoermenyi A.; Wang Z. F.; Fischer P. D.; Coote P. W.; Padmanabha Das K. M.; Malets Y. S.; Radchenko D. S.; Moroz Y. S.; Scott D. A.; et al. An Open-Source Drug Discovery Platform Enables Ultra-Large Virtual Screens. Nature 2020, 580, 663–668. 10.1038/s41586-020-2117-z. 32152607 PMC8352709

[ref19] Berenger F.; Kumar A.; Zhang K. Y. J.; Yamanishi Y. Lean-Docking: Exploiting Ligands’ Predicted Docking Scores to Accelerate Molecular Docking. J. Chem. Inf. Model. 2021, 61, 2341–2352. 10.1021/acs.jcim.0c01452. 33861591

[ref20] Beroza P.; Crawford J. J.; Ganichkin O.; Gendelev L.; Harris S. F.; Klein R.; Miu A.; Steinbacher S.; Klingler F. M.; Lemmen C. Chemical Space Docking Enables Large-Scale Structure-Based Virtual Screening to Discover Rock1 Kinase Inhibitors. Nat. Commun. 2022, 13, 6447 10.1038/s41467-022-33981-8. 36307407 PMC9616902

[ref21] Graff D. E.; Aldeghi M.; Morrone J. A.; Jordan K. E.; Pyzer-Knapp E. O.; Coley C. W. Self-Focusing Virtual Screening with Active Design Space Pruning. J. Chem. Inf. Model. 2022, 62, 3854–3862. 10.1021/acs.jcim.2c00554. 35938299

[ref22] Muller J.; Klein R.; Tarkhanova O.; Gryniukova A.; Borysko P.; Merkl S.; Ruf M.; Neumann A.; Gastreich M.; Moroz Y. S.; et al. Magnet for the Needle in Haystack: ″Crystal Structure First″ Fragment Hits Unlock Active Chemical Matter Using Targeted Exploration of Vast Chemical Spaces. J. Med. Chem. 2022, 65, 15663–15678. 10.1021/acs.jmedchem.2c00813. 36069712

[ref23] Sadybekov A. A.; Sadybekov A. V.; Liu Y.; Iliopoulos-Tsoutsouvas C.; Huang X. P.; Pickett J.; Houser B.; Patel N.; Tran N. K.; Tong F.; et al. Synthon-Based Ligand Discovery in Virtual Libraries of over 11 Billion Compounds. Nature 2022, 601, 452–459. 10.1038/s41586-021-04220-9. 34912117 PMC9763054

[ref24] Gadioli D.; Vitali E.; Ficarelli F.; Latini C.; Manelfi C.; Talarico C.; Silvano C.; Cavazzoni C.; Palermo G.; Beccari A. R., Exscalate: An Extreme-Scale in-Silico Virtual Screening Platform to Evaluate 1 Trillion Compounds in 60 h on 81 Pflops Supercomputers. arXiv:2110.11644v1, 2021.

[ref25] Neumann A.; Marrison L.; Klein R. Relevance of the Trillion-Sized Chemical Space ″Explore″ as a Source for Drug Discovery. ACS Med. Chem. Lett. 2023, 14, 466–472. 10.1021/acsmedchemlett.3c00021. 37077402 PMC10108389

[ref26] Hartenfeller M.; Eberle M.; Meier P.; Nieto-Oberhuber C.; Altmann K. H.; Schneider G.; Jacoby E.; Renner S. A Collection of Robust Organic Synthesis Reactions for in Silico Molecule Design. J. Chem. Inf. Model. 2011, 51, 3093–3098. 10.1021/ci200379p. 22077721

[ref27] Penner P.; Martiny V.; Bellmann L.; Flachsenberg F.; Gastreich M.; Theret I.; Meyer C.; Rarey M. Fastgrow: On-the-Fly Growing and Its Application to DYRK1a. J. Comput. Aided Mol. Des. 2022, 36, 639–651. 10.1007/s10822-022-00469-y. 35989379 PMC9512872

[ref28] Roggia M.; Natale B.; Amendola G.; Di Maro S.; Cosconati S., Streamlining Large Chemical Library Docking with Artificial Intelligence: The Pyrmd2dock Approach. J. Chem. Inf. Model. 2023, 10.1021/acs.jcim.3c00647 (accessed 11-16-2023). PMC11005044 37552222

[ref29] Sivula T.; Yetukuri L.; Kalliokoski T.; Kasnanen H.; Poso A.; Pohner I. Machine Learning-Boosted Docking Enables the Efficient Structure-Based Virtual Screening of Giga-Scale Enumerated Chemical Libraries. J. Chem. Inf. Model. 2023, 63, 5773–5783. 10.1021/acs.jcim.3c01239. 37655823 PMC10523430

[ref30] Gentile F.; Fernandez M.; Ban F.; Ton A. T.; Mslati H.; Perez C. F.; Leblanc E.; Yaacoub J. C.; Gleave J.; Stern A.; et al. Automated Discovery of Noncovalent Inhibitors of SARS-Cov-2 Main Protease by Consensus Deep Docking of 40 Billion Small Molecules. Chem. Sci. 2021, 12, 15960–15974. 10.1039/D1SC05579H. 35024120 PMC8672713

[ref31] Desaphy J.; Bret G.; Rognan D.; Kellenberger E. sc-PDB: A 3D-Database of Ligandable Binding Sites--10 Years On. Nucleic Acids Res. 2015, 43, D399–404. 10.1093/nar/gku928. 25300483 PMC4384012

[ref32] Bostrom J.; Brown D. G.; Young R. J.; Keseru G. M. Expanding the Medicinal Chemistry Synthetic Toolbox. Nat. Rev. Drug Discovery 2018, 17, 709–727. 10.1038/nrd.2018.116. 30140018

[ref33] Rarey M.; Kramer B.; Lengauer T.; Klebe G. A Fast Flexible Docking Method Using an Incremental Construction Algorithm. J. Mol. Biol. 1996, 261, 470–489. 10.1006/jmbi.1996.0477. 8780787

[ref34] Jones G.; Willett P.; Glen R. C.; Leach A. R.; Taylor R. Development and Validation of a Genetic Algorithm for Flexible Docking. J. Mol. Biol. 1997, 267, 727–748. 10.1006/jmbi.1996.0897. 9126849

[ref35] Korb O.; Stutzle T.; Exner T. E. Empirical Scoring Functions for Advanced Protein-Ligand Docking with Plants. J. Chem. Inf. Model. 2009, 49, 84–96. 10.1021/ci800298z. 19125657

[ref36] Li J.; Li C.; Sun J.; Palade V. RDPSOVina: The Random Drift Particle Swarm Optimization for Protein-Ligand Docking. J. Comput. Aided Mol. Des. 2022, 36, 415–425. 10.1007/s10822-022-00455-4. 35532815

[ref37] Jain A. N. Surflex-Dock 2.1: Robust Performance from Ligand Energetic Modeling, Ring Flexibility, and Knowledge-Based Search. J. Comput. Aided Mol. Des. 2007, 21, 281–306. 10.1007/s10822-007-9114-2. 17387436

[ref38] Verdonk M. L.; Giangreco I.; Hall R. J.; Korb O.; Mortenson P. N.; Murray C. W. Docking Performance of Fragments and Druglike Compounds. J. Med. Chem. 2011, 54, 5422–5431. 10.1021/jm200558u. 21692478

[ref39] Chachulski L.; Windshugel B. Leads-Frag: A Benchmark Data Set for Assessment of Fragment Docking Performance. J. Chem. Inf. Model. 2020, 60, 6544–6554. 10.1021/acs.jcim.0c00693. 33289563

[ref40] Marcou G.; Rognan D. Optimizing Fragment and Scaffold Docking by Use of Molecular Interaction Fingerprints. J. Chem. Inf. Model. 2007, 47, 195–207. 10.1021/ci600342e. 17238265

[ref41] Hartenfeller M.; Zettl H.; Walter M.; Rupp M.; Reisen F.; Proschak E.; Weggen S.; Stark H.; Schneider G. DOGS: Reaction-Driven De Novo Design of Bioactive Compounds. PLoS Comput. Biol. 2012, 8, e1002380 10.1371/journal.pcbi.1002380. 22359493 PMC3280956

[ref42] Sommer K.; Flachsenberg F.; Rarey M. NAOMInext - Synthetically Feasible Fragment Growing in a Structure-Based Design Context. Eur. J. Med. Chem. 2019, 163, 747–762. 10.1016/j.ejmech.2018.11.075. 30576905

[ref43] Moroz Y. S. 2022q3-4 Real Database Reagents, Personal Communication, 2023.

[ref44] Malamas M. S.; Manas E. S.; McDevitt R. E.; Gunawan I.; Xu Z. B.; Collini M. D.; Miller C. P.; Dinh T.; Henderson R. A.; Keith J. C. Jr.; Harris H. A. Design and Synthesis of Aryl Diphenolic Azoles as Potent and Selective Estrogen Receptor-Beta Ligands. J. Med. Chem. 2004, 47, 5021–5040. 10.1021/jm049719y. 15456246

[ref45] Da Silva F.; Desaphy J.; Rognan D. IChem: A Versatile Toolkit for Detecting, Comparing, and Predicting Protein-Ligand Interactions. ChemMedChem. 2018, 13, 507–510. 10.1002/cmdc.201700505. 29024463 PMC5901026

[ref46] Openeye Scientific Software, Sante Fe, NM, U.S.A. https://www.eyesopen.com/ (accessed 11-16-2023).

[ref47] Penner P.; Guba W.; Schmidt R.; Meyder A.; Stahl M.; Rarey M. The Torsion Library: Semiautomated Improvement of Torsion Rules with SMARTScompare. J. Chem. Inf. Model. 2022, 62, 1644–1653. 10.1021/acs.jcim.2c00043. 35318851

[ref48] Schneider N.; Lange G.; Hindle S.; Klein R.; Rarey M. A Consistent Description of Hydrogen Bond and Dehydration Energies in Protein-Ligand Complexes: Methods Behind the Hyde Scoring Function. J. Comput. Aided Mol. Des. 2013, 27, 15–29. 10.1007/s10822-012-9626-2. 23269578

[ref49] Fischer A.; Smiesko M.; Sellner M.; Lill M. A. Decision Making in Structure-Based Drug Discovery: Visual Inspection of Docking Results. J. Med. Chem. 2021, 64, 2489–2500. 10.1021/acs.jmedchem.0c02227. 33617246

[ref50] https://www.ebi.ac.uk/chembl/ (accessed 11-16-2023).

[ref51] Chien E. Y. T.; Liu W.; Zhao Q.; Katritch V.; Won Han G.; Hanson M. A.; Shi L.; Newman A. H.; Javitch J. A.; Cherezov V.; Stevens R. C. Structure of the Human Dopamine D3 Receptor in Complex with a D2/D3 Selective Antagonist. Science 2010, 330, 1091–1095. 10.1126/science.1197410. 21097933 PMC3058422

[ref52] Maramai S.; Gemma S.; Brogi S.; Campiani G.; Butini S.; Stark H.; Brindisi M. Dopamine D3 Receptor Antagonists as Potential Therapeutics for the Treatment of Neurological Diseases. Front. Neurosci. 2016, 10, 451 10.3389/fnins.2016.00451. 27761108 PMC5050208

[ref53] Hopkins A. L.; Groom C. R.; Alex A. Ligand Efficiency: A Useful Metric for Lead Selection. Drug Discovery Today 2004, 9, 430–431. 10.1016/S1359-6446(04)03069-7. 15109945

[ref54] Cache Challenge: Critical Assessment of Computational Hit-Finding Experiments, https://cache-challenge.org/, (accessed 11-16-2023).

[ref55] sc-PDB: An Annotated Database of Druggable Binding Sites from the Protein Databank, http://bioinfo-pharma.u-strasbg.fr/scPDB/, (accessed 11-16-2023). 10.1021/ci050372x 16563002

[ref56] Lewell X. Q.; Judd D. B.; Watson S. P.; Hann M. M. Recap--Retrosynthetic Combinatorial Analysis Procedure: A Powerful New Technique for Identifying Privileged Molecular Fragments with Useful Applications in Combinatorial Chemistry. J. Chem. Inf. Comput. Sci. 1998, 38, 511–522. 10.1021/ci970429i. 9611787

[ref57] Clark M.; Cramer R. D. III.; Van Opdenbosch N. Validation of the General Purpose Tripos 5.2 Force Field. J. Comput. Chem. 1989, 10, 982–1012. 10.1002/jcc.540100804.

[ref58] ADFR Software Suite Downloads, https://ccsb.scripps.edu/adfr/downloads/ (accessed 11-16-2023).

[ref59] Enamine Building Blocks Catalog. https://enamine.net/building-blocks/building-blocks-catalog (accessed 03-25-2023).

[ref60] Dassault Systèmes Biovia Corp., San Diego, CA. https://www.3ds.com/products-services/biovia/products/data-science/pipeline-pilot/ (accessed 11-16-023).

[ref61] Molecular Networks Gmbh, Nürnberg, Germany, https://mn-am.com/products/corina/ (accessed 11-16-2023).

[ref62] Pike A. C.; Brzozowski A. M.; Hubbard R. E.; Bonn T.; Thorsell A. G.; Engstrom O.; Ljunggren J.; Gustafsson J. A.; Carlquist M. Structure of the Ligand-Binding Domain of Oestrogen Receptor Beta in the Presence of a Partial Agonist and a Full Antagonist. EMBO J. 1999, 18, 4608–4618. 10.1093/emboj/18.17.4608. 10469641 PMC1171535

[ref63] Bietz S.; Urbaczek S.; Schulz B.; Rarey M. Protoss: A Holistic Approach to Predict Tautomers and Protonation States in Protein-Ligand Complexes. J. Cheminform. 2014, 6, 12 10.1186/1758-2946-6-12. 24694216 PMC4019353

[ref64] Halgren T. A. Merck Molecular Force Field. I. Basis, Form, Scope, Parameterization, and Performance of MMMFF94. J. Comput. Chem. 1996, 17, 490–519. 10.1002/(SICI)1096-987X(199604)17:5/6<490::AID-JCC1>3.0.CO;2-P.

[ref65] O’Boyle N. M.; Banck M.; James C. A.; Morley C.; Vandermeersch T.; Hutchison G. R. Open Babel: An Open Chemical Toolbox. J. Cheminform. 2011, 3, 33 10.1186/1758-2946-3-33. 21982300 PMC3198950

