# Supplementary material for: Protein Structure-Based Organic Chemistry-Driven Ligand Design from Ultralarge Chemical Spaces
Source: ACS Cent Sci. 2024 Feb 13;10(3):615–27. doi: 10.1021/acscentsci.3c01521 (PMC10979501; doi:10.1021/acscentsci.3c01521)
Supplement: Supplementary file 1 — oc3c01521_si_001.pdf [file oc3c01521_si_001.pdf]

## SUPPORTING INFORMATION

# Protein structure-based organic chemistry-driven ligand design from ultra-large chemical spaces.

François Sindt,[1] Anthony Seyller,[1] Merveille Eguida,[1,2] and Didier Rognan[1]*

[1]Laboratoire d'innovation thérapeutique, UMR7200 CNRS-Université de Strasbourg, Illkirch, France

[2] current address: Amgen Research Copenhagen, Copenhagen, Denmark

* e-mail: rognan@unistra.fr

**Table of Content**

**Fig. S1.** Set of 12 organic chemistry rules to process specific bonds in sc-PDB ligands and generate building blocks with defined functional groups.

**Fig. 2.** Cumulative size of the accessible chemical space for 36 organic chemistry reactions.

**Fig.S3.** Chemical and topological rules to form a benzoxazole ring.

**Fig. S4.** Chemical and topological rules to form a sulfonamide bond.

**Fig. S5.** Overlap of Erβ SpaceDock hits to ChEMBL and REAL Space.

**Fig. S6.** Chemical and topological rules to form an amide bond.

**Fig. S7.** Overlap of DRD3 SpaceDock hits to ChEMBL and REAL Space.

**Fig. S8.** Binding of six SpaceDock hits to the human dopamine D3 receptor.

**Fig. S9.** Workflow to select reaction-specific reactants from SMARTS strings

**Table S1.** Rules to filter chemical reagents from fragmented sc-PDB ligands.

**Table S2.** Set of 36 organic chemistry reactions to prepare a combinatorial space of 5.5 billions compounds.

**Table S3.** SpaceDock hits as potential estrogen receptor beta agonists.

**Table S4.** Rules to filter commercial reagents for drug-likeness of amides to be synthesized.

**Table S5.** SpaceDock hits as potential dopamine D3 receptor antagonists.

**Table S6.** Parameter settings for PLANTS docking

**Table S7.** Parameter settings for GOLD docking

**Table S8.** Parameter settings for RDPSOVina docking, Surflex-Dock and FlexX docking

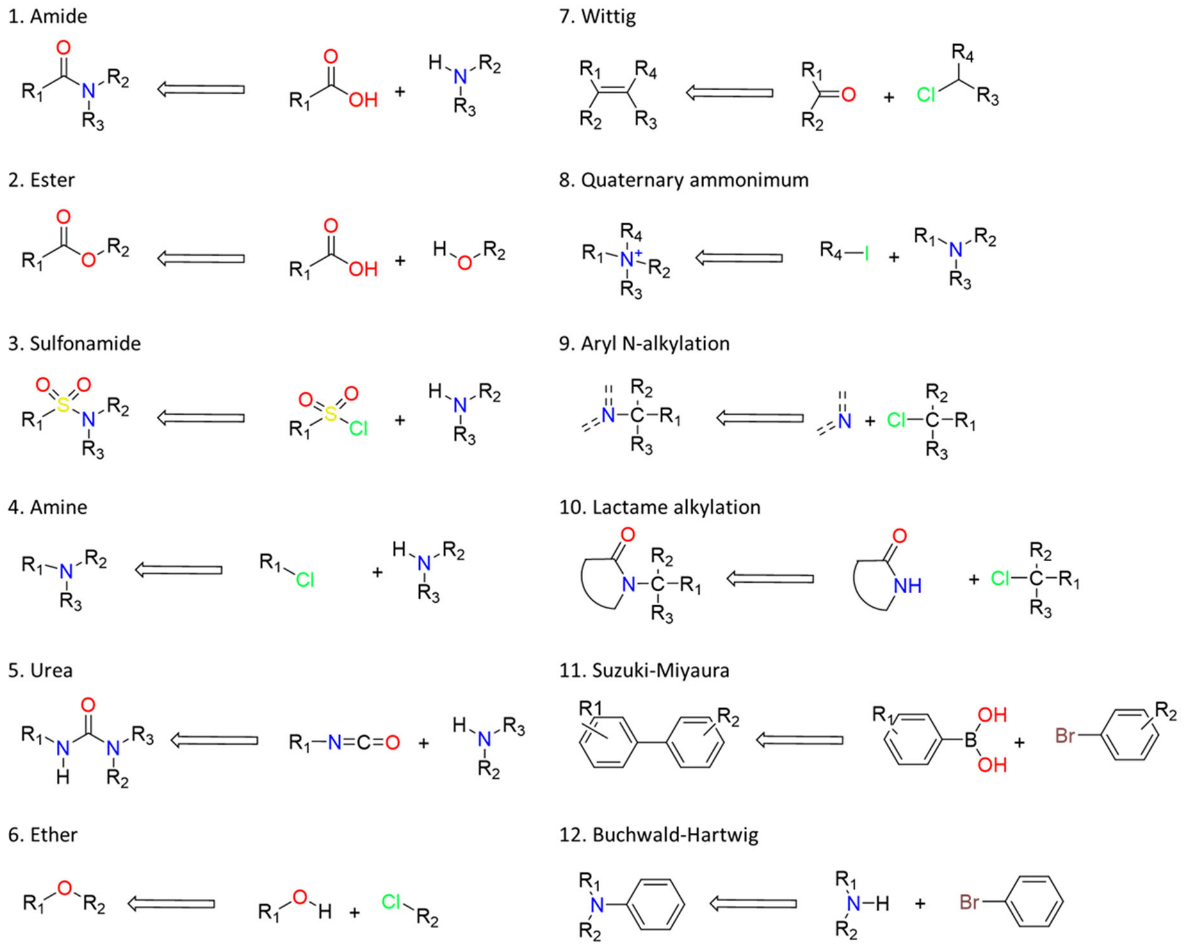


**Fig. S1.** Set of 12 organic chemistry rules to process specific bonds in sc-PDB ligands and generate reactants with defined functional groups.

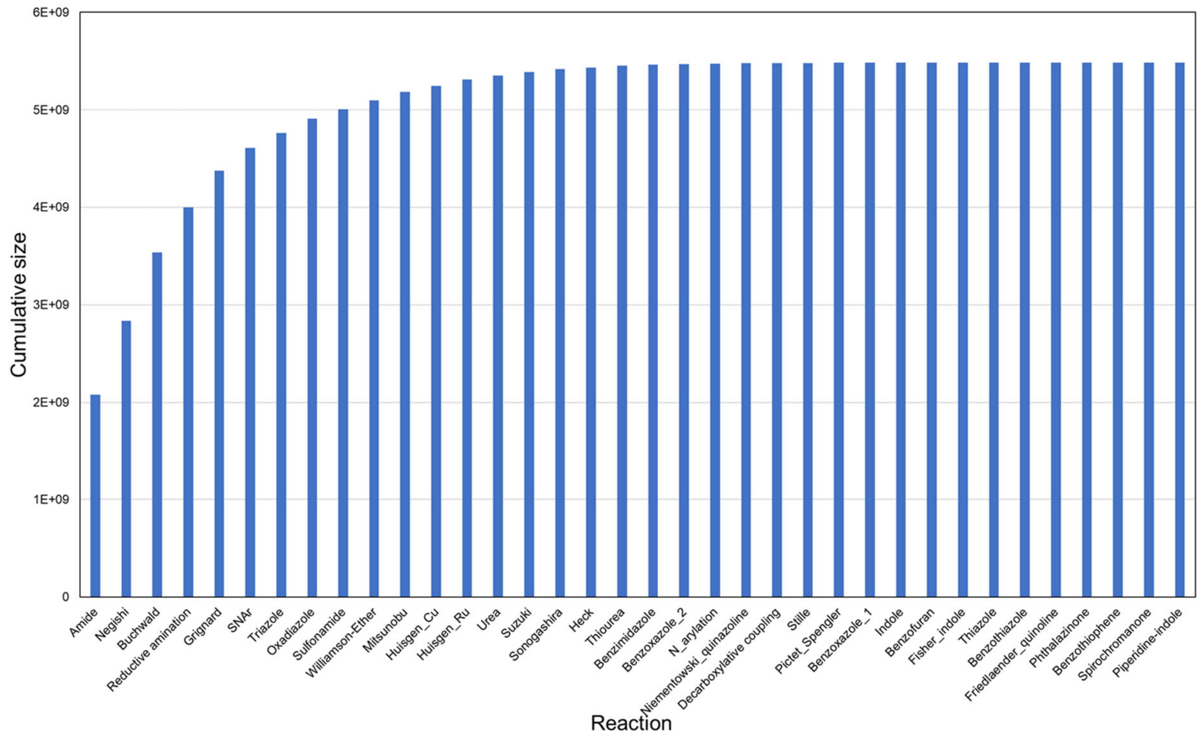


**Fig. S2.** Cumulative size of the accessible chemical space for 36 organic chemistry reactions

| ID | Reaction | Reactant | Tag_atom |
| --- | --- | --- | --- |
| EN300-138860 | Benzoxazole | Aminophenol | 8 9 2 1 |
| ... | | | |
| EN300-19124 | Benzoxazole | Benzaldehyde | 3 2 1 |

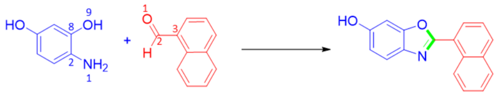


EN300-138860 EN300-19124

1. Reaction eq 'Benzoxazole'

2. Select the proper tags
   for Aminophenol → Reactant eq 'Aminophenol' and tags on C-OH (8, 9) and C-N atoms (2, 1)
   for Benzaldehyde→ Reactant eq 'Benzaldehyde' and tags on CC=O atoms (atoms 3, 2, 1)

3. Check distances/angles/clashes

   cmass-cmass: 5 – 7.5 Å
   $N_1$-$C_2$: 1 - 3 Å
   $O_9$-$C_2$: 1 - 3 Å
   $C_2$-$N_1$-$C_2$: 105 ± 50 deg.
   $C_8$-$O_9$-$C_2$: 105 ± 50 deg.
   Clashes <= 4

4. Add a single bond (type 1)
   between the 2nd tag of the aminophenol (atom 9) and the 2nd tag (atom 2) of the benzaldehyde

5. Add a double bond (type 2)
   between the 4th tag of the aminophenol (atom 1) and 2nd tag (atom 2) of the benzaldehyde

6. Remove exit atoms
   the two hydrogen atoms linked to the 4th tag (atom 1) of the aminophenol
   the hydrogen linked to the 2nd tag (atom 9) of the aminophenol
   the CHO group of the benzaldehyde: hydrogen bonded to the 2nd tag of the benzaldehyde (atom 2) + the last 2 tags (atom 2, 1) of the benzaldehyde

7. Modify atom types
   4th tag of the aminophenol (atom 1) → N.2
   2nd tag of the aminophenol (atom 9) → O.3 with a charge of 0

**Fig. S3.** Chemical and topological rules to form a benzoxazole ring, exemplified by reactants EN300-138860 and EN300-019124. A clash is defined as any short contact (< 2.5 Å) between non-tagged heavy atoms of both reactants.

| ID | Reaction | Reactant | Tag_atom |
|---|---|---|---|
| EN300-62688 | Sulfonamide | Amine | 1 |
| … | | | |
| EN300-81551 | Sulfonamide | Sulfonylchloride | 2 1 |

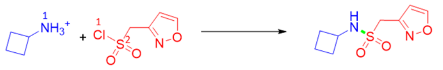


EN300-62688 EN300-81551 EN300-62688_EN300-81551_10

1. Reaction eq 'Sulfonamide'

2. Select the proper tags
   for Amine → Reactant eq 'Amine' and tag on the amine nitrogen atom (atom 1)
   for Sulfonylchloride→ Reactant eq 'Sulfonylchloride' and tags on S, Cl atoms (atoms 2, 1)

3. Check distances/angles/clashes

   cmass-cmass: 5 – 7.5 Å
   $N_1$-$S_2$: 1 - 3 Å
   Clashes <= 4

4. Add a single bond (type 1)
   between the tag of the amine (atom 1) and the 1st tag of the sulfonylchloride (atom 2)

5. Remove exit atoms
   one hydrogen linked to the tag of the amine (atom 1) if nitrogen is not N.4
   two hydrogens linked to the tag of the amine (atom 1) if nitrogen is N.4
   the chlorine corresponding to the 2nd tag of the sulfonylchloride (atom 1)
   → new sulfonamide bond torsion H-N-S-O closest to 180 deg.

6. Modify atom types
   tag of the amine (atom 1) → N.pl3

**Fig. S4.** Chemical and topological rules to form a sulfonamide bond, exemplified by reactants EN300-62688 and EN300-85551. A clash is defined as any short contact (< 2.5 Å) between non-tagged heavy atoms of both reactants.

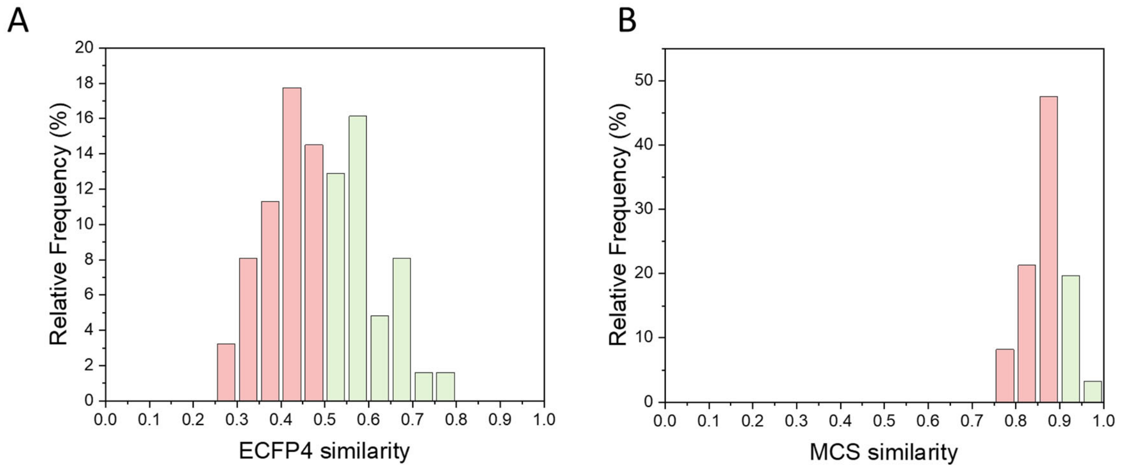


**Fig. S5.** Overlap of SpaceDock hits to ChEMBL and REAL Space. **A)** Chemical similarity of SpaceDock hits to known ERβ ligand from ChEMBL. Similarity is expressed by the Tanimoto coefficient of ECFP4 circular fingerprints, computed with Pipeline Pilot v.22.1.0.2935. Hits are considered similar to ChEMBL compounds if the similarity is greater than or equal to 0.50 (green bars). **B)** Maximum common substructure (MCS) similarity of SpaceDock hits to 36 billion on-demand compounds from REAL space, computed with SpaceMACS v.0.9.2. Hits are considered to intersect Enamine REAL space if the MCS similarity is greater than or equal to 0.90 (green bars).

| ID | Reaction | Reactant | Tag_atom |
|---|---|---|---|
| EN300-62688 | Amide | Amine | 1 |
| ... | | | |
| EN300-23735 | Amide | Carboxylic acid | 2 1 |

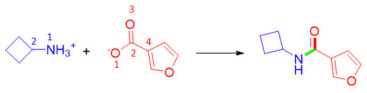


EN300-62688 EN300-23735 EN300-62688_EN300-23735_1

1. Reaction eq 'Amide'

2. Select the proper tags
   for Amine → Reactant eq 'Amine' and tag on the amine nitrogen atom (atom 1)
   for Carboxylic acid → Reactant eq 'Carboxylic acid' and tags on C.2 and one of the two O.co2 atoms (atoms 2, 1 or 3)

3. Check distances/angles/clashes

cmass-cmass: 5-7.5 Å
$N_1$-$C_2$: 1 - 3 Å
$C_2$-$N_1$-$C_2$ : 105 ± 50 deg.
$C_4$-$C_2$-$N_1$ : 105 ± 50 deg.
$C_2$-$N_1$-$C_2$ + $C_4$-$C_2$-$N_1$ : 205 ± 60 deg.
Clashes <= 4

4. Add an amide bond (type am)
   between the tag of the amine (atom 1) and the 1st tag (atom 2) of the carboxylic acid

5. Remove exit atoms
   one hydrogen linked to the tag of the amine (atom 1) if nitrogen is not N.4
   two hydrogens linked to the tag of the amine (atom 1) if nitrogen is N.4
   one of the 2 O.co2 atoms linked to the first tag (atom 2) of the carboxylic acid
   → *new amide bond torsion closest to 180 deg.*

6. Modify atom types
   tag of the amine (atom 1) → N.am
   remaining O.co2 atom → O.2

**Fig. S6.** Chemical and topological rules to form an amide bond, exemplified by reactants EN300-62688 and EN300-23735. A clash is defined as any short contact (< 2.5 Å) between non-tagged heavy atoms of both reactants.

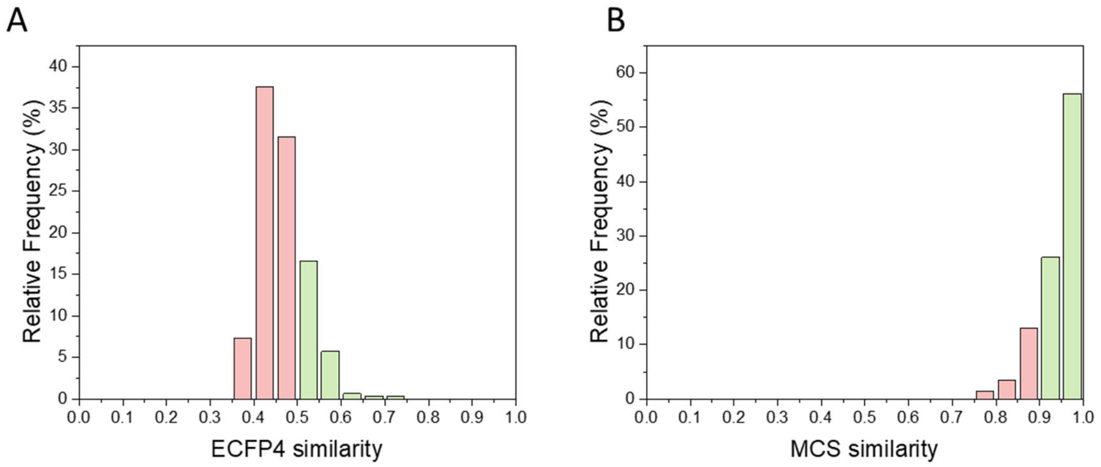


**Fig. S7.** Overlap of SpaceDock hits to ChEMBL and REAL Space. **A)** Chemical similarity of SpaceDock hits to known DRD2/DRD3 ligand from ChEMBL. Similarity is expressed by the Tanimoto coefficient of ECFP4 circular fingerprints, computed with Pipeline Pilot v.22.1.0.2935. Hits are considered similar to ChEMBL compounds if the similarity is greater than or equal to 0.50 (green bars). **B)** Maximum common substructure (MCS) similarity of SpaceDock hits to 36 billion on-demand compounds from REAL space, computed with SpaceMACS v.0.9.2. Hits are considered to intersect Enamine REAL space if the MCS similarity is greater than or equal to 0.90 (green bars).

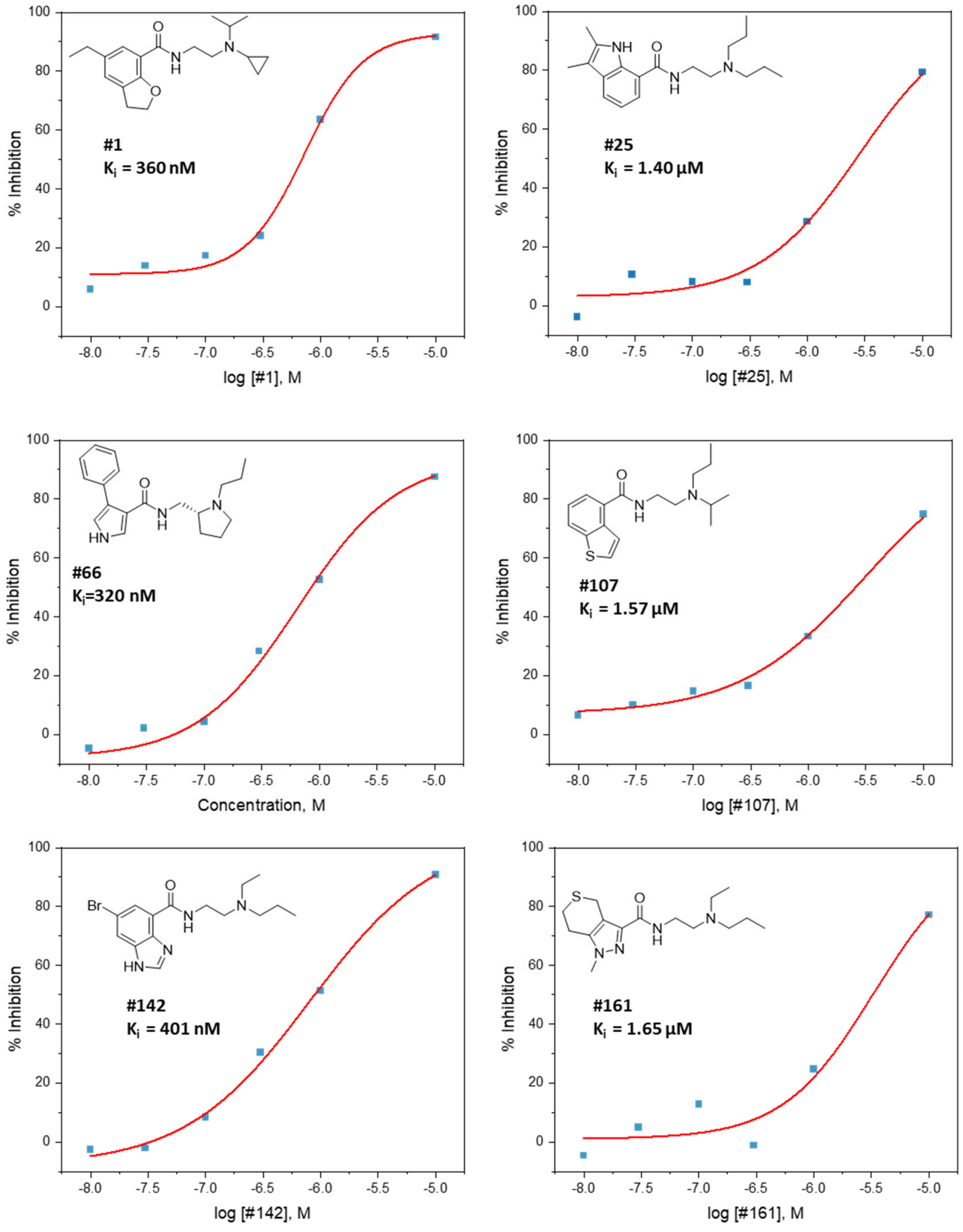


**Fig. S8.** Binding of six SpaceDock hits to the human dopamine D3 receptor, expressed by the percentage of inhibition of [3H]-methylspiperone binding to human recombinant DRD3 in CHO cells (Eurofins-Discovery assay #48). Inhibition constants ($K_i$) were determined from six concentrations dose-response curves.

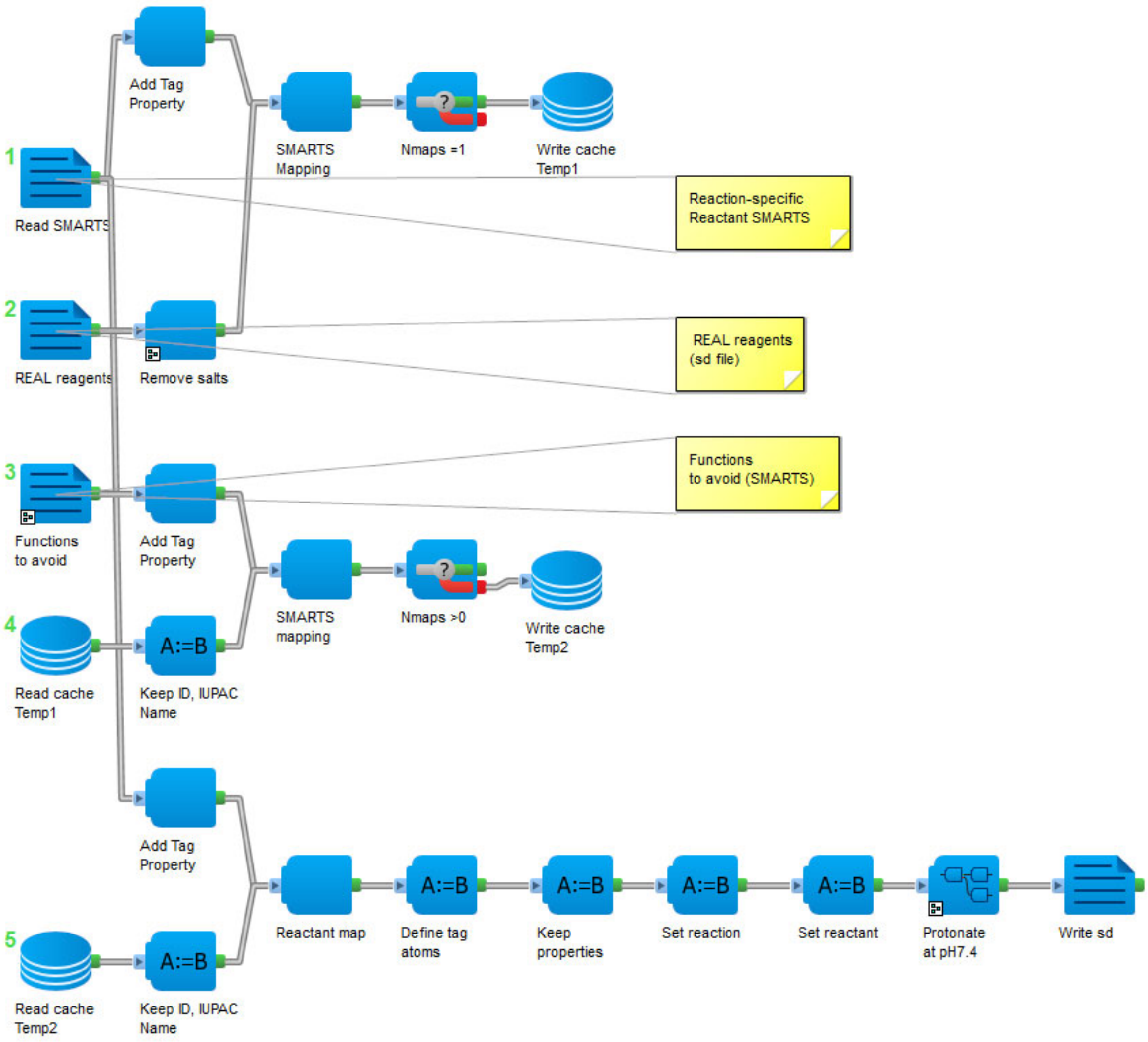


**Fig. S9.** PipelinePilot workflow to select reaction-specific reactants from SMARTS strings.

**Table S1.** Rules to filter chemical reagents from fragmented sc-PDB ligands.

| Rule | Lower limit | Upper limit |
|---|---|---|
| Non-covalent interactions to the target protein | 4 (1 polar) | - |
| Number of heavy atoms | 3 | 23 |
| Number of rotatable bonds | 0 | 6 |
| Heteroatom to carbon ratio | 0.05 | 4.5 |
| Number of aromatic rings | 0 | 3 |
| Number of fused cycles | 0 | 2 |

**Table S2.** Set of 36 organic chemistry reactions to prepare bespoke combinatorial spaces.

| Reaction | Reactant 1 | Reactant 2 | Disallowed functions | N1 | N2 | Products | Nsteps | Ring formation |
|---|---|---|---|---|---|---|---|---|
| Amide | Amines (prim, sec) | Carboxylic acids | Esters, alcohols, acyl chlorides, sulfonyl chlorides, alkyl halides | 62,710 | 33,173 | 2,080,278,830 | 1 | no |
| Negishi[a] | Halides (Cl, Br, I) | Halides (Cl, Br, I) | Amines (prim, sec), alcohols | 27,447 | 27,447 | 753,337,809 | 2 | no |
| Buchwald | Amines (prim, sec) | Aryl halides (Cl, Br, I) | Alcohols, carboxylic acids, acyl chlorides, sulfonyl chlorides, alkyl halides | 56,133 | 12,520 | 702,785,160 | 1 | no |
| Reductive amination | Amines (prim, sec) | Ketones, Aldehydes | Acyl chlorides, sulfonyl chlorides, alkyl halides | 34,466 | 13,453 | 463,671,098 | 2 | no |
| Grignard[b] | Nitriles, ketones , aldehydes | Halides (Cl, Br, I) | Carboxylic acids, Esters, acyl chlorides, sulfonyl chlorides | 14,845 | 25,194 | 374,004,930 | 2 | no |
| SNAr | Amines (prim, sec) | Aryl halides (Cl, F) | Carboxylic acids, alcohols, esters, acyl chlorides, sulfonylchlorides, alkyl halides | 62,980 | 3,743 | 235,734,140 | 1 | no |
| Triazole[c] | Nitriles | Carboxylic acids/esters | Amines (prim, sec), alcohols, acyl chlorides, sulfonyl chlorides, alkyl halides | 4,196 | 36,746 | 154,186,216 | 2 | yes |
| Oxadiazole[d] | Nitriles | Carboxylic acids | Amines (prim, sec), alcohols, acyl chlorides, sulfonyl chlorides, alkyl halides | 4,305 | 33,568 | 144,510,240 | 2 | yes |
| Sulfonamide | Amines (prim, sec) | Sulfonyl chlorides | Carboxylic acids, alcohols, esters, acyl chlorides, alkyl halides | 69,568 | 1,374 | 95,586,432 | 1 | no |
| Williamson-Ether | Alcohols | Halides (Cl, Br, I) | Amines (prim, sec), Carboxylic acids, esters, acyl chlorides, sulfonyl chlorides | 10,959 | 8,449 | 92,592,591 | 1 | no |
| Mitsunobu | Alcohols | Imides, Phenols, Sulfonamides | Carboxylic acids, acyl chlorides, sulfonylchlorides, alkyl halides | 15,986 | 5,354 | 85,589,044 | 1 | no |
| Huisgen_Cu[e] | Alkynes | Halides, Alcohols | Dienes | 2,253 | 28,287 | 63,730,611 | 2 | yes |
| Huisgen_Ru[e] | Alkynes | Halides, Alcohols | Dienes | 2,253 | 28,287 | 63,730,611 | 2 | yes |
| Urea | Amines (prim, sec) | Isocyanates | Alcohols, acyl chlorides, sulfonyl chlorides, carboxylic acids, esters, alkyl chlorides | 62,710 | 676 | 42,391,960 | 1 | no |
| Suzuki | Boronic acids, esters | Aryl halides (Cl, Br, I) | Vinyls, alkynes | 1,508 | 23,351 | 35,213,308 | 1 | no |
| Sonogashira | Alkynes | Halides (Br, I) | Allenes, dienes, boronates, vinyls | 1,752 | 16,527 | 28,955,304 | 1 | no |
| Heck | Halides (Cl, Br, I) | Vinyls | Boronates,alkynes | 27,833 | 672 | 18,703,776 | 1 | no |
| Thiourea | Amines (prim, sec) | Isothiocyanates | Alcohols, acyl chlorides, sulfonyl chlorides, carboxylic acids, esters, alkyl halides | 62,710 | 259 | 16,241,890 | 1 | no |
| Benzimidazole | Diaminoaryl | Carboxylic acids/esters/aldehydes | Alcohols, acyl chlorides, sulfonyl chlorides, alkyl halides | 237 | 51,197 | 12,133,689 | 1 | yes |
| Benzoxazole_2 | 1-hydroxy,2-aminoheteroaromatic | Carboxylic acids | Alcohols, acyl chlorides, sulfonyl chlorides, alkyl halides, esters | 156 | 38,680 | 6,034,080 | 1 | yes |
| N_arylation | Boronic acids, esters | Het-NH | Alkyl chlorides, acyl chlorides, sulfonyl chlorides, Aryl halides | 1,532 | 2,899 | 4,441,268 | 1 | no |
| Niementowski_quinazoline | Anthranilic acids | Amides | Acyl chlorides, sulfonyl chlorides, alkyl halides, alcohols | 231 | 11,631 | 2,686,761 | 1 | yes |
| Decarboxylative coupling | Halides (Cl, Br, I) | Benzoic acids | Alcohols, acyl chlorides, sulfonyl chlorides | 21,240 | 101 | 2,145,240 | 1 | no |
| Stille[f] | Aryl halides (Cl, Br, I) | Vinyl_aryl halides (Br, I) | Boronates, alkynes | 13,535 | 144 | 1,949,040 | 2 | no |
| Pictet_Spengler | Phenethylamines | Aldehydes | Ketones, acyl chlorides, sulfonyl chlorides, alkyl halides | 361 | 5,121 | 1,848,681 | 1 | yes |

| | | | | | | | | |
|---|---|---|---|---|---|---|---|---|
| Benzoxazole_1 | Anthranilic acids | Benzaldehydes | Alcohols, acyl chlorides, sulfonyl chlorides, alkyl halides | 148 | 4,028 | 596,144 | 1 | yes |
| Indole | Anilines | Alkynes | Carboxylic acids, Acyl chlorides, sulfonyl chlorides, alkyl halides | 224 | 2,267 | 507,808 | 1 | yes |
| Benzofuran | Phenols | Alkynes | Acyl chlorides, sulfonyl chlorides, alkyl halides | 183 | 2,507 | 458,781 | 1 | yes |
| Fisher_indole | Phenylhydrazines | Ketones | Acyl chlorides, sulfonyl chlorides, alkyl halides, Carboxylic acids | 151 | 2,789 | 421,139 | 1 | yes |
| Thiazole | Halogenoketones | Thioamides | | 266 | 709 | 188,594 | 1 | yes |
| Benzothiazole | Thiophenols | Aldehydes | Acyl chlorides, sulfonyl chlorides, alkyl halides | 8 | 5,138 | 41,104 | 1 | yes |
| Friedlaender_quinoline | Aminoacetophenones | Ketones | Carboxylic acids, Acyl chlorides, sulfonyl chlorides, alkyl halides | 13 | 2,789 | 36,257 | 1 | yes |
| Phthalazinone | Benzoic acids | Hydrazines | alcohols, acyl chlorides, sulfonyl chlorides, alkyl halides | 30 | 921 | 27,630 | 1 | yes |
| Benzothiophene | Phenylthioethers | Alkynes | | 11 | 2,507 | 27,577 | 1 | yes |
| Spirochromanone | Hydroxyacetophenones | Ketones | | 67 | 267 | 17,889 | 1 | no |
| Piperidine-indole | Indoles | Piperidones | Amines (prim, sec), acyl chlorides, sulfonyl chlorides, alkyl halides | 118 | 111 | 13,098 | 1 | no |

[a] formation a Zn halide on one of the two reactants, [b] Reactant 2 has to be tranformed into Grignard reagent (RMgX) first, [c] additional nucleophilic substitution with hydrazine, [d] convert nitrile to amidoxime by hydroxylamine, [e] convert halide/alcohol into azide with $NaN_3$, [f] Reactant 2 has to be transformed into organotin reagent.

**Table S3.** SpaceDock hits as potential estrogen receptor beta agonists. The ground truth ligand (WAY-338) is highlighted in green.

| Rank | SpaceDock SMILES | IFP_full[a] | IFP_polar[b] | HYDE[c] | Tc-MCS[d] | REAL SMILES[e] | Tc-ECFP4[f] |
|---|---|---|---|---|---|---|---|
| 1 | O1c2c(N=C1c3c(cc(N)cc3C)C)ccc(c2)CO | 0.722 | 0.667 | -46.78 | 0.81 | O1c2c(N=C1c3c(cc(cc3C)C)C)cccc2 | 0.43 |
| 2 | O1c2c(N=C1c3c(cc(N)cc3C)C)ccc(O)c2C | 0.778 | 0.667 | -43.85 | 0.81 | O1c2c(N=C1c3c(cc(cc3C)C)C)cccc2 | 0.49 |
| 3 | O1c2c(N=C1c3c(OC)c4c(NC=C4)cc3)ccc(c2)CO | 0.882 | 1.000 | -38.63 | 0.82 | O1c2c(N=C1c3cc4c(NC=C4)cc3)cccc2 | 0.44 |
| 4 | O1c2c(N=C1c3cc(NC)ccc3)ccc(c2)CO | 0.722 | 0.667 | -37.95 | 0.89 | O1c2c(N=C1c3cc(NC)ccc3)cccc2 | 0.47 |
| 5 | S(=O)(=O)(NC1C[C@@H]2C(O)[C@H](C1)CC2)CCCC#N | 0.765 | 0.667 | -37.62 | 0.89 | S(=O)(=O)(NC1CCC(O)CC1)CCCC#N | 0.30 |
| 6 | S(=O)(=O)(NC1C[C@@H]2C(O)[C@H](C1)CC2)CCCCC#N | 0.765 | 0.667 | -36.86 | 0.89 | S(=O)(=O)(NC1CCC(O)CC1)CCCCC#N | 0.27 |
| 7 | O1c2c(N=C1c3cc(c(O)cc3)C)ccc(O)c2C | 0.824 | 1.000 | -36.12 | 0.89 | O1c2c(N=C1c3cc(c(O)cc3)C)cccc2 | 0.55 |
| 8 | S(=O)(=O)(N1C([C@@H](CO)CC1)(C)C)CCCC#N | 0.647 | 0.667 | -36.03 | 1.00 | S(=O)(=O)(N1C(C(CO)CC1)(C)C)CCCC#N | 0.31 |
| 9 | Oc1ccc2nc(oc2c1)c3cccc4c(O)cccc34 | 0.647 | 0.667 | -35.59 | 0.90 | O1c2c(N=C1c3c4c(ccc3)cccc4)cccc2 | 1.00 |
| 10 | O1N=Cc2c1ccc(c2)C=3Oc4c(N3)ccc(O)c4C | 0.765 | 0.667 | -34.11 | 0.82 | O1N=Cc2c1ccc(c2)C=3Nc4c(c(C#N)ccc4)C3 | 0.56 |
| 11 | O1c2c(N=C1c3c(O)ccc(c3)CO)ccc(O)c2 | 0.722 | 1.000 | -32.89 | 0.89 | O1c2c(N=C1c3c(O)ccc(c3)C)cccc2 | 0.66 |
| 12 | O1c2c(N=C1c3cc(c(O)c(c3)C)C)ccc(c2)CO | 0.889 | 1.000 | -32.52 | 0.90 | O1c2c(N=C1c3cc(c(O)c(c3)C)C)cccc2 | 0.51 |
| 13 | O1c2c(N=C1c3cc(NC)ccc3)ccc(O)c2C | 0.882 | 0.667 | -32.50 | 0.89 | O1c2c(N=C1c3cc(NC)ccc3)cccc2 | 0.52 |
| 14 | O1c2c(N=C1c3cc(ccc3)[C@H](O)C)ccc(O)c2 | 0.882 | 1.000 | -32.48 | 0.89 | O1c2c(N=C1c3cc(ccc3)CC)cccc2 | 0.61 |
| 15 | O1c2c(N=C1c3cc4c(NC=C4)cc3)ccc(c2)CO | 0.882 | 1.000 | -32.40 | 0.90 | O1c2c(N=C1c3cc4c(NC=C4)cc3)cccc2 | 0.47 |
| 16 | O1c2c(N=C1c3cc4c(NN=C4)cc3)ccc(O)c2C | 0.941 | 1.000 | -32.12 | 0.86 | O1c2c(N=C1C3=CC=4C(=NN(C4)C)C=C3)cccc2 | 0.46 |
| 17 | O1c2c(N=C1c3cc(O)c(O)cc3)c4c(NC=C4)cc2 | 0.722 | 0.750 | -31.76 | 0.81 | O1c2c(N=C1c3cc(O)c(OC)cc3)cccc2 | 0.61 |
| 18 | O1c2c(N=C1c3c(O)cc(O)cc3C)ccc(O)c2C | 0.722 | 1.000 | -31.57 | 0.81 | O1c2c(N=C1c3c(cc(O)c(c3)C)C)cccc2 | 0.53 |
| 19 | O1c2c(N=C1c3c4c(NC=C4)ccc3)ccc(c2)CO | 0.647 | 0.667 | -31.41 | 0.90 | O1c2c(N=C1c3c4c(NC=C4)ccc3)cccc2\| | 0.52 |
| 20 | O1c2c(N=C1c3cc(ccc3)[C@H](O)C)ccc(c2)CO | 0.882 | 1.000 | -30.87 | 0.85 | O1c2c(N=C1c3cc(ccc3)CC)cccc2 | 0.42 |
| 21 | S(=O)(=O)(N1C(C[C@H](CO)C1)(C)C)CCCC#N | 0.706 | 0.667 | -30.80 | 0.94 | S(=O)(=O)(N1C(CC(CO)C1)C)CCCC#N | 0.30 |
| 22 | O1c2c(N=C1c3cc(ccc3)[C@H](O)C)ccc(O)c2C | 0.941 | 1.000 | -29.94 | 0.85 | O1c2c(N=C1c3cc(ccc3)CC)cccc2 | 0.48 |
| 23 | S(=O)(=O)(NCc1cc(F)c(N2CCC(O)CC2)cc1)NC | 0.684 | 1.000 | -29.76 | 1.00 | S(=O)(=O)(NCc1cc(F)c(N2CCC(O)CC2)cc1)NC | 0.39 |
| 24 | O1c2c(N=C1C3=CN(C(C#N)=C3)C)ccc(O)c2C | 0.824 | 0.667 | -29.69 | 0.76 | N#Cc1c2c(NC(C3=CNC(C#N)=C3)=C2 | 0.47 |
| 25 | O1c2c(N=C1c3ccc(O)cc3)ccc(O)c2 | 0.706 | 1.000 | -28.69 | 0.94 | O1c2c(N=C1c3ccc(O)cc3)cccc2 | 1.00 |
| 26 | O1c2c(N=C1c3cc(O)c(O)cc3)ccc(O)c2C | 0.611 | 1.000 | -28.40 | 0.85 | O1c2c(N=C1c3cc(O)c(OC)cc3)cccc2 | 0.66 |
| 27 | S(=O)(=O)(N1c2c(cccc2)[C@H](O)CC1)CCCC#N | 0.647 | 0.667 | -28.12 | 0.95 | S(=O)(=O)(N1c2c(cccc2)CCC1)CCCC#N | 0.36 |

| | | | | | | | |
|---|---|---|---|---|---|---|---|
| 28 | O1c2c(N=C1c3cc(c(O)cc3)C)ccc(c2)CO | 0.941 | 1.000 | -27.82 | 0.89 | O1c2c(N=C1c3cc(c(O)cc3)C)cccc2 | 0.55 |
| 29 | O1c2c(N=C1c3ccc(O)cc3)ccc(O)c2C | 0.824 | 1.000 | -27.56 | 0.89 | O1c2c(N=C1c3ccc(O)cc3)cccc2 | 0.59 |
| 30 | Clc1c(ccc(N)c1)C=2Oc3c(N2)ccc(O)c3 | 0.765 | 0.667 | -26.60 | 0.89 | Clc1c(cccc1)C=2Oc3c(N2)cccc3 | 0.67 |
| 31 | Clc1c2N=CNc2ccc1C=3Oc4c(N3)ccc(c4)CO | 0.765 | 1.000 | -25.88 | 0.86 | O1c2c(N=C1c3cc4N=CNc4cc3)cccc2 | 0.43 |
| 32 | Clc1c(N)ccc(c1)C=2Oc3c(N2)ccc(O)c3C | 0.882 | 0.667 | -25.65 | 0.84 | Clc1cc(ccc1)C=2Oc3c(N2)cccc3 | 0.59 |
| 33 | O1c2c(N=C1c3ccc(O)cc3)ccc(c2)CO | 0.824 | 1.000 | -25.51 | 0.89 | O=C(O)c1cc2OC(=Nc2cc1)c3ccccc3 | 0.70 |
| 34 | S(=O)(=O)(Nc1nccc(O)c1C)CCCC#N | 0.765 | 0.667 | -25.50 | 0.84 | S(=O)(=O)(N(c1nccc(OC)c1)C)CCCC#N | 0.38 |
| 35 | Brc1nc(ccc1N)C=2Oc3c(N2)ccc(c3)CO | 0.824 | 1.000 | -25.47 | 0.79 | O1c2c(N=C1c3ncccc3)cccc2 | 0.42 |
| 36 | Clc1c(N)ccc(c1)C=2Oc3c(N2)ccc(O)c3 | 0.882 | 1.000 | -25.14 | 0.89 | Clc1cc(ccc1)C=2Oc3c(N2)cccc3 | 0.78 |
| 37 | Clc1c(O)cccc1C=2Oc3c(N2)ccc(c3)CO | 0.667 | 1.000 | -24.71 | 0.86 | Clc1c(O)cccc1C2=Nc3c(N2)ccc(c3)C(=O)O | 0.54 |
| 38 | O1c2c(N=C1c3c4c(NC=C4)ccc3)ccc(O)c2C | 0.824 | 1.000 | -23.95 | 0.90 | O1c2c(N=C1c3c4c(NC=C4)ccc3)cccc2 | 0.52 |
| 39 | O1c2c(N=C1c3cc4N=CNc4cc3)ccc(c2)CO | 0.706 | 1.000 | -22.75 | 0.90 | O1c2c(N=C1c3cc4N=CNc4cc3)cccc2 | 0.44 |
| 40 | S(=O)(=O)(N1[C@@H](C[C@H](O)C[C@@H]1C)C)CCCC#N | 0.611 | 0.667 | -22.00 | 0.94 | S(=O)(=O)(N1C(CC(O)CC1)C)CCCC#N | 0.30 |
| 41 | O1c2c(N=C1c3c(O)ccc(c3)CO)ccc(c2)CO | 0.941 | 1.000 | -21.74 | 0.85 | O1c2c(N=C1c3c(O)ccc(c3)C)cccc2 | 0.53 |
| 42 | O1c2c(N=C1c3c(O)c(O)ccc3)ccc(c2)CO | 0.824 | 1.000 | -21.36 | 0.85 | O=C(O)c1cc2OC(=Nc2cc1)c3ccccc3 | 0.71 |
| 43 | O1c2c(N=C1c3cc4c(NN=C4)cc3)ccc(c2)CO | 0.824 | 1.000 | -21.02 | 0.86 | O=C(O)c1cc2N=C(Nc2cc1)c3cc4c(NN=C4)cc3 | 0.43 |
| 44 | Clc1c(ccc(N)c1)C=2Oc3c(N2)ccc(c3)CO | 0.824 | 1.000 | -20.84 | 0.85 | O=C(O)c1cc2OC(=Nc2cc1)c3ccccc3 | 0.49 |
| 45 | O1c2c(N=C1c3c(O)ccc(O)c3)ccc(c2)CO | 0.882 | 1.000 | -20.48 | 0.85 | O=C(O)c1cc2OC(=Nc2cc1)c3ccccc3 | 0.61 |
| 46 | Clc1c(O)c(cc(c1)C=2Oc3c(N2)ccc(c3)CO)C | 0.889 | 1.000 | -20.02 | 0.90 | Clc1c(O)c(cc(c1)C=2Oc3c(N2)cccc3)C | 0.53 |
| 47 | S(=O)(=O)(N1C([C@@H](CO)CC1)(C)C)C[C@@H]2OC(=O)NC2 | 0.611 | 0.667 | -19.99 | 0.82 | S(=O)(=O)(N1C(C(CO)CC1)(C)C)CC2(NC(=O)NC2=O)C | 0.35 |
| 48 | ClC=1N=C(N(C1C=2Oc3c(N2)ccc(c3)CO)C)N | 0.824 | 1.000 | -18.84 | 0.79 | O1c2c(N=C1C=3N(C=NC3)C)cccc2 | 0.41 |
| 49 | O1c2c(N=C1c3cc(O)c(O)cc3)ccc(c2)CO | 0.941 | 1.000 | -18.83 | 0.85 | O1c2c(N=C1c3cc(O)c(OC)cc3)cccc2 | 0.69 |
| 50 | O=C(N)c1cc2OC(=Nc2cc1)c3cc(c(O)cc3)C | 0.632 | 0.667 | -18.56 | 0.90 | O=C(N)c1cc2OC(=Nc2cc1)c3ccccc3 | 0.56 |
| 51 | O1c2c(N=C1c3cnc(N)cc3)ccc(c2)CO | 0.706 | 1.000 | -17.56 | 0.85 | S1c2c(N=C1c3cnc(N)cc3)ccc(c2)C(=O)O | 0.44 |
| 52 | Clc1c(cc(O)cc1)C=2Oc3c(N2)ccc(c3)CO | 0.778 | 1.000 | -17.49 | 0.86 | Clc1c(cc(O)cc1)C2=Nc3c(N2)ccc(c3)C(=O)O | 0.58 |
| 53 | O1c2c(N=C1c3c4c(NN=C4)ccc3)ccc(O)c2C | 0.824 | 1.000 | -15.11 | 0.90 | O1c2c(N=C1c3c4c(NN=C4)ccc3)cccc2 | 0.47 |
| 54 | Brc1c(O)cccc1C=2Oc3c(N2)ccc(c3)CO | 0.882 | 1.000 | -14.32 | 0.86 | Brc1c(O)cccc1C2=Nc3c(N2)ccc(c3)C(=O)O | 0.60 |
| 55 | Clc1nc(ccc1N)C=2Oc3c(N2)ccc(c3)CO | 0.941 | 1.000 | -13.48 | 0.81 | Clc1nc(ccc1)C2=Nc3c(N2)ccc(c3)C(=O)O | 0.45 |
| 56 | Clc1c(N)ccc(c1)C=2Oc3c(N2)ccc(c3)CO | 0.941 | 1.000 | -13.47 | 0.86 | Clc1cc(Cl)cc(c1)C=2Oc3c(N2)ccc(c3)C(=O)O | 0.56 |
| 57 | O1c2c(N=C1c3c(c(ncc3)N)C)ccc(c2)CO | 0.882 | 1.000 | -11.21 | 0.84 | O1c2c(N=C1c3c(cncc3)C)cccc2 | 0.43 |

| | | | | | | | |
|---|---|---|---|---|---|---|---|
| 58 | Clc1c(ccc(N)c1)C=2Oc3c(N2)ccc(O)c3C | 0.882 | 1.000 | -10.84 | 0.84 | Clc1c(cccc1)C=2Oc3c(N2)cccc3 | 0.49 |
| 59 | Brc1c(cc(O)cc1)C=2Oc3c(N2)ccc(c3)CO | 0.778 | 1.000 | -9.65 | 0.86 | Brc1c(cc(O)cc1)C2=Nc3c(N2)ccc(c3)C(=O)O | 0.56 |
| 60 | S(=O)(=O)(N1C[C@@](CO)(CCC1)C)CC[C@H]2NC(=O)NC2=O | 0.706 | 1.000 | -8.88 | 1.00 | S(=O)(=O)(N1CC(CO)(CCC1)C)CCC2NC(=O)NC2=O | 0.35 |
| 61 | S(=O)(=O)(N1C(CC[C@H](O)C1)(C)C)CC[C@H]2NC(=O)NC2=O | 0.706 | 1.000 | -8.59 | 0.95 | S(=O)(=O)(N1C(CCC(O)C1)C)CCC2NC(=O)NC2=O | 0.34 |
| 62 | Clc1nc(ccc1N)C=2Oc3c(N2)c4c(NC=C4)cc3 | 0.706 | 0.667 | -4.53 | 0.77 | Clc1nc(ccc1)C2=Nc3c(N2)cc4NC=Nc4c3 | 0.39 |
| 63 | Brc1nc(ccc1N)C=2Oc3c(N2)c4c(NC=C4)cc3 | 0.667 | 0.667 | -4.12 | 0.75 | O1c2c(N=C1c3ncccc3)cccc2 | 0.37 |
| 64 | Brc1nc(ccc1N)C=2Oc3c(N2)ccc(c3)C(=O)N | 0.611 | 0.667 | 0.91 | 0.81 | O=C(N)c1cc2OC(=Nc2cc1)c3ccccc3 | 0.37 |

[a] Interaction fingerprint similarity to the X-ray pose of genistein (all interactions), [b] Interaction fingerprint similarity to the X-ray pose of genistein (h-bonds, ionic bonds), [c] HYDE score (kJ/mol), [d] maximum common substructure similarity to the closest REAL space ligand, [e] SMILES string of the closest ligand in REAL space, [f] ECFP4 similarity to the closest ChEMBL ERβ ligand.

**Table S4.** Rules to filter commercial reagents for drug-likeness of amides to be synthesized.

```
#/**************************************************
********************
#Copyright (C) 2000-2005 by OpenEye Scientific Software, Inc.
#**************************************************
********************/
#This file defines the rules for filtering multi-structure files
based
# on properties and substructure patterns.
MIN_MOLWT 30 "Minimum molecular weight"
MAX_MOLWT 300 "Maximum molecular weight"
MIN_NUM_HVY 4 "Minimum number of heavy atoms"
MAX_NUM_HVY 18 "Maximum number of heavy atoms"
MIN_RING_SYS 0 "Minumum number of ring systems"
MAX_RING_SYS 3 "Maximum number of ring systems"
MIN_RING_SIZE 0 "Minimum atoms in any ring system"
MAX_RING_SIZE 20 "Maximum atoms in any ring system"
MIN_CON_NON_RING 0 "Minimum number of connected
non-ring atoms"
MAX_CON_NON_RING 20 "Maximum number of connected
non-ring atoms"
MIN_FCNGRP 0 "Minimum number of functional groups"
MAX_FCNGRP 20 "Maximum number of functional
groups"
MIN_UNBRANCHED 0 "Minimum number of connected
unbranched non-ring atoms"
MAX_UNBRANCHED 8 "Maximum number of connected
unbranched non-ring atoms"
MIN_CARBONS 5 "Minimum number of carbons"
MAX_CARBONS 40 "Maximum number of carbons"
MIN_HETEROATOMS 2 "Minimum number of heteroatoms"
MAX_HETEROATOMS 20 "Maximum number of
heteroatoms"
MIN_Het_C_Ratio 0.10 "Minimum heteroatom to carbon
ratio"
MAX_Het_C_Ratio 1.0 "Maximum heteroatom to carbon
ratio"
MIN_HALIDE_FRACTION 0.0 "Minimum Halide Fraction"
MAX_HALIDE_FRACTION 0.5 "Maximum Halide Fraction"
#count ring degrees of freedom = (#BondsInRing) - 4 -
(RigidBondsInRing) - (BondsSharedWithOtherRings)
#must be >= 0, from JCAMD 14:251-265,2000.
ADJUST_ROT_FOR_RING true "BOOLEAN for whether to
estimate degrees of freedom in rings"
MIN_ROT_BONDS 0 "Minimum number of rotatable bonds"
MAX_ROT_BONDS 6 "Maximum number of rotatable
bonds"
MIN_RIGID_BONDS 0 "Minimum number of rigid bonds"
MAX_RIGID_BONDS 50 "Maximum number of rigid bonds"
MIN_HBOND_DONORS 0 "Minimum number of hydrogen-
bond donors"
MAX_HBOND_DONORS 6 "Maximum number of hydrogen-
bond donors"
MIN_HBOND_ACCEPTORS 0 "Minimum number of
hydrogen-bond acceptors"
MAX_HBOND_ACCEPTORS 10 "Maximum number of
hydrogen-bond acceptors"
MIN_LIPINSKI_DONORS 0 "Minimum number of hydrogens
on O & N atoms"
MAX_LIPINSKI_DONORS 5 "Maximum number of hydrogens
on O & N atoms"
MIN_LIPINSKI_ACCEPTORS 0 "Minimum number of oxygen &
nitrogen atoms"
MAX_LIPINSKI_ACCEPTORS 10 "Maximum number of oxygen
& nitrogen atoms"
MIN_COUNT_FORMAL_CRG 0 "Minimum number formal
charges"
MAX_COUNT_FORMAL_CRG 3 "Maximum number of
formal charges"
MIN_SUM_FORMAL_CRG -2 "Minimum sum of formal
charges"
MAX_SUM_FORMAL_CRG 2 "Maximum sum of formal
charges"
MIN_CHIRAL_CENTERS 0
"Minimum chiral centers"
MAX_CHIRAL_CENTERS 2
"Maximum chiral centers"
MIN_XLOGP -2.0 "Minimum XLogP"
MAX_XLOGP 6.0 "Maximum XLogP"
#choices are insoluble<poorly<moderately<soluble<very<highly
MIN_SOLUBILITY moderately "Minimum solubility"
PSA_USE_SandP true "Count S and P as polar atoms"
MIN_2D_PSA 0.0 "Minimum 2-Dimensional (SMILES) Polar
Surface Area"
MAX_2D_PSA 150.0 "Maximum 2-Dimensional (SMILES)
Polar Surface Area"
AGGREGATORS true "Eliminate known aggregators"
PRED_AGG false "Eliminate predicted aggregators"
#secondary filters (based on multiple primary filters)
GSK_VEBER true "PSA>140 or >10 rot bonds"
MAX_LIPINSKI 2 "Maximum number of Lipinski violations"
MIN_ABS 0.5 "Minimum probability F>10% in rats"
PHARMACOPIA true "LogP > 5.88 or PSA > 131.6"
ALLOWED_ELEMENTS H,C,N,O,F,S,Cl,Br,I,P,B
ELIMINATE_METALS
Sc,Ti,V,Cr,Mn,Fe,Co,Ni,Cu,Zn,Y,Zr,Nb,Mo,Tc,Ru,Rh,Pd,Ag,Cd
#acceptable molecules must have <= instances of each of the
patterns below
#specific, undesirable functional groups
RULE 0 quinone
RULE 0 pentafluorophenyl_esters
RULE 0 paranitrophenyl_esters
RULE 0 HOBT_esters
RULE 0 triflates
RULE 0 lawesson_s_reagent
RULE 0 phosphoramides
RULE 0 beta_carbonyl_quat_nitrogen
RULE 0 acylhydrazide
RULE 0 cation_C_Cl_I_P_or_S
RULE 0 phosphoryl
RULE 0 alkyl_phosphate
RULE 0 phosphinic_acid
RULE 0 phosphanes
RULE 0 phosphoranes
RULE 0 imidoyl_chlorides
RULE 0 nitroso
RULE 0 N_P_S_Halides
RULE 0 carbodiimide
RULE 0 isonitrile
RULE 0 triacyloxime
RULE 0 cyanohydrins
RULE 0 acyl_cyanides
RULE 0 sulfonylnitrile
RULE 0 phosphonylnitrile
RULE 0 azocyanamides
RULE 0 beta_azo_carbonyl
RULE 0 polyenes
RULE 0 saponin_derivatives
RULE 0 cytochalasin_derivatives
RULE 0 cycloheximide_derivatives
RULE 0 monensin_derivatives
RULE 0 squalestatin_derivatives
#functional groups which often eliminate compounds from
consideration
RULE 0 acid_halide
RULE 0 aldehyde
RULE 0 alkyl_halide
RULE 0 anhydride
```

RULE 0 azide
RULE 0 azo
RULE 0 di_peptide
RULE 0 michael_acceptor
RULE 0 beta_halo_carbonyl
RULE 0 nitro
RULE 0 oxygen_cation
RULE 0 peroxide
RULE 0 phosphonic_acid
RULE 0 phosphonic_ester
RULE 0 phosphoric_acid
RULE 0 phosphoric_ester
RULE 0 sulfonic_acid
RULE 0 sulfonic_ester
RULE 0 tricarbo_phosphene
RULE 0 epoxide
RULE 0 sulfonyl_halide
RULE 0 halopyrimidine
RULE 0 perhalo_ketone
RULE 0 aziridine
RULE 1 oxalyl
RULE 0 alphahalo_amine
RULE 0 halo_amine
RULE 0 halo_alkene
RULE 0 acyclic_NCN
RULE 0 acyclic_NS
RULE 0 SCN2
RULE 0 terminal_vinyl
RULE 0 hetero_hetero
RULE 0 hydrazine
RULE 0 N_methoyl
RULE 0 NS_beta_halothyl
RULE 0 propiolactones
RULE 0 iodoso
RULE 0 iodoxy
RULE 0 noxide
#groups of molecules
RULE 0 dye

#functional groups which are allowed, but may not be wanted in high quantities
#common functional groups
RULE 6 alcohol
RULE 8 alkene
RULE 4 amide
RULE 0 amino_acid
RULE 1 amine
RULE 1 primary_amine
RULE 1 secondary_amine
RULE 4 tertiary_amine
RULE 1 carboxylic_acid
RULE 6 halide
RULE 1 iodine
RULE 4 ketone
RULE 4 phenol
RULE 2 imine
RULE 1 methyl_ketone
RULE 1 alkylaniline
RULE 4 sulfonamide
RULE 1 sulfonylurea
RULE 0 phosphonamide
RULE 0 alphahalo_ketone
RULE 0 oxaziridine
RULE 1 cyclopropyl
RULE 2 guanidine
RULE 0 sulfonimine
RULE 0 sulfinimine
RULE 1 hydroxamic_acid
RULE 0 sulfinylthio
RULE 0 disulfide
RULE 0 enol_ether
RULE 0 enamine
RULE 0 organometallic
RULE 0 dithioacetal
RULE 1 oxime
RULE 0 isothiocyanate
RULE 0 isocyanate
RULE 3 lactone
RULE 3 lactam
RULE 1 thioester
RULE 1 carbonate
RULE 0 carbamic_acid
RULE 1 thiocarbamate
RULE 0 triazine
RULE 1 malonic
#other functional groups
RULE 2 alkyne
RULE 4 aniline
RULE 4 aryl_halide
RULE 4 carbamate
RULE 4 ester
RULE 4 ether
RULE 1 hydrazone
RULE 0 nonacylhydrazone
RULE 1 hydroxylamine
RULE 2 nitrile
RULE 2 sulfide
RULE 2 sulfone
RULE 2 sulfoxide
RULE 0 thiourea
RULE 1 thioamide
RULE 1 thiol
RULE 2 urea
RULE 0 hemiketal
RULE 0 hemiacetal
RULE 0 ketal
RULE 1 acetal
RULE 0 aminal
RULE 0 hemiaminal
#protecting groups
RULE 0 benzyloxycarbonyl_CBZ
RULE 0 t_butoxycarbonyl_tBOC
RULE 0 fluorenylmethoxycarbonyl_Fmoc
RULE 1 dioxolane_5MR
RULE 1 dioxane_6MR
RULE 1 tetrahydropyran_THP
RULE 1 methoxyethoxymethyl_MEM
RULE 2 benzyl_ether
RULE 2 t_butyl_ether
RULE 0 trimethylsilyl_TMS
RULE 0 t_butyldimethylsilyl_TBDMS
RULE 0 triisopropylsilyl_TIPS
RULE 0 t_butyldiphenylsilyl_TBDPS
RULE 1 phthalimides_PHT
RULE 2 arenesulfonyl

**Table S5.** SpaceDock hits as potential dopamine D3 receptor antagonists. Compounds purchased for experimental validation are highlighted in yellow. The ground truth ligand (eticlopride) is highlighted in green.

| Rank | SpaceDock SMILES | IFP_full[a] | IFP_polar[b] | HYDE[c] | Tc-MCS[d] | REAL SMILES[e] | Tc-ECFP4[f] |
|---|---|---|---|---|---|---|---|
| 1 | O=C(NCC[NH+](C1CC1)C(C)C)c2c3OCCc3cc(c2)CC | 0.778 | 1 | -53.27 | 0.96 | O=C(NCCN(C1CC1)C(C)C)c2c3OCCc3cc(c2)C | 0.42 |
| 2 | O=C(NC1(C[NH+](CC)CC)CCCC1)c2c(NC(=O)C(C)(C)C)cccc2 | 0.778 | 1 | -51.79 | 1.00 | O=C(NC1(CN(CC)CC)CCCC1)c2c(NC(=O)C(C)(C)C)cccc2 | 0.41 |
| 3 | S(c1cc(SC)cc(c1)C(=O)NC[C@H]2C[NH+](C(C)C)CCC2)C | 0.813 | 1 | -51.13 | 0.91 | S(c1cc(ccc1)C(=O)NCC2CN(C(C)C)CCC2)C | 0.46 |
| 4 | O=C(NCC[NH+](C1CC1)C(C)C)c2c3c(ccc2)CC(C3)(C)C | 0.765 | 1 | -50.64 | 0.92 | O=C(NCCN(C1CC1)C(C)C)c2c3OC(Cc3ccc2)(C)C | 0.48 |
| 5 | O=C(NC[C@@H]([NH+]1CCCC1)c2ccc(cc2)C)C3=NN(C(=C3)C4CC4)CC | 0.750 | 1 | -49.22 | 0.96 | O=C(NCC(N1CCCC1)c2ccc(cc2)C)C3=NN(C(=C3)C4CC4)C | 0.48 |
| 6 | O=C(NC1(C[NH+](CC)CC)CCCC1)c2c3OCCc3cc(c2)CC | 0.778 | 1 | -49.09 | 0.96 | O=C(NC1(CN(CC)CC)CCCC1)c2c3OCCc3cc(c2)C | 0.39 |
| 7 | O=C(NC[C@H]1[NH+](CCC1)CCC)c2c3c(ccc2)CC(C3)(C)C | 0.867 | 1 | -48.62 | 0.92 | O=C(NCC1N(CCC1)CCC)c2c3OC(Cc3ccc2)(C)C | 0.57 |
| 8 | O=C(NCC[NH+](CCC)CC)c1cc(ccc1)C(C)(C)C | 0.750 | 1 | -48.32 | 1.00 | O=C(NCCN(CCC)CC)c1cc(ccc1)C(C)(C)C | 0.45 |
| 9 | O=C(NCC[NH+](C1CC1)C(C)C)c2c(NC(=O)C(C)(C)C)cccc2 | 0.875 | 1 | -48.31 | 1.00 | O=C(NCCN(C1CC1)C(C)C)c2c(NC(=O)C(C)(C)C)cccc2 | 0.46 |
| 10 | O=C(OCC[NH+](CC)CC)c1cc(NC(=O)CC2CC2)ccc1 | 0.765 | 1 | -48.11 | 1.00 | O=C(OCCN(CC)CC)c1cc(NC(=O)CC2CC2)ccc1 | 0.45 |
| 11 | O=C(NCC[NH+](C1CC1)C(C)C)C=2C(=NN(C2)C)C3CCOCC3 | 0.750 | 1 | -47.82 | 0.96 | O=C(NCCN(C1CC1)C(C)C)C=2C(=NNC2)C3CCOCC3 | 0.43 |
| 12 | O=C(NC1(C[NH+](CC)CC)CCCC1)c2c3c(cc4c2CCC4)CCC3 | 0.765 | 1 | -47.51 | 0.88 | O=C(NC1(CN(CC)CC)CCCC1)c2c3c(ccc2)CCC3 | 0.41 |
| 13 | O=C(NCC[NH+](CCC)CC)c1c(cccc1)C2CCC2 | 0.813 | 1 | -46.91 | 0.87 | O=C(N(CCC)CC)C(=O)NCc1c(cccc1)C2CCC2 | 0.48 |
| 14 | O=C(NC[C@H]1[C@@H]([NH+]2CCCC2)CCCC1)C=3C(=NN(C3C)CC)C | 0.778 | 1 | -46.66 | 1.00 | O=C(NCC1C(N2CCCC2)CCCC1)C=3C(=NN(C3C)CC)C | 0.40 |
| 15 | O=C(NCc1c(c2cnccc2)cccc1)C[C@H]3[NH+](CCC3)CC | 0.813 | 1 | -46.58 | 0.96 | O=C(N1C(CC(=O)NCc2c(c3cnccc3)cccc2)CCC1)C | 0.47 |
| 16 | O=C(NCC1([NH+](C)C)CCCC1)c2c(O)ccc(c2)Cc3ccccc3 | 0.800 | 1 | -46.55 | 1.00 | O=C(NCC1(N(C)C)CCCC1)c2c(O)ccc(c2)Cc3ccccc3 | 0.49 |
| 17 | O=C(NCCC=1c2c(NC1C)cccc2C)C[C@H]3[NH+](CCC3)CC | 0.813 | 1 | -46.46 | 0.92 | O=C(N1C(CC(=O)NCCC=2c3c(NC2)cccc3C)CCC1)C | 0.42 |
| 18 | O=C(NCC[NH+](CCC)CC)c1c(cccc1)C2CCCC2 | 0.813 | 1 | -46.40 | 1.00 | O=C(NCCN(CCC)CC)c1c(cccc1)C2CCCC2 | 0.48 |
| 19 | O=C(NC[C@H]1[NH+](CCC1)CCC)C=2C(=NN(C2)C)C3CCC3 | 0.867 | 1 | -45.87 | 0.95 | N=1N(C=C(C1C2CCC2)CNCC3N(CCC3)CCC)C | 0.53 |
| 20 | O=C(NC(C[NH+](CCC)C)(C)C)c1c(N2c3c(N=C2)cccc3)cccc1 | 0.765 | 1 | -45.78 | 0.93 | O=C(NC(CN(CCCC)C)C)c1c(N2c3c(N=C2)cccc3)cccc1 | 0.41 |
| 21 | O=C(NC[C@@H]1C[NH+](CCCC1)C)C2(c3cc(ccc3)C)CCCCC2 | 0.750 | 1 | -45.48 | 0.96 | O=C(NCC1CNCCCC1)C2(c3cc(ccc3)C)CCCCC2 | 0.48 |
| 22 | O=C(NC[C@H]1C[NH+](CC(C)C)CCC1)c2cc3c(c(c2)C)CCC3 | 0.813 | 1 | -45.38 | 0.96 | O=C(NCC1CN(CC(C)C)CCC1)c2cc3c(cc2)CCC3 | 0.45 |
| 23 | O=C(NCC[NH+](CCC)CC)c1c2c(cc(c1)C)CCC2 | 0.800 | 1 | -45.35 | 0.95 | O=C(NCCN(CCC)CC)c1c2c(ccc1)CCC2 | 0.49 |
| 24 | O=C(NC[C@@H]1C[NH+](CCCC1)C)C2(c3cc(ccc3)C)CCC2 | 0.750 | 1 | -45.28 | 0.96 | O=C(NCC1CNCCCC1)C2(c3cc(ccc3)C)CCC2 | 0.48 |
| 25 | O=C(NCC[NH+](CCC)CC)c1c2NC(=C(c2ccc1)C)C | 0.875 | 1 | -44.85 | 1.00 | O=C(NCCN(CCC)CC)c1c2NC(=C(c2ccc1)C)C | 0.53 |
| 26 | O=C(NC(C[NH+](CCC)C)(C)C)c1c(cc(cc1)C)C2CC2 | 0.765 | 1 | -44.67 | 1.00 | O=C(NC(CN(CCC)C)(C)C)c1c(cc(cc1)C)C2CC2 | 0.40 |

| | | | | | | | |
|---|---|---|---|---|---|---|---|
| 27 | O=C(N[C@@H]1C2(COCC1)CCCC2)CC[NH+]3CCN(c4ccccc4)CC3 | 0.750 | 1 | -44.63 | 1.00 | O=C(NC1C2(COCC1)CCCC2)CCN3CCN(c4ccccc4)CC3 | 0.51 |
| 28 | O=C(NCC[NH+]1[C@H](CCC1)C)C2=C(N(C=C2)C)c3ccccc3 | 0.750 | 1 | -44.50 | 1.00 | O=C(NCCN1C(CCC1)C)C2=C(N(C=C2)C)c3ccccc3 | 0.47 |
| 29 | S1C2=C(C(C(=O)NCC[NH+](CCC)CC)=C1)CC[C@H](C2)C | 0.867 | 1 | -44.37 | 1.00 | S1C2=C(C(C(=O)NCCN(CCC)CC)=C1)CCC(C2)C | 0.40 |
| 30 | Clc1c(OC)c(c(O)c(c1)CC)C(=O)NC[C@H]2[NH+](CCC2)CC | 0.929 | 1 | -44.34 | 0.91 | O=C(NCC1N(CCC1)CC)c2c(OC)ccc(c2)CC | 1.00 |
| 31 | O=C(NC[C@H]1[C@@H]([NH+]2CCCC2)CCCC1)C3(c4cc(C#N)ccc4)CC3 | 0.813 | 1 | -44.16 | 1.00 | O=C(NCC1C(N2CCCC2)CCCC1)C3(c4cc(C#N)ccc4)CC3 | 0.52 |
| 32 | O=C(NC(C[NH+](CCC)C)(C)C)/C(/c1ccccc1)=C/c2ccccc2 | 0.824 | 1 | -44.08 | 0.93 | O=C(NC(CN(CCCC)C)C)C(c1ccccc1)=Cc2ccccc2 | 0.40 |
| 33 | O=C(NCC[NH+](CCC)CC)[C@@H]1CC=2N(N=CC2CC1)C | 0.800 | 1 | -44.05 | 1.00 | O=C(NCCN(CCC)CC)C1CC=2N(N=CC2CC1)C | 0.47 |
| 34 | O=C(NCC[NH+](C(C)C)CCC)C=1N(C=CC1)C(C)C | 0.786 | 1 | -44.04 | 1.00 | O=C(NCCN(C(C)C)CCC)C=1N(C=CC1)C(C)C | 0.47 |
| 35 | O=C(NC[C@H]1C[NH+](CCC1)CC)C=2c3c(OC2)ccc(c3)CC | 0.765 | 1 | -43.92 | 1.00 | O=C(NCC1CN(CCC1)CC)C=2c3c(OC2)ccc(c3)CC | 0.48 |
| 36 | O=C(NCC[NH+](C1CC1)C(C)C)C=2C(=NN(C2)C)[C@H]3COCC3 | 0.813 | 1 | -43.69 | 1.00 | O=C(NCCN(C1CC1)C(C)C)C=2C(=NN(C2)C)C3COCC3 | 0.43 |
| 37 | O=C(NC[C@H]1[NH+](CCC1)CCC)C=2C(=NN(C2)C)COC | 0.867 | 1 | -43.52 | 1.00 | O=C(NCC1N(CCC1)CCC)C=2C(=NN(C2)C)COC | 0.54 |
| 38 | O=C(NC(C[NH+](CCC)C)(C)C)[C@H](c1ccccc1)C(C)C | 0.750 | 1 | -43.48 | 0.96 | O=C(NC(CN(CCC)C)(C)C)C(c1ccc(cc1)C)C(C)C | 0.40 |
| 39 | O=C(NCCC=1c2c(NC1)cccc2)C[C@H]3[NH+](CCC3)CC | 0.750 | 1 | -43.46 | 0.96 | O=C(N1C(CC(=O)NCCC=2c3c(NC2)cccc3)CCC1)C | 0.51 |
| 40 | O=C(NCc1c(nccc1)N2CCCCC2)C[C@H]3[NH+](CCC3)CC | 0.813 | 1 | -43.32 | 0.96 | O=C(N1C(CC(=O)NCc2c(nccc2)N3CCCCC3)CCC1)C | 0.41 |
| 41 | O=C(NC[C@H]1[NH+](CCC1)CCC)C=2N(N=C(C2)C)C3CCC3 | 0.813 | 1 | -42.86 | 1.00 | O=C(NCC1N(CCC1)CCC)C=2N(N=C(C2)C)C3CCC3 | 0.54 |
| 42 | O=C(NC1(C[NH+](CC)CC)CCCC1)C=2C(=NN(C2)C)C3CCOCC3 | 0.765 | 1 | -42.81 | 0.96 | O=C(NC1(CN(CC)CC)CCCC1)C=2C(=NNC2)C3CCOCC3 | 0.43 |
| 43 | O=C(NCC[NH+]1[C@H](CCC1)C)c2c(cccc2)Cc3ccccc3 | 0.813 | 1 | -42.52 | 1.00 | O=C(NCCN1C(CCC1)C)c2c(cccc2)Cc3ccccc3 | 0.48 |
| 44 | O=C(NCC[NH+](C(C)C)CCC)C=1N=C(N2C1CCCC2)C | 0.867 | 1 | -42.44 | 0.92 | O=C(NCCN(C(C)C)CCC)C=1N=C(N2C1CCCC2)C(C)C | 0.43 |
| 45 | O=C(NCC1([NH+](C)C)CCCCC1)c2cc(ccc2)[C@H]3C4(CC3)CCC4 | 0.765 | 1 | -42.36 | 0.89 | O=C(NCC1(N(C)C)CCCCC1)c2cc(ccc2)C3CNCCC3 | 0.46 |
| 46 | O=C(NC(C[NH+](CCC)C)(C)C)[C@H](c1ccccc1)C2CC2 | 0.765 | 1 | -42.01 | 0.92 | Fc1ccc(cc1)C(C(=O)NC(CN(C(=O)CC)C)(C)C)C2CC2 | 0.41 |
| 47 | O=C(NCC[NH+](CCC)CC)c1c2c(ccc1)CCCC2 | 0.867 | 1 | -41.94 | 1.00 | O=C(NCCN(CCC)CC)c1c2c(ccc1)CCCC2 | 0.49 |
| 48 | O=C(NC(C[NH+](CC)CC)(CC)CC)c1c2c(cc(c1)C)CCC2 | 0.765 | 1 | -41.91 | 0.96 | O=C(NC(CN(CC)CC)(CC)CC)c1c2c(ccc1)CCC2 | 0.40 |
| 49 | O=C(NC[C@H]1[NH+](CCC1)CCC)C=2N(N=CC2)C3CCCC3 | 0.750 | 1 | -41.77 | 1.00 | O=C(NCC1N(CCC1)CCC)C=2N(N=CC2)C3CCCC3 | 0.52 |
| 50 | O=C(NC[C@H]1[NH+](C2CC2)CCC1)c3c(cccc3)C4CCCC4 | 0.750 | 1 | -41.65 | 0.88 | O=C(NCC1N(C2CC2)CCCC1)c3c(cccc3)C4CCCC4 | 0.48 |
| 51 | Ic1cc(C#C)cc(c1)C(=O)NCC[NH+](CCC)CCC | 0.813 | 1 | -41.61 | 1.00 | Ic1cc(C#C)cc(c1)C(=O)NCCN(CCC)CCC | 0.50 |
| 52 | O=C(NC(C[NH+](CCC)C)(C)C)C=1N(N=C(C1)C)C2CCOCC2 | 0.765 | 1 | -41.52 | 1.00 | O=C(NC(CN(CCC)C)(C)C)C=1N(N=C(C1)C)C2CCOCC2 | 0.39 |
| 53 | O=C(NCC[NH+](CCC)CC)c1c2c(nccc2)ccc1 | 0.786 | 1 | -41.39 | 1.00 | O=C(NCCN(CCC)CC)c1c2c(nccc2)ccc1 | 0.45 |
| 54 | O=C(N[C@H]1C[NH+](C2CCCC2)CCC1)C=3N4C(=NC3C(C)(C)C)CCCC4 | 0.765 | 1 | -41.33 | 0.89 | O=C(NC1CN(C2CCCC2)CCC1)C=3N=C4N(C3C(C)C)CCCC4 | 0.42 |
| 55 | O=C(NCC1(c2ncccc2)CCC1)[C@H]3C[NH+](CCC3)CC | 0.929 | 1 | -41.32 | 1.00 | O=C(NCC1(c2ncccc2)CCC1)C3CN(CCC3)CC | 0.48 |
| 56 | O=C(NC1(C[NH+](CC)CC)CCCC1)c2cc(ncc2)C3CCC3 | 0.765 | 1 | -41.32 | 0.92 | O=C(NC1(CN(CC)CC)CCCC1)c2cc(ccc2)C3CCC3 | 0.46 |

| | | | | | | | |
|---|---|---|---|---|---|---|---|
| 57 | O=C(NCC[NH+](CCC)CCC)c1c2c(ccc1)C(=O)CCC2 | 0.750 | 1 | -41.09 | 0.96 | O=C(NCCN(CCC)CCC)c1c2c(ccc1)CCCC2 | 0.50 |
| 58 | O=C(NCC[NH+]1[C@H](CCC1)C)C2=C(OC=C2c3ccccc3)C | 0.867 | 1 | -40.94 | 1.00 | O=C(NCCN1C(CCC1)C)C2=C(OC=C2c3ccccc3)C | 0.47 |
| 59 | O=C(NCC[NH+](C1CC1)C(C)C)C2=C(ON=C2)CC(C)C | 0.750 | 1 | -40.80 | 1.00 | O=C(NCCN(C1CC1)C(C)C)C2=C(ON=C2)CC(C)C | 0.39 |
| 60 | O=C(NCC[NH+](C1CC1)C(C)C)c2ncccc2N(C)C | 0.750 | 1 | -40.80 | 1.00 | O=C(NCCN(C1CC1)C(C)C)c2ncccc2N(C)C | 0.43 |
| 61 | O=C(NC[C@H]1[NH+](CCC1)CCC)C=2C(=CNC2)C3CC3 | 0.786 | 1 | -40.74 | 1.00 | O=C(NCC1N(CCC1)CCC)C=2C(=CNC2)C3CC3 | 0.60 |
| 62 | Fc1cc(ccc1)CC2(C(=O)NC[C@H]3[NH+](CCC3)CC)CC2 | 0.867 | 1 | -40.64 | 1.00 | Fc1cc(ccc1)CC2(C(=O)NCC3N(CCC3)CC)CC2 | 0.58 |
| 63 | O=C(NCC1=C2N(N=C1)CC[NH+](C2)C)C=3N(N=C4C3CCCC4)C(C)(C)C | 0.750 | 1 | -40.63 | 0.89 | O=C1N(CCN2N=CC(=C21)CNC(=O)C=3N(N=C4C3CCCC4)CC)C | 0.39 |
| 64 | O=C(NC[C@H]1C[NH+](CC1)CC)C(c2ccccc2)c3ccccc3 | 0.800 | 1 | -40.55 | 1.00 | O=C(NCC1CN(CC1)CC)C(c2ccccc2)c3ccccc3 | 0.55 |
| 65 | O=C(NCC[NH+](C1CC1)C(C)C)c2c(cccc2)[C@@H]3COCC3 | 0.765 | 1 | -40.51 | 1.00 | O=C(NCCN(C1CC1)C(C)C)c2c(cccc2)C3COCC3 | 0.44 |
| 66 | O=C(NC[C@H]1[NH+](CCC1)CCC)C=2C(c3ccccc3)=CNC2 | 0.786 | 1 | -40.38 | 1.00 | O=C(NCC1N(CCC1)CCC)C=2C(c3ccccc3)=CNC2 | 0.57 |
| 67 | O=C(NCC[NH+](CCC)CC)[C@H]1c2c(cccc2)CCC1 | 0.800 | 1 | -40.37 | 1.00 | O=C(NCCN(CCC)CC)C1c2c(cccc2)CCC1 | 0.49 |
| 68 | O=C(NCC[NH+](C1CC1)C(C)C)c2c(NC(=O)CC)cccc2 | 0.813 | 1 | -40.32 | 1.00 | O=C(NCCN(C1CC1)C(C)C)c2c(NC(=O)CC)cccc2 | 0.55 |
| 69 | O=C(NCC[NH+](CCC)CC)c1nc(cc(c1)C)C | 1.000 | 1 | -40.22 | 1.00 | O=C(NCCN(CCC)CC)c1nc(cc(c1)C)C | 0.41 |
| 70 | O=C(NC(C[NH+](CCC)C)(C)C)C=1C(=NN(C1)C)C2CCOCC2 | 0.765 | 1 | -40.21 | 0.88 | O=C(NC(CN(C(=O)CC)C)C)C=1C(=NNC1)C2CCOCC2 | 0.43 |
| 71 | O=C(NC[C@H]1C[NH+](C(C)C)CCC1)c2c(O)ccc(c2)C(C)C | 0.765 | 1 | -40.18 | 1.00 | O=C(NCC1CN(C(C)C)CCC1)c2c(O)ccc(c2)C(C)C | 0.49 |
| 72 | O=C(NC(C[NH+](CCC)C)(C)C)C1=C(OC=C1c2ccccc2)C | 0.750 | 1 | -40.15 | 0.92 | O=C(NC(CN(C(=O)CC)C)C)C1=C(OC=C1c2ccccc2)C | 0.42 |
| 73 | O=C(NC[C@H]1[NH+](CCC1)CCC)c2c(cccc2)C3CC3 | 0.857 | 1 | -40.14 | 1.00 | O=C(NCC1N(CCC1)CCC)c2c(cccc2)C3CC3 | 0.57 |
| 74 | O=C(NCC[NH+](CCC)CC)c1c2c(ccc1)cccc2 | 0.800 | 1 | -40.12 | 1.00 | O=C(NCCN(CCC)CC)c1c2c(ccc1)cccc2 | 0.51 |
| 75 | Brc1cc2c(nccc2)c(c1)C(=O)NC[C@@H]([NH+]3CCCC3)c4ccc(OC)cc4 | 0.824 | 1 | -40.06 | 1.00 | Brc1cc2c(nccc2)c(c1)C(=O)NCC(N3CCCC3)c4ccc(OC)cc4 | 0.49 |
| 76 | O=C(NC(C[NH+](CCC)C)(C)C)c1c2c(nc(c1C)C3CC3)cccc2 | 0.765 | 1 | -40.05 | 0.96 | O=C(NC(CN(CCC)C)(C)C)c1c2c(nc(c1)C3CC3)cccc2 | 0.43 |
| 77 | O=C(NC(C[NH+](CC)CC)(CC)CC)c1c2c(ccc1)[C@H]3O[C@@H]2CC3 | 0.765 | 1 | -39.92 | 0.96 | O=C(NC(CN(CC)CC)(CC)CC)c1c2c(ccc1)CCCC2 | 0.46 |
| 78 | Brc1cc(cc(c1)C(C)C)C(=O)NC[C@@H]([NH+]2CCCC2)c3ccc(cc3)C | 0.813 | 1 | -39.88 | 1.00 | Brc1cc(cc(c1)C(C)C)C(=O)NCC(N2CCCC2)c3ccc(cc3)C | 0.47 |
| 79 | O=C(NC[C@H]1C[NH+](C2CCCC2)CCC1)C=3N(N=C(C3)C(C)C)C | 0.813 | 1 | -39.83 | 1.00 | O=C(NCC1CN(C2CCCC2)CCC1)C=3N(N=C(C3)C(C)C)C | 0.43 |
| 80 | Clc1nc(cc(c1)C(=O)NCC[NH+](CCC)CC)C(C)(C)C | 0.750 | 1 | -39.81 | 0.95 | O=C(NCCN(CCC)CC)c1cc(ncc1)C(C)(C)C | 0.46 |
| 81 | O=C(NC[C@H]1C[NH+](C2CCCC2)CCC1)C=3C(=NN(C3)C(C)C)C | 0.813 | 1 | -39.79 | 1.00 | O=C(NCC1CN(C2CCCC2)CCC1)C=3C(=NN(C3)C(C)C)C | 0.44 |
| 82 | O=C(NC[C@H]1C[NH+](CC(C)C)CCC1)c2c(cc(cc2)C)C3CC3 | 0.824 | 1 | -39.61 | 1.00 | O=C(NCC1CN(CC(C)C)CCC1)c2c(cc(cc2)C)C3CC3 | 0.49 |
| 83 | O=C(NCCC=1c2c(NC1)cccc2C)C[C@H]3[NH+](CCC3)CC | 0.800 | 1 | -39.61 | 0.96 | O=C(N1C(CC(=O)NCCC=2c3c(NC2)cccc3C)CCC1)C | 0.46 |
| 84 | Clc1c(OC)c(cc(Cl)c1)C(=O)NCC[NH+](CCC)CC | 0.933 | 1 | -39.58 | 1.00 | Clc1c(OC)c(cc(Cl)c1)C(=O)NCCN(CCC)CC | 0.59 |
| 85 | O=C(NCC(c1ncccc1)(C)C)[C@H]2C[NH+](CCC2)CC | 0.750 | 1 | -39.55 | 0.95 | Fc1c(nccc1)C(CNC(=O)C2CN(CCC2)CC)(C)C | 0.45 |
| 86 | O=C(NC(C[NH+](CCC)C)(C)C)c1c2c(nc(c1)C)CCCC2 | 0.765 | 1 | -39.54 | 0.96 | O=C(NC(CN(CCC)C)(C)C)c1c2c(nc(c1)C)CCCC2=O | 0.41 |

| | | | | | | | |
|---|---|---|---|---|---|---|---|
| 87 | S1C(=C(C(C(=O)NCC[NH+](CCC)CC)=C1)C2CC2)C | 0.750 | 1 | -39.51 | 0.86 | O=C(NCCN(CCC)CC)C=1C(=CNC1)C2CC2 | 0.50 |
| 88 | BrC1=NN(C(C(=O)NC[C@H]2[NH+](CCC2)CCC)=C1)CC3CC3 | 0.813 | 1 | -39.49 | 0.95 | O=C(NCC1N(CCC1)CCC)C=2N(N=CC2)CC3CC3 | 0.50 |
| 89 | O=C(NC[C@H]([NH+]1CCCC1)c2ccc(cc2)C)c3c(c(c(c3C)C)C)C | 0.778 | 1 | -39.36 | 1.00 | O=C(NCC(N1CCCC1)c2ccc(cc2)C)c3c(c(c(c3C)C)C)C | 0.42 |
| 90 | O=C(NCC[NH+](C1CC1)C(C)C)c2c3c(N=C(C3(C)C)C)ccc2 | 0.824 | 1 | -39.26 | 1.00 | O=C(NCCN(C1CC1)C(C)C)c2c3c(N=C(C3(C)C)C)ccc2 | 0.42 |
| 91 | O=C(NCC1([NH+](C)C)CCCCC1)C2=NN(C3=C2CC(CC3)(C)C)C | 0.813 | 1 | -39.20 | 1.00 | O=C(NCC1(N(C)C)CCCCC1)C2=NN(C3=C2CC(CC3)(C)C)C | 0.39 |
| 92 | O=C(NCC1(c2ncccc2)CC1)[C@H]3C[NH+](CCC3)CC | 0.929 | 1 | -38.83 | 1.00 | O=C(NCC1(c2ncccc2)CC1)C3CN(CCC3)CC | 0.51 |
| 93 | O=C(NC[C@@H]([NH+]1CCCC1)C)[C@@H](c2c(cccc2)C)c3ccccc3 | 0.778 | 1 | -38.79 | 1.00 | O=C(NCC(N1CCCC1)C)C(c2c(cccc2)C)c3ccccc3 | 0.39 |
| 94 | O=C(NC[C@H]1[C@@H]([NH+]2CCCC2)CCCC1)C=3N(N=C(C3)C)CCC | 0.824 | 1 | -38.78 | 1.00 | O=C(NCC1C(N2CCCC2)CCCC1)C=3N(N=C(C3)C)CCC | 0.39 |
| 95 | O=C(NCC[NH+](CCC)CC)C1=NN(C2=C1CCCC2)C | 0.867 | 1 | -38.76 | 1.00 | O=C(NCCN(CCC)CC)C1=NN(C2=C1CCCC2)C | 0.43 |
| 96 | O=C(NCC[NH+](C1CC1)C(C)C)C=2C(c3ccccc3)=CNC2 | 0.750 | 1 | -38.71 | 1.00 | O=C(NCCN(C1CC1)C(C)C)C=2C(c3ccccc3)=CNC2 | 0.53 |
| 97 | O=C(NC[C@H]1C[NH+](CCC1)CC)C2=C(OC(=C2)C)CC | 0.800 | 1 | -38.66 | 1.00 | O=C(NCC1CN(CCC1)CC)C2=C(OC(=C2)C)CC | 0.50 |
| 98 | O=C(N[C@H](CC[NH+](C)C)C)CC=1c2c(NC1C)cccc2 | 0.786 | 1 | -38.61 | 1.00 | O=C(NC(CCN(C)C)C)CC=1c2c(NC1C)cccc2 | 0.51 |
| 99 | O=C(NC[C@H]1[NH+](CCC1)CC)C=2N(N=CC2)C3CCCC3 | 0.800 | 1 | -38.57 | 1.00 | O=C(NCC1N(CCC1)CC)C=2N(N=CC2)C3CCCC3 | 0.54 |
| 100 | O=C(NCC[NH+](C(C)C)CCC)C=1N(C=CC1)C2CC2 | 0.786 | 1 | -38.50 | 1.00 | O=C(NCCN(C(C)C)CCC)C=1N(C=CC1)C2CC2 | 0.44 |
| 101 | O=C(NC[C@@H]([NH+]1CCCC1)C)c2c(cccc2)[C@@H]3COCC3 | 0.765 | 1 | -38.48 | 1.00 | O=C(NCC(N1CCCC1)C)c2c(cccc2)C3COCC3 | 0.45 |
| 102 | O=C(NC[C@H]1[C@@H]([NH+]2CCCC2)CCCC1)c3c4c(ccc3)CC(C4)(C)C | 0.765 | 1 | -38.45 | 0.93 | O=C(NCC1C(N2CCCC2)CCCC1)c3c4OC(Cc4ccc3)(C)C | 0.42 |
| 103 | O=C(NC[C@H]([NH+]1CCCC1)C)C=2C(=NN(C2)C(C)C)C3CC3 | 0.750 | 1 | -38.20 | 0.95 | O=C(NCC(N1CCCC1)C)C=2C(=NN(C2)CC)C3CC3 | 0.40 |
| 104 | O=C(NC[C@H]1[NH+](CCC1)CCC)C2(N3c4c(N=C3)cccc4)CC2 | 0.813 | 1 | -38.17 | 0.76 | O=C(NCC1N(CCC1)CCC)C2(N3N=CN=C3)CC2 | 0.51 |
| 105 | O=C(NCC(c1ncccc1)(C)C)[C@H]2C[NH+](CCC2)CCC | 0.929 | 1 | -38.08 | 0.96 | Fc1c(nccc1)C(CNC(=O)C2CN(CCC2)CCC)(C)C | 0.51 |
| 106 | O=C(NCC[NH+]1[C@H](CCC1)C)c2c(cccc2)COC(C)(C)C | 0.765 | 1 | -37.99 | 1.00 | O=C(NCCN1C(CCC1)C)c2c(cccc2)COC(C)(C)C | 0.50 |
| 107 | S1c2c(c(ccc2)C(=O)NCC[NH+](C(C)C)CCC)C=C1 | 0.857 | 1 | -37.91 | 1.00 | S1c2c(c(ccc2)C(=O)NCCN(C(C)C)CCC)C=C1 | 0.44 |
| 108 | Brc1c(OC)c(ccc1)C(=O)NCC[NH+](C(C)C)CCC | 0.750 | 1 | -37.78 | 1.00 | Brc1c(OC)c(ccc1)C(=O)NCCN(C(C)C)CCC | 0.50 |
| 109 | Brc1cc(OC)cc(c1)C(=O)NCC[NH+](C2CC2)C(C)C | 0.750 | 1 | -37.77 | 1.00 | Brc1cc(OC)cc(c1)C(=O)NCCN(C2CC2)C(C)C | 0.46 |
| 110 | S1c2c(N=C1C)cccc2C(=O)NC(C[NH+](CC)CC)(CC)CC | 0.778 | 1 | -37.75 | 1.00 | S1c2c(N=C1C)cccc2C(=O)NC(CN(CC)CC)(CC)CC | 0.42 |
| 111 | IC=1N(N=C(C1C(=O)NCCC[NH+](CC)CC)C)C | 0.750 | 1 | -37.66 | 1.00 | IC=1N(N=C(C1C(=O)NCCCN(CC)CC)C)C | 0.43 |
| 112 | O=C(NC(C[NH+](CCC)C)(C)C)[C@@]1(OCCC1)c2ccccc2 | 0.765 | 1 | -37.64 | 0.88 | Brc1c(cccc1)C2(OCCC2)C(=O)NC(CN(C(=O)CC)C)C | 0.43 |
| 113 | O=C(NC[C@H]1[NH+](CCC1)CCC)C2=C(OC=C2C)C3CC3 | 0.867 | 1 | -37.60 | 1.00 | O=C(NCC1N(CCC1)CCC)C2=C(OC=C2C)C3CC3 | 0.50 |
| 114 | O=C(NCC[NH+](C(C)C)CCC)c1ncccc1N(C)C | 0.857 | 1 | -37.50 | 1.00 | O=C(NCCN(C(C)C)CCC)c1ncccc1N(C)C | 0.41 |
| 115 | BrC1=CN(C(C(=O)NCC[NH+](C2CC2)C(C)C)=C1)CC | 0.765 | 1 | -37.50 | 1.00 | BrC1=CN(C(C(=O)NCCN(C2CC2)C(C)C)=C1)CC | 0.45 |
| 116 | O=C(NC1(C[NH+](CC)CC)CCCC1)c2cc(ncc2)C3CC3 | 0.750 | 1 | -37.43 | 1.00 | O=C(NC1(CN(CC)CC)CCCC1)c2cc(ncc2)C3CC3 | 0.47 |

| | | | | | | | |
|---|---|---|---|---|---|---|---|
| 117 | O=C(NC[C@H]1C[NH+](CCC1)CC)C2=C(N(C=C2)CC)C | 0.800 | 1 | -37.40 | 0.95 | O=C(NCC1CN(CCC1)CC)C2=C(N(C(=C2)C)CC)C | 0.52 |
| 118 | O=C(NCC[NH+](CCC)CC)c1c2c(ccc1)COC2 | 0.857 | 1 | -37.39 | 0.90 | O=C(NCCN(CCC)CC)c1c2c(ccc1)CCC2 | 0.50 |
| 119 | O=C(N[C@H]1C[NH+](C(C)(C)C)CCC1)c2c(cccc2)C3CCCC3 | 0.765 | 1 | -37.34 | 1.00 | O=C(NC1CN(C(C)(C)C)CCC1)c2c(cccc2)C3CCCC3 | 0.45 |
| 120 | O=C(N[C@@H](C[NH+](CCC)CC)C)c1ncc(cc1OC)C | 0.750 | 1 | -37.17 | 0.95 | O=C(NC(CN(C(=O)CC)CC)C)c1ncc(cc1OC)C | 0.44 |
| 121 | Clc1cc(c(OC)cc1)C(=O)NCC[NH+](C2CC2)C(C)C | 0.750 | 1 | -37.11 | 1.00 | Clc1cc(c(OC)cc1)C(=O)NCCN(C2CC2)C(C)C | 0.53 |
| 122 | Clc1nc(Cl)cc(c1C)C(=O)NCC[NH+](C2CC2)C(C)C | 0.824 | 1 | -36.99 | 0.86 | ClC=1NC(=O)C=C(C(=O)NCCN(C2CC2)C(C)C)C1 | 0.38 |
| 123 | O=C(NCC[NH+](C(C)C)CCC)C1=NC=CN1C(C)C | 0.786 | 1 | -36.84 | 1.00 | O=C(NCCN(C(C)C)CCC)C1=NC=CN1C(C)C | 0.42 |
| 124 | O=C(NCC[NH+](CCC)CC)C1=NN(C2=C1CCC2)C | 0.765 | 1 | -36.84 | 1.00 | O=C(NCCN(CCC)CC)C1=NN(C2=C1CCC2)C | 0.43 |
| 125 | Clc1c(Cl)cc(Cl)c(O)c1C(=O)NC[C@H]2[NH+](CCC2)CCC | 0.867 | 1 | -36.75 | 1.00 | Clc1c(Cl)cc(Cl)c(O)c1C(=O)NCC2N(CCC2)CCC | 0.73 |
| 126 | O=C(NC[C@H]1[C@@H]([NH+]2CCCC2)CCCC1)C=3N(N=C4C3CCCC4)C | 0.765 | 1 | -36.69 | 1.00 | O=C(NCC1C(N2CCCC2)CCCC1)C=3N(N=C4C3CCCC4)C | 0.39 |
| 127 | O=C(NC(C[NH+](CC)CC)(CC)CC)c1c2c(ncc1)cc(cc2)C | 0.765 | 1 | -36.67 | 0.96 | Clc1c2nccc(c2ccc1C)C(=O)NC(CN(CC)CC)(CC)CC | 0.39 |
| 128 | O=C(NC[C@H]1C[NH+](CC(C)C)CCC1)c2nc(cc(c2)C)C | 0.857 | 1 | -36.66 | 1.00 | O=C(NCC1CN(CC(C)C)CCC1)c2nc(cc(c2)C)C | 0.46 |
| 129 | Brc1cc(cc(c1)C(C)C)C(=O)NCC[NH+]2[C@H](CCC2)C | 0.750 | 1 | -36.62 | 0.95 | Brc1cc(cc(c1)C(C)C)C(=O)NCCN2CCCC2 | 0.43 |
| 130 | O=C(NC(C[NH+](CCC)C)(C)C)c1cc(Oc2ccncc2)ccc1 | 0.875 | 1 | -36.55 | 1.00 | O=C(NC(CN(CCC)C)(C)C)c1cc(Oc2ccncc2)ccc1 | 0.41 |
| 131 | O=C(NCC1(c2ncccc2)CCC1)[C@H]3C[NH+](CCC3)CCC | 0.929 | 1 | -36.43 | 1.00 | O=C(NCC1(c2ncccc2)CCC1)C3CN(CCC3)CCC | 0.49 |
| 132 | O=C(NCC[NH+](C(C)C)CCC)c1c2c(ccc1)COCC2 | 0.867 | 1 | -36.40 | 1.00 | O=C(NCCN(C(C)C)CCC)c1c2c(ccc1)COCC2 | 0.44 |
| 133 | BrC1=CN(C(C(=O)NCC[NH+](CCC)CC)=C1)CC | 0.750 | 1 | -36.36 | 1.00 | BrC1=CN(C(C(=O)NCCN(CCC)CC)=C1)CC | 0.45 |
| 134 | O=C(NCC(c1ncccc1)(CC)CC)[C@H]2C[NH+](C(C)C)CCC2 | 0.765 | 1 | -36.35 | 1.00 | O=C(NCC(c1ncccc1)(CC)CC)C2CN(C(C)C)CCC2 | 0.46 |
| 135 | Clc1cc2c(c(ccc2)C(=O)NC(C[NH+](CC)CC)(CC)CC)cc1 | 0.778 | 1 | -36.26 | 1.00 | Clc1cc2c(c(ccc2)C(=O)NC(CN(CC)CC)(CC)CC)cc1 | 0.45 |
| 136 | Clc1c(cc(cc1)C(=O)NC[C@H]2C[NH+](CCC2)CC)CCC | 0.765 | 1 | -36.24 | 0.95 | Clc1c(cc(cc1)C(=O)NCC2CN(CCC2)CC)CC | 0.53 |
| 137 | O=C(N[C@H](CC[NH+](C)C)C)C1CCN(c2ncccc2)CC1 | 0.800 | 1 | -36.19 | 1.00 | O=C(NC(CCN(C)C)C)C1CCN(c2ncccc2)CC1 | 0.48 |
| 138 | ClC=1SC=C(Cl)C1C(=O)NCCC[NH+](CC)CC | 0.800 | 1 | -36.18 | 0.94 | ClC=1C(C(=O)NCCCN(CC)CC)=CSC1 | 0.48 |
| 139 | O=C(NCC[C@@H]([NH+]1CCC1)C)[C@H](c2ccccc2)C=C | 0.750 | 1 | -36.14 | 0.90 | O=C(NCCCN1CCC1)C(c2ccccc2)C | 0.41 |
| 140 | O=C(NCC[NH+]1[C@H](CCC1)C)c2cc(OC(C)C)ccc2 | 0.867 | 1 | -36.12 | 0.95 | Brc1c(OC(C)C)cc(cc1)C(=O)NCCN2C(CCC2)C | 0.49 |
| 141 | O=C(NCC[NH+]1[C@H](CCC1)C)c2c(c(cc(c2)C)C)C | 0.800 | 1 | -36.11 | 1.00 | O=C(NCCN1C(CCC1)C)c2c(c(cc(c2)C)C)C | 0.46 |
| 142 | Brc1cc2NC=Nc2c(c1)C(=O)NCC[NH+](CCC)CC | 0.800 | 1 | -36.06 | 1.00 | Brc1cc2NC=Nc2c(c1)C(=O)NCCN(CCC)CC | 0.48 |
| 143 | Clc1c(O)c(cc(Cl)c1)C(=O)NCC[NH+](C2CC2)C(C)C | 0.824 | 1 | -36.03 | 1.00 | Clc1c(O)c(cc(Cl)c1)C(=O)NCCN(C2CC2)C(C)C | 0.45 |
| 144 | S1C=C(C(=O)NCC[NH+](CCC)CC)C(=C1)CC | 0.786 | 1 | -36.03 | 1.00 | S1C=C(C(=O)NCCN(CCC)CC)C(=C1)CC | 0.50 |
| 145 | O=C(NCC[C@@H]([NH+]1CCC1)C)C2(c3ccccc3)CC2 | 0.750 | 1 | -35.93 | 0.95 | O=C(NCCCN1CCC1)C2(c3ccccc3)CC2 | 0.48 |
| 146 | O=C(NC[C@H]1[NH+](CCC1)CCC)C2=C(ON=C2C)CC | 0.750 | 1 | -35.87 | 1.00 | O=C(NCC1N(CCC1)CCC)C2=C(ON=C2C)CC | 0.55 |

| | | | | | | | |
|---|---|---|---|---|---|---|---|
| 147 | O=C(NC[C@H]1[C@@H]([NH+]2CCCC2)CCCC1)c3cc(c(OC)cc3)C4CC4 | 0.765 | 1 | -35.82 | 0.92 | O=C(NCC1C(N2CCCC2)CCCC1)c3cc(ccc3)C4CC4 | 0.46 |
| 148 | O=C(NC(C[NH+](CCC)C)(C)C)c1c(cccc1)[C@H]2COCC2 | 0.765 | 1 | -35.73 | 0.92 | O=C(NC(CN(C(=O)CC)C)C)c1c(cccc1)C2COCC2 | 0.42 |
| 149 | Brc1c(cccc1)CCNC(=O)C[C@H]2[NH+](CCC2)CC | 0.800 | 1 | -35.63 | 0.95 | Brc1c(cccc1)CCNC(=O)CC2N(C(=O)C)CCC2 | 0.50 |
| 150 | Clc1c(c2OCOc2cc1)C(=O)NCC[NH+](CCC)CCC | 0.800 | 1 | -35.52 | 1.00 | Clc1c(c2OCOc2cc1)C(=O)NCCN(CCC)CCC | 0.45 |
| 151 | O=C(NC[C@]1([NH+](C)C)C[C@H](CCC1)C)c2c3OCCc3cc(c2)CC | 0.765 | 1 | -35.50 | 0.96 | O=C(NCC1(N(C)C)CC(CCC1)C)c2c3OCCc3cc(c2)C | 0.42 |
| 152 | O=C(NC[C@@H]([NH+]1CCCC1)c2ccc(OC)cc2)c3c4nc(O)ccc4ccc3 | 0.800 | 1 | -35.48 | 0.93 | Brc1c2c(nccc2)c(cc1)C(=O)NCC(N3CCCC3)c4ccc(OC)cc4 | 0.45 |
| 153 | O=C(NC[C@@H]1C[NH+](CCCC1)C)C=2C(=NN(C2)C(C)(C)C)C(C)(C)C | 0.765 | 1 | -35.46 | 0.96 | O=C(NCC1CNCCCC1)C=2C(=NN(C2)C(C)(C)C)C(C)(C)C | 0.40 |
| 154 | O=C(NC[C@H]1[NH+](C2CC2)CCC1)CC3(Oc4c(O3)cccc4)C | 0.800 | 1 | -35.39 | 0.77 | O=C(NCCC1Oc2c(OC1)cccc2)C3N(C4CC4)CCC3 | 0.49 |
| 155 | O=C(NC1(C[NH+](CC)CC)CCCC1)c2c3c(OC(=C3)C)ccc2 | 0.824 | 1 | -35.37 | 1.00 | O=C(NC1(CN(CC)CC)CCCC1)c2c3c(OC(=C3)C)ccc2 | 0.45 |
| 156 | O=C(NCC[NH+](C1CC1)C(C)C)c2c(cncc2)CC | 0.800 | 1 | -35.26 | 1.00 | O=C(NCCN(C1CC1)C(C)C)c2c(cncc2)CC | 0.43 |
| 157 | O=C(NCC[NH+](CCC)CCC)c1c2c(ncc(c2)C)ccc1 | 0.800 | 1 | -35.22 | 1.00 | O=C(NCCN(CCC)CCC)c1c2c(ncc(c2)C)ccc1 | 0.44 |
| 158 | Fc1cc(c2c(c1)CCC2)C(=O)NC[C@H]3[NH+](C4CC4)CCC3 | 0.857 | 1 | -35.16 | 0.91 | Fc1cc(c2c(c1)CCC2)CNC(=O)C3N(C4CC4)CCC3 | 0.51 |
| 159 | S(=O)(=O)(C=1C(C(=O)NCC[NH+](C2CC2)C(C)C)=CSC1)C | 0.857 | 1 | -35.13 | 1.00 | S(=O)(=O)(C=1C(C(=O)NCCN(C2CC2)C(C)C)=CSC1)C | 0.46 |
| 160 | Clc1c(OC)c(cc(Cl)c1)C(=O)N[C@@H](C[NH+](CCC)CC)C | 0.824 | 1 | -35.12 | 0.96 | Clc1c(OC)c(cc(Cl)c1)C(=O)NC(CN(C(=O)CC)CC)C | 0.45 |
| 161 | S1CC=2C(=NN(C2CC1)C)C(=O)NCC[NH+](CCC)CC | 0.800 | 1 | -35.07 | 1.00 | S1CC=2C(=NN(C2CC1)C)C(=O)NCCN(CCC)CC | 0.39 |
| 162 | S1CC=2C(=NN(C2C(=O)NC3(C[NH+](CC)CC)CCCC3)C)CC1 | 0.765 | 1 | -34.99 | 0.92 | S1(=O)(=O)CC2=C(N(N=C2CC1)C)C(=O)NC3(CN(CC)CC)CCCC<br>3 | 0.38 |
| 163 | O=C(NCc1ncc(cc1C)C)[C@H]2C[NH+](CCC2)CCC | 0.786 | 1 | -34.95 | 1.00 | O=C(NCc1ncc(cc1C)C)C2CN(CCC2)CCC | 0.46 |
| 164 | S(=O)(=O)(NC(C)(C)C)c1cc(ccc1)C(=O)NCC2(C[NH+](C)C)CCCC2 | 0.750 | 1 | -34.81 | 1.00 | S(=O)(=O)(NC(C)(C)C)c1cc(ccc1)C(=O)NCC2(CN(C)C)CCCC2 | 0.44 |
| 165 | O=C(NC[C@H]1[C@@H]([NH+]2CCCC2)CCCC1)c3c(NC(=O)C(C)(C)C)cccc3 | 0.778 | 1 | -34.79 | 1.00 | O=C(NCC1C(N2CCCC2)CCCC1)c3c(NC(=O)C(C)(C)C)cccc3 | 0.46 |
| 166 | O=C(N[C@H]1C[NH+](C2CCCCCC2)CC1)C=3N4C(=NC3C(C)(C)C)CCCC4 | 0.778 | 1 | -34.77 | 0.90 | O=C(NC1CN(C2CCCCCC2)CC1)C=3N=C4N(C3C(C)C)CCCC4 | 0.45 |
| 167 | O=C(NC[C@H]1[C@@H]([NH+]2CCCC2)CCCC1)C=3c4c(N(C3)C)cccc4 | 0.765 | 1 | -34.74 | 0.96 | Clc1cc2c(N(C=C2C(=O)NCC3C(N4CCCC4)CCCC3)C)cc1 | 0.45 |
| 168 | O=C(NC1(C[NH+](CC)CC)CCCC1)c2c(c(OC(=O)C)ccc2)C | 0.778 | 1 | -34.73 | 0.92 | O=C(NC1(CN(CC)CC)CCCC1)c2c(c(OC)ccc2)C | 0.42 |
| 169 | O=C(NC[C@H]1C[NH+](CC(C)C)CCC1)C=2N(N=C(C2)CCC)C | 0.750 | 1 | -34.72 | 0.96 | O=C(NCC1CN(CC(C)C)CCC1)C=2N(N=C(C2)CC(C)C)C | 0.46 |
| 170 | O=C(NCC=1C(=NN(C1C)C)C)[C@H]2C[NH+](CC(C)C)CCC2 | 0.929 | 1 | -34.67 | 1.00 | O=C(NCC=1C(=NN(C1C)C)C)C2CN(CC(C)C)CCC2 | 0.41 |
| 171 | O=C(NCC[NH+](CCC)CC)c1c2c(cc(c1)C)ccnc2 | 0.800 | 1 | -34.67 | 1.00 | O=C(NCCN(CCC)CC)c1c2c(cc(c1)C)ccnc2 | 0.42 |
| 172 | O=C(NC[C@H]1C[NH+](CCC1)CC)c2ccc(cc2)CCC | 0.750 | 1 | -34.65 | 1.00 | O=C(NCC1CN(CCC1)CC)c2ccc(cc2)CCC | 0.51 |
| 173 | Clc1nc(N(C)C)cc(c1)C(=O)NCC[NH+](CCC)CC | 0.875 | 1 | -34.61 | 1.00 | Clc1nc(N(C)C)cc(c1)C(=O)NCCN(CCC)CC | 0.45 |
| 174 | Clc1cc2c(c(c1)C(=O)NC[C@H]3[NH+](CCC3)CCC)cccc2 | 0.867 | 1 | -34.60 | 1.00 | Clc1cc2c(c(c1)C(=O)NCC3N(CCC3)CCC)cccc2 | 0.57 |
| 175 | S1c2c(cccc2)C(C(=O)NCC[NH+](CCC)CC)=C1 | 0.800 | 1 | -34.55 | 1.00 | S1c2c(cccc2)C(C(=O)NCCN(CCC)CC)=C1 | 0.49 |

| | | | | | | | |
|---|---|---|---|---|---|---|---|
| 176 | O=C(NCC[NH+](CCC)CC)c1cnc2ON=C(c2c1)C(C)(C)C | 0.750 | 1 | -34.47 | 0.96 | O=C(NCCN(CCC)CC)c1cnc2ON=C(c2c1)C(C)(C)C | 0.39 |
| 177 | O=C(NCC1([NH+](C)C)CCCCCC1)c2c3c(ccc2)CC(C3)(C)C | 0.750 | 1 | -34.46 | 0.92 | O=C(NCC1(N(C)C)CCCCCC1)c2c3OC(Cc3ccc2)(C)C | 0.42 |
| 178 | O=C(NC[C@H]1[C@@H]([NH+]2CCCC2)CCCC1)c3c(cccc3)C4CCCC4 | 0.765 | 1 | -34.46 | 1.00 | O=C(NCC1C(N2CCCC2)CCCC1)c3c(cccc3)C4CCCC4 | 0.42 |
| 179 | O=C(NCC[NH+](CCC)CC)c1c2OC=Cc2cc(c1)C | 0.800 | 1 | -34.45 | 1.00 | O=C(NCCN(CCC)CC)c1c2OC=Cc2cc(c1)C | 0.44 |
| 180 | O=C(NCc1ncc(cc1C)C)[C@H]2C[NH+](CC(C)C)CCC2 | 0.929 | 1 | -34.44 | 1.00 | O=C(NCc1ncc(cc1C)C)C2CN(CC(C)C)CCC2 | 0.45 |
| 181 | ClC1=CN(C(C(=O)NC[C@H]2[NH+](C3CC3)CCC2)=C1)CC | 0.750 | 1 | -34.43 | 0.86 | ClC1=CN(C(C(=O)NCC2N(C3CC3)CCCC2)=C1)CC | 0.46 |
| 182 | O=C(NCc1cc(OCC[NH+](C)C)ccc1)C2CC=CC2 | 0.786 | 1 | -34.43 | 1.00 | O=C(NCc1cc(OCCN(C)C)ccc1)C2CC=CC2 | 0.47 |
| 183 | O=C(NCC[NH+]1[C@H](CCC1)C)c2c(c3c(cccc3)C)cccc2 | 0.765 | 1 | -34.43 | 1.00 | O=C(NCCN1C(CCC1)C)c2c(c3c(cccc3)C)cccc2 | 0.47 |
| 184 | S1C(=NC=C1C)[C@@H](NC(=O)C2(C[NH+](C)C)CCCCC2)C3CC3 | 0.750 | 1 | -34.35 | 0.91 | S1C(=NC=C1C)C(NC(=O)C2(CN)CCCCC2)C3CC3 | 0.38 |
| 185 | O=C(NC(C[NH+](CC)CC)(CC)CC)c1c2c(ncc1C)cccc2 | 0.813 | 1 | -34.32 | 0.96 | O=C(NC(CN(CC)CC)(CC)CC)c1c2c(ncc1)cccc2 | 0.41 |
| 186 | O=C(NC[C@@H]1C[NH+](CCCC1)C)c2c3c(cc4c2CCC4)CCC3 | 0.750 | 1 | -34.30 | 0.83 | O=C(NCC1CNCCCC1)c2c3c(ccc2)CCC3 | 0.46 |
| 187 | O=C(NC[C@@H]([NH+]1CCCC1)C(CC)CC)c2cc(nc(c2)C)C | 0.824 | 1 | -34.28 | 0.91 | n1c(cc(cc1)CNCC(N2CCCC2)C(CC)CC)C | 0.41 |
| 188 | Brc1c2OCCc2cc(c1)C(=O)NC[C@@H]([NH+]3CCCC3)c4ccc(OC)cc4 | 0.765 | 1 | -34.23 | 0.96 | O=C(NCC(N1CCCC1)c2ccc(OC)cc2)c3cc4c(OCC4)cc3 | 0.55 |
| 189 | O=C(NC[C@H]1[NH+](C2CC2)CCC1)C=3N=COC3C4CCCCC4 | 0.800 | 1 | -34.23 | 0.88 | O=C(NCC1N(C2CC2)CCCC1)C=3N=COC3C4CCCCC4 | 0.42 |
| 190 | O=C(NC(C[NH+](CCC)C)(C)C)CC=1N(N=C(C1)CC)C | 0.765 | 1 | -34.17 | 0.95 | O=C(NC(CN(CCC)C)(C)C)CC=1N(N=C(C1)C)C | 0.36 |
| 191 | O=C(NCC[C@@H]([NH+]1CCC1)C)C=2c3c(NC2C)cccc3 | 0.765 | 1 | -34.13 | 0.95 | O=C(NCCCN1CCC1)C=2c3c(NC2C)cccc3 | 0.45 |
| 192 | O=C(N[C@H]1C[NH+](C2CCCC2)CCC1)c3c(c(ccc3C)C)C | 0.750 | 1 | -34.12 | 1.00 | O=C(NC1CN(C2CCCC2)CCC1)c3c(c(ccc3C)C)C | 0.46 |
| 193 | Clc1cc(c(c(c1)C)C)C(=O)NCC[NH+](C2CC2)C(C)C | 0.867 | 1 | -34.11 | 1.00 | Clc1cc(c(c(c1)C)C)C(=O)NCCN(C2CC2)C(C)C | 0.40 |
| 194 | O=C(NC(C[NH+](CCC)C)(C)C)[C@@]1(c2c(OCC1)cccc2)C | 0.765 | 1 | -34.10 | 0.96 | O=C(NC(CN(CCC)C)(C)C)C1c2c(OCC1)cccc2 | 0.45 |
| 195 | O=C(NC[C@H]1[NH+](CCC1)CCC)c2c(ccnc2)C3OCCO3 | 0.786 | 1 | -34.02 | 1.00 | O=C(NCC1N(CCC1)CCC)c2c(ccnc2)C3OCCO3 | 0.53 |
| 196 | S1C=C(C(=O)NC2(C[NH+](CC)CC)CCCC2)C(=C1)C3CC3 | 0.765 | 1 | -33.95 | 0.91 | O=C(NC1(CN(CC)CC)CCCC1)C=2C(=CNC2)C3CC3 | 0.42 |
| 197 | O=C(NCC[NH+](C(C)C)CC)C1=C(OC=C1)C2CC2 | 0.786 | 1 | -33.89 | 1.00 | O=C(NCCN(C(C)C)CC)C1=C(OC=C1)C2CC2 | 0.43 |
| 198 | O=C(NC(C[NH+](CCC)C)(C)C)c1ncccc1N(C)C | 0.750 | 1 | -33.87 | 0.91 | O=C(NC(CN(C(=O)CC)C)C)c1ncccc1N(C)C | 0.42 |
| 199 | Brc1cc(Cl)c(O)c(c1)C(=O)NC[C@@H]([NH+]2CCCC2)c3ccc(cc3)CC | 0.765 | 1 | -33.84 | 0.96 | Brc1cc(Cl)c(O)c(c1)C(=O)NCC(N2CCCC2)c3ccc(cc3)C | 0.52 |
| 200 | O=C(NCc1cncc(c1)C(C)(C)C)C2(C[NH+]3CCCC3)CCCC2 | 0.765 | 1 | -33.82 | 0.81 | O=C(N1CCCC1)C2(C(=O)NCc3cncc(c3)C(C)(C)C)CC2 | 0.41 |
| 201 | O=C(NCC[NH+](C1CC1)C(C)C)C=2C(=NN(C2)C)C(C)(C)C | 0.824 | 1 | -33.66 | 0.96 | O=C(NCCN(C1CC1)C(C)C)C=2C(=NN(C2)CC)C(C)(C)C | 0.41 |
| 202 | Clc1cc(cc(c1)CC)C(=O)NC[C@H]2C[NH+](CCC2)CC | 0.765 | 1 | -33.59 | 1.00 | Clc1cc(cc(c1)CC)C(=O)NCC2CN(CCC2)CC | 0.49 |
| 203 | Fc1cc2c(c(c1)C(=O)NCC[NH+](CCC)CC)cccc2 | 0.800 | 1 | -33.56 | 0.95 | O=C(NCCN(CCC)CC)c1c2c(ccc1)cccc2 | 0.55 |
| 204 | O=C(NC[C@H]1[NH+](CCC1)CCC)c2c3c(ccc2)COCC3 | 0.929 | 1 | -33.49 | 1.00 | O=C(NCC1N(CCC1)CCC)c2c3c(ccc2)COCC3 | 0.57 |
| 205 | O=C(NC[C@H]1[NH+](CCC1)CCC)C2(c3ccccc3)CC(=O)C2 | 0.857 | 1 | -33.47 | 0.96 | Brc1cc(ccc1)C2(C(=O)NCC3N(CCC3)CCC)CC(=O)C2 | 0.55 |

| | | | | | | | |
|---|---|---|---|---|---|---|---|
| 206 | Clc1cc(c(OC)c(c1)C)C(=O)NC(C[NH+](CCC)C)(C)C | 0.765 | 1 | -33.45 | 0.95 | Clc1cc(c(OC)cc1)C(=O)NC(CN(CCC)C)(C)C | 0.37 |
| 207 | O=C(NC[C@H]1[NH+](C2CC2)CCC1)c3c4c(cnc3)CCCC4 | 0.786 | 1 | -33.43 | 0.83 | O=C(NCc1c2c(ccc1)CCCC2)C3N(C4CC4)CCC3 | 0.45 |
| 208 | O=C(NCC[NH+](CCC)CC)c1c2c(OC(=C2)C)ccc1 | 0.867 | 1 | -33.42 | 1.00 | O=C(NCCN(CCC)CC)c1c2c(OC(=C2)C)ccc1 | 0.47 |
| 209 | Fc1cc(c(cc1)C(=O)NC[C@H]2[C@@H]([NH+]3CCCC3)CCCC2)CC | 0.824 | 1 | -33.41 | 1.00 | Fc1cc(c(cc1)C(=O)NCC2C(N3CCCC3)CCCC2)CC | 0.48 |
| 210 | Clc1nc(cc(c1)C(=O)NCC[NH+](CCC)CC)CC | 0.857 | 1 | -33.41 | 0.95 | O=C(NCCN(CCC)CC)c1cc(ncc1)CC | 0.41 |
| 211 | Fc1c(OC)c(c(OC)cc1)C(=O)NC2(C[NH+](CC)CC)CCCC2 | 0.765 | 1 | -33.40 | 0.96 | O=C(NC1(CN(CC)CC)CCCC1)c2c(OC)cccc2OC | 0.43 |
| 212 | O=C(NCC1(N2N=CC=C2)CC1)[C@H]3C[NH+](CC(C)C)CCC3 | 0.786 | 1 | -33.40 | 1.00 | O=C(NCC1(N2N=CC=C2)CC1)C3CN(CC(C)C)CCC3 | 0.44 |
| 213 | O=C(NCC[NH+](C(C)C)CCC)c1c2c(OCC2)ccc1 | 0.800 | 1 | -33.39 | 1.00 | O=C(NCCN(C(C)C)CCC)c1c2c(OCC2)ccc1 | 0.49 |
| 214 | Clc1c2ncccc2c(c(Cl)c1)C(=O)NC[C@H]3[NH+](CCC3)CCC | 0.800 | 1 | -33.35 | 0.92 | O=C(NCC1N(CCC1)CCC)c2c3c(nccc3)ccc2 | 0.55 |
| 215 | S1C2=NC(=C(N2C=C1)C(=O)NC[C@H]3[C@@H]([NH+]4CCCC4)CCCC3)C | 0.813 | 1 | -33.35 | 1.00 | S1C2=NC(=C(N2C=C1)C(=O)NCC3C(N4CCCC4)CCCC3)C | 0.50 |
| 216 | Brc1cc2OCCOc2c(c1)C(=O)NCC[NH+](CCC)CC | 0.750 | 1 | -33.35 | 1.00 | Brc1cc2OCCOc2c(c1)C(=O)NCCN(CCC)CC | 0.45 |
| 217 | O=C(NCC[NH+](C(C)C)CCC)c1c2N(C=Nc2ccc1)C | 0.786 | 1 | -33.34 | 1.00 | O=C(NCCN(C(C)C)CCC)c1c2N(C=Nc2ccc1)C | 0.45 |
| 218 | O=C(NCC1(C[NH+](C)C)CCC1)c2c(nc(cc2)C(C)C)CC | 0.750 | 1 | -33.34 | 1.00 | O=C(NCC1(CN(C)C)CCC1)c2c(nc(cc2)C(C)C)CC | 0.41 |
| 219 | O=C(NCC([NH+]1CCCC1)(C)C)C=2c3c(N(C2)CC)cccc3 | 0.750 | 1 | -33.28 | 1.00 | O=C(NCC(N1CCCC1)(C)C)C=2c3c(N(C2)CC)cccc3 | 0.46 |
| 220 | O=C(NCC[NH+](C1CC1)C(C)C)c2c(ncnc2)C(C)C | 0.750 | 1 | -33.28 | 1.00 | O=C(NCCN(C1CC1)C(C)C)c2c(ncnc2)C(C)C | 0.44 |
| 221 | Clc1cc(Cl)cc(c1)C(=O)NCC[NH+](CCC)CC | 0.800 | 1 | -33.27 | 1.00 | Clc1cc(Cl)cc(c1)C(=O)NCCN(CCC)CC | 0.47 |
| 222 | O=C(NCC[NH+](C(C)C)CCC)C=1NC(=CC1C)C | 0.786 | 1 | -33.23 | 0.95 | O=C(NCCN(C(C)C)CCC)C=1NC(=CC1)C | 0.40 |
| 223 | O=C(NCC(c1ncccc1)(C)C)[C@H]2C[NH+](CC(C)C)CCC2 | 0.929 | 1 | -33.22 | 0.96 | Fc1c(nccc1)C(CNC(=O)C2CN(CC(C)C)CCC2)(C)C | 0.44 |
| 224 | O=C(NC(C[NH+](CCC)C)(C)C)c1c2c(N=C(C2(C)C)C)ccc1 | 0.824 | 1 | -33.21 | 0.92 | O=C(NC(CN(CCCC)C)(C)C)c1c2c(N=C(C2(C)C)C)ccc1 | 0.42 |
| 225 | O=C(NCCC=1ON=C(N1)C(C)C)C[C@H]2C[NH+](C(C)C)CCC2 | 0.765 | 1 | -33.19 | 0.96 | O=C(NCCC=1ON=C(N1)CC)CC2CN(C(C)C)CCC2 | 0.45 |
| 226 | O=C(NCC[NH+]1[C@H](CCC1)C)c2c(NC(=O)C(C)C)cccc2 | 0.750 | 1 | -33.15 | 0.96 | O=C(NCCN1C(CCC1)C)c2c(NC(=O)CC)cccc2 | 0.46 |
| 227 | O=C(NC(C[NH+](CCC)C)(C)C)C1=NN(C2=C1CCCC2)C | 0.765 | 1 | -33.14 | 1.00 | O=C(NC(CN(CCC)C)(C)C)C1=NN(C2=C1CCCC2)C | 0.44 |
| 228 | S1C=C(c2ccccc2)C(C(=O)NCC[NH+](C3CC3)C(C)C)=C1 | 0.750 | 1 | -33.11 | 1.00 | S1C=C(c2ccccc2)C(C(=O)NCCN(C3CC3)C(C)C)=C1 | 0.54 |
| 229 | O=C(NC[C@H]1[C@@H]([NH+]2CCCC2)CCCC1)C3=C(N(C=C3)C)c4ccccc4 | 0.765 | 1 | -33.10 | 1.00 | O=C(NCC1C(N2CCCC2)CCCC1)C3=C(N(C=C3)C)c4ccccc4 | 0.46 |
| 230 | O=C(NCC[NH+](C1CC1)C(C)C)C=2c3c(OC2)cc(O)cc3 | 0.800 | 1 | -33.06 | 0.95 | O=C(NCCN(C1CC1)C(C)C)C=2c3c(OC2)cccc3 | 0.44 |
| 231 | O=C(NCC[NH+](C(C)C)CCC)c1c2ncc(nc2ccc1)C | 0.867 | 1 | -32.98 | 0.96 | O=C(NCCN(C(C)C)CCC)c1c2nccnc2ccc1 | 0.44 |
| 232 | O=C(NCC[NH+]1[C@H](CCC1)C)c2c3c(cc4c2CCC4)CCC3 | 0.800 | 1 | -32.96 | 0.87 | O=C(NCCN1C(CCC1)C)c2c3c(ccc2)CCC3 | 0.43 |
| 233 | O=C(NCC[NH+]1[C@H](CCC1)C)c2c3c(ccc2)CCC3 | 0.800 | 1 | -32.94 | 1.00 | O=C(NCCN1C(CCC1)C)c2c3c(ccc2)CCC3 | 0.47 |
| 234 | O=C(NC1(C[NH+](CC)CC)CCCC1)[C@@H]2CN3C(=NN=C3CC2)C4CC4 | 0.778 | 1 | -32.92 | 0.88 | O=C(NC1(CN(CC)CC)CCCC1)C2CN3C(=NN=C3)CC2 | 0.46 |
| 235 | O=C(NC[C@H]1[NH+](CCC1)CCC)C2(C(=O)c3ccccc3)CC2 | 0.929 | 1 | -32.86 | 1.00 | O=C(NCC1N(CCC1)CCC)C2(C(=O)c3ccccc3)CC2 | 0.56 |

| | | | | | | | |
|---|---|---|---|---|---|---|---|
| 236 | O=C(NC(C[NH+](CC)CC)(CC)CC)c1c2NC3=C(c2ccc1)CCC3 | 0.813 | 1 | -32.83 | 0.89 | O=C(NC(CN(C(=O)C)CC)CC)c1c2NC3=C(c2ccc1)CCC3 | 0.46 |
| 237 | O=C(NC(C[NH+](CC)CC)(CC)CC)c1c(ncc2c1cccc2)C | 0.765 | 1 | -32.78 | 0.96 | O=C(NC(CN(CC)CC)(CC)CC)c1c2c(cnc1)cccc2 | 0.45 |
| 238 | O=C(NC[C@H]([NH+]1CCCC1)C)C=2C(=NN(C2)C(C)C)C3CC3 | 0.813 | 1 | -32.77 | 0.95 | O=C(NCC(N1CCCC1)C)C=2C(=NN(C2)CC)C3CC3 | 0.40 |
| 239 | O=C(NC[C@H]1[NH+](CCC1)CCC)C=2C(=NOC2C)c3ccccc3 | 0.929 | 1 | -32.73 | 1.00 | O=C(NCC1N(CCC1)CCC)C=2C(=NOC2C)c3ccccc3 | 0.54 |
| 240 | S(C1=NN(C=C1C(=O)NCC[NH+](C2CC2)C(C)C)C)C | 0.765 | 1 | -32.70 | 0.90 | S(C=1N(N=CC1C(=O)NCCN(C2CC2)C(C)C)C)C | 0.41 |
| 241 | O=C(NCc1c(nccc1)N2CCOCC2)C[C@H]3[NH+](CCC3)CC | 0.813 | 1 | -32.68 | 0.96 | O=C(N1C(CC(=O)NCc2c(nccc2)N3CCOCC3)CCC1)C | 0.44 |
| 242 | O=C(NC[C@H]1[NH+](CCC1)CCC)c2c(ncnc2)C3CC3 | 0.786 | 1 | -32.61 | 1.00 | O=C(NCC1N(CCC1)CCC)c2c(ncnc2)C3CC3 | 0.56 |
| 243 | Fc1ccc(cc1)C2(C(=O)NCC[NH+](C(C)C)CCC)CCC2 | 1.000 | 1 | -32.57 | 1.00 | Fc1ccc(cc1)C2(C(=O)NCCN(C(C)C)CCC)CCC2 | 0.51 |
| 244 | O=C(NCC[NH+](C(C)C)CC)C=1C(=NN(C1)C)c2ccncc2 | 0.857 | 1 | -32.44 | 0.96 | n1ccc(cc1)C2=NN(C=C2CNCCN(C(C)C)CC)C | 0.46 |
| 245 | Brc1nc(Cl)cc(c1)C(=O)NCC[NH+](C2CC2)C(C)C | 0.875 | 1 | -32.38 | 0.95 | Brc1nc(Cl)cc(c1N)C(=O)NCCN(C2CC2)C(C)C | 0.38 |
| 246 | O=C(NC[C@H]1[NH+](CCC1)CCC)c2cc(OC)ccc2 | 0.857 | 1 | -32.36 | 1.00 | O=C(NCC1N(CCC1)CCC)c2cc(OC)ccc2 | 0.59 |
| 247 | O=C(NC1(C[NH+](CC)CC)CCCC1)c2c3c(cc(O)cc3)ccc2 | 0.750 | 1 | -32.30 | 1.00 | O=C(NC1(CN(CC)CC)CCCC1)c2c3c(cc(O)cc3)ccc2 | 0.47 |
| 248 | Fc1cc2c(c(c1)C(=O)NC[C@H]3[NH+](CCC3)CCC)cccc2 | 0.800 | 1 | -32.23 | 0.96 | O=C(NCC1N(CCC1)CCC)c2c3c(ccc2)cccc3 | 0.56 |
| 249 | O=C(NCC[NH+](CCC)CC)C=1C(=NOC1C)c2ccccc2 | 0.750 | 1 | -32.14 | 1.00 | O=C(NCCN(CCC)CC)C=1C(=NOC1C)c2ccccc2 | 0.50 |
| 250 | O=C(NC[C@H]1[NH+](C2CC2)CCC1)c3c4c(nc(c3)C)N(N=C4)C | 0.857 | 1 | -32.09 | 0.88 | O=C(NCC1N(C2CC2)CCCC1)c3c4c(nc(c3)C)N(N=C4)C | 0.48 |
| 251 | O=C(NC1(C[NH+](CC)CC)CCCC1)c2c3c(ccc2)COC3 | 0.750 | 1 | -32.08 | 0.92 | O=C(NC1(CN(CC)CC)CCCC1)c2c3c(ccc2)CCC3 | 0.42 |
| 252 | O=C(NC[C@@H]([NH+]1CCCC1)c2ccc(cc2)C)c3c4c(nc(c3)C)N(N=C4)C | 0.765 | 1 | -32.08 | 1.00 | O=C(NCC(N1CCCC1)c2ccc(cc2)C)c3c4c(nc(c3)C)N(N=C4)C | 0.44 |
| 253 | Fc1cc2ncc(O)c(c2cc1)C(=O)NCC[NH+](CCC)CCC | 0.750 | 1 | -32.05 | 0.92 | Fc1c(F)ccc2c1nc(c(O)c2C(=O)NCCN(CCC)CCC)C | 0.48 |
| 254 | Brc1c(ncc(c1)C)[C@@H](NC(=O)C2(C[NH+]3CCCC3)CCCC2)C | 0.824 | 1 | -32.02 | 0.81 | Brc1c(ncc(c1)C)C(NC(=O)C2(C(=O)N3CCCC3)CC2)C | 0.41 |
| 255 | O=C(NC[C@H]1C[NH+](C(C)C)CCC1)c2cc3c(OCOC3)cc2 | 0.867 | 1 | -32.01 | 0.92 | O=C(NCC1CN(C(C)C)CCC1)c2cc3c(OC(OC3)(C)C)cc2 | 0.49 |
| 256 | O=C(NCC[NH+](CCC)CCC)c1c2N=C(Oc2ccc1)C | 0.813 | 1 | -32.01 | 1.00 | O=C(NCCN(CCC)CCC)c1c2N=C(Oc2ccc1)C | 0.45 |
| 257 | Clc1nc(cc(c1)C(=O)NC[C@H]2C[NH+](CCC2)CC)CC | 0.750 | 1 | -31.99 | 0.95 | O=C(NCC1CN(CCC1)CC)c2cc(ncc2)CC | 0.48 |
| 258 | O=C(OC(C)(C)C)c1c(nccc1)C(=O)NC[C@@H]([NH+]2CCCC2)C | 0.813 | 1 | -31.92 | 1.00 | O=C(OC(C)(C)C)c1c(nccc1)C(=O)NCC(N2CCCC2)C | 0.44 |
| 259 | O=C(NCC1([NH+](C)C)CCCCC1)CC2(c3ccccc3)CC2 | 0.867 | 1 | -31.89 | 0.96 | Brc1ccc(cc1)C2(CC(=O)NCC3(N(C)C)CCCCC3)CC2 | 0.41 |
| 260 | O=C(NCC[NH+](C(C)C)CCC)c1c2c(OC(=C2)C)ccc1 | 0.933 | 1 | -31.87 | 1.00 | O=C(NCCN(C(C)C)CCC)c1c2c(OC(=C2)C)ccc1 | 0.44 |
| 261 | S(C1=NN(C=C1C(=O)NC[C@H]2[NH+](CCC2)CCC)C)C | 0.857 | 1 | -31.84 | 0.90 | S(C=1N(N=CC1C(=O)NCC2N(CCC2)CCC)C)C | 0.52 |
| 262 | O=C(NC[C@@H]([NH+]1CCCC1)C(C)C)c2c3c(ncc2)CCC3 | 0.765 | 1 | -31.79 | 0.91 | O=C(NCC(N1CCCC1)C)c2c3c(ncc2)CCC3 | 0.42 |
| 263 | Brc1nc(cc(c1)C(=O)NCC[NH+](CCC)CC)C | 0.800 | 1 | -31.78 | 0.95 | O=C(NCCN(CCC)CC)c1cc(ncc1)C | 0.40 |
| 264 | O=C(NCC[NH+](CCC)CC)c1c(cc2OCOc2c1)C | 0.800 | 1 | -31.72 | 1.00 | O=C(NCCN(CCC)CC)c1c(cc2OCOc2c1)C | 0.46 |
| 265 | Fc1cc2OCC[C@H](NC(=O)CC[NH+](CC)CC)c2cc1 | 0.813 | 1 | -31.71 | 1.00 | Fc1cc2OCCC(NC(=O)CCN(CC)CC)c2cc1 | 0.46 |

| | | | | | | | |
|---|---|---|---|---|---|---|---|
| 266 | O=C(NCC[NH+](CCC)CC)C1=NNC=C1c2ccccc2 | 0.786 | 1 | -31.71 | 1.00 | O=C(NCCN(CCC)CC)C1=NNC=C1c2ccccc2 | 0.55 |
| 267 | Brc1cc2SC=Nc2c(c1)C(=O)NC(C[NH+](CC)CC)(CC)CC | 0.778 | 1 | -31.61 | 0.96 | S1c2c(N=C1)c(ccc2)C(=O)NC(CN(CC)CC)(CC)CC | 0.40 |
| 268 | O=C(NC[C@H]1[NH+](C2CC2)CCC1)c3c4c(ccc3)CCOC4 | 0.857 | 1 | -31.57 | 0.88 | O=C(NCC1N(C2CC2)CCCC1)c3c4c(ccc3)CCOC4 | 0.48 |
| 269 | ClC1=CN(C(C(=O)NCC[NH+](C2CC2)C(C)C)=C1)C | 0.800 | 1 | -31.54 | 1.00 | ClC1=CN(C(C(=O)NCCN(C2CC2)C(C)C)=C1)C | 0.44 |
| 270 | S1c2c(cc(cc2)C)C(C(=O)NC3(C[NH+](CC)CC)CCCC3)=C1 | 0.765 | 1 | -31.51 | 1.00 | S1c2c(cc(cc2)C)C(C(=O)NC3(CN(CC)CC)CCCC3)=C1 | 0.41 |
| 271 | O=C(NC[C@H]1C[NH+](CCC1)CC)C=2N(c3c(cccc3)C2)CCC | 0.933 | 1 | -31.38 | 1.00 | O=C(NCC1CN(CCC1)CC)C=2N(c3c(cccc3)C2)CCC | 0.51 |
| 272 | Brc1cc2SC=Nc2c(c1)C(=O)NCC[NH+](CCC)CCC | 0.800 | 1 | -31.38 | 0.95 | S1c2c(N=C1)c(ccc2)C(=O)NCCN(CCC)CCC | 0.44 |
| 273 | Fc1cnc(cc1)C2(CNC(=O)[C@H]3C[NH+](CC(C)C)CCC3)CCC2 | 0.857 | 1 | -31.32 | 0.96 | O=C(NCC1(c2ncccc2)CCC1)C3CN(CC(C)C)CCC3 | 0.47 |
| 274 | Fc1c(cc(cc1)C2CC2)C(=O)NC[C@H]3[NH+](CCC3)CCC | 0.813 | 1 | -31.32 | 1.00 | Fc1c(cc(cc1)C2CC2)C(=O)NCC3N(CCC3)CCC | 0.56 |
| 275 | Clc1c(c(ccc1)C(=O)NC(C[NH+](CC)CC)(CC)CC)C | 0.750 | 1 | -31.29 | 1.00 | Clc1c(c(ccc1)C(=O)NC(CN(CC)CC)(CC)CC)C | 0.45 |
| 276 | O=C(NC[C@H]1[C@@H]([NH+]2CCCC2)CCCC1)c3c(NC(=O)C(C)C)cccc3 | 0.813 | 1 | -31.26 | 1.00 | O=C(NCC1C(N2CCCC2)CCCC1)c3c(NC(=O)C(C)C)cccc3 | 0.45 |
| 277 | Clc1c(O)c(cc(Cl)c1)C(=O)NC[C@H]2[NH+](CCC2)CCC | 0.786 | 1 | -31.24 | 1.00 | Clc1c(O)c(cc(Cl)c1)C(=O)NCC2N(CCC2)CCC | 0.66 |
| 278 | Brc1cc(c(c(c1)C)C)C(=O)NC[C@H]2[NH+](CCC2)CCC | 0.857 | 1 | -31.21 | 1.00 | Brc1cc(c(c(c1)C)C)C(=O)NCC2N(CCC2)CCC | 0.62 |
| 279 | O=C(NC[C@H]1[NH+](C2CC2)CCC1)c3c(cnc(c3)C)C | 0.786 | 1 | -31.19 | 0.86 | O=C(NCC1N(C2CC2)CCCC1)c3c(cnc(c3)C)C | 0.50 |
| 280 | O=C(N[C@H]1C2=NC(=NN2CCC1)CC)C[C@]([NH+](C)C)(C3CCC3)C | 0.750 | 1 | -31.14 | 1.00 | O=C(NC1C2=NC(=NN2CCC1)CC)CC(N(C)C)(C3CCC3)C | 0.38 |
| 281 | BrC=1N=C(N2C1CCCC2)C(=O)NC[C@H]3C[NH+](C(C)C)CCC3 | 0.750 | 1 | -31.13 | 1.00 | BrC=1N=C(N2C1CCCC2)C(=O)NCC3CN(C(C)C)CCC3 | 0.46 |
| 282 | O=C(NCC[NH+](CCC)CC)c1cc(NC(=O)C)ccc1 | 0.750 | 1 | -31.12 | 1.00 | O=C(NCCN(CCC)CC)c1cc(NC(=O)C)ccc1 | 0.46 |
| 283 | Clc1c(O)c(Cl)cc(c1)C(=O)N[C@H](C[NH+](CCC)CC)C | 0.750 | 1 | -31.05 | 0.95 | Clc1c(O)c(Cl)cc(c1)C(=O)NC(CN(C(=O)CC)CC)C | 0.46 |
| 284 | O=C(NCC[C@@H]([NH+]1CCC1)C)C2=C(OC(=C2C)C)C | 0.813 | 1 | -31.05 | 0.95 | O=C(NCCCN1CCC1)C2=C(OC(=C2C)C)C | 0.45 |
| 285 | S1C2=NC(=C(N2C=C1C)C(=O)NC[C@H]3C[NH+](CCC3)CC)CC | 0.857 | 1 | -31.05 | 1.00 | S1C2=NC(=C(N2C=C1C)C(=O)NCC3CN(CCC3)CC)CC | 0.45 |
| 286 | O=C(N[C@@H](C[NH+](CCC)CC)C)C=1C(=NNC1)c2ccccc2 | 0.750 | 1 | -31.00 | 0.96 | O=C(NC(CN(CC)CC)C)C=1C(=NNC1)c2ccccc2 | 0.47 |
| 287 | O=C(Nc1c(C#N)ccc(N2C[C@H]([NH+](C)C)CCC2)c1)CC=C=C | 0.813 | 1 | -30.98 | 0.83 | O=C(Nc1c(ccc(N2CCCCC2)c1)C)CC=C=C | 0.45 |
| 288 | O=C(NCC=1NN=CC1C2CCC2)[C@H]3C[NH+](CCC3)CC | 0.786 | 1 | -30.91 | 1.00 | O=C(NCC1=NNC=C1C2CCC2)C3CN(CCC3)CC | 0.43 |
| 289 | O=C(NCC[NH+](C1CC1)C(C)C)C=2NN=CC2C(C)(C)C | 0.750 | 1 | -30.65 | 0.91 | O=C(NCCN(C1CC1)C(C)C)C2=NOC=C2C(C)(C)C | 0.43 |
| 290 | Fc1ccc(cc1)C2(C(=O)NC[C@@H]3C[NH+](CCCC3)C)CCCC2 | 0.800 | 1 | -30.62 | 0.96 | Fc1ccc(cc1)C2(C(=O)NCC3CNCCCC3)CCCC2 | 0.43 |
| 291 | O=C(NC[C@@H]([NH+]1CCCC1)C(C)C)c2c(cccc2)CC(C)C | 0.765 | 1 | -30.55 | 0.96 | N1(C(C(C)C)CNCc2c(cccc2)CC(C)C)CCCC1 | 0.49 |
| 292 | O=C(NC[C@H]([NH+]1CCCC1)C)C=2C(=NN(C2)C)C3CCOCC3 | 0.750 | 1 | -30.54 | 0.96 | O=C(NCC(N1CCCC1)C)C=2C(=NNC2)C3CCOCC3 | 0.43 |
| 293 | O=C(NC1(C[NH+](CC)CC)CCCC1)c2cc(ccc2)CC#N | 0.750 | 1 | -30.50 | 1.00 | O=C(NC1(CN(CC)CC)CCCC1)c2cc(ccc2)CC#N | 0.44 |
| 294 | Brc1cc2c(SC=C2C(=O)NCC[NH+](CCC)CC)cc1 | 0.813 | 1 | -30.49 | 1.00 | Brc1cc2c(SC=C2C(=O)NCCN(CCC)CC)cc1 | 0.45 |
| 295 | O=C(NCC[NH+](CCC)CC)C1=NOC2=C1C[C@H](C(C)(C)C)CC2 | 0.813 | 1 | -30.49 | 1.00 | O=C(NCCN(CCC)CC)C1=NOC2=C1CC(C(C)(C)C)CC2 | 0.42 |

| | | | | | | | |
|---|---|---|---|---|---|---|---|
| 296 | O=C(NCC[NH+](CCC)CC)C1=C(N(N=C1)C)CC | 0.750 | 1 | -30.46 | 1.00 | O=C(NCCN(CCC)CC)C1=C(N(N=C1)C)CC | 0.50 |
| 297 | O=C(N[C@@H](C[NH+]1CCCC1)C)c2cc(OC(C)(C)C)ccc2 | 0.765 | 1 | -30.38 | 1.00 | O=C(NC(CN1CCCC1)C)c2cc(OC(C)(C)C)ccc2 | 0.45 |
| 298 | S1C2=C(C=C1)[C@@H](C(=O)NC(C[NH+](CC)CC)(CC)CC)CCC2 | 0.750 | 1 | -30.37 | 1.00 | S1C2=C(C=C1)C(C(=O)NC(CN(CC)CC)(CC)CC)CCC2 | 0.43 |
| 299 | O=C(NCC[NH+]1C[C@@H](C(C)C)CC1)C=2C(=NOC2C)c3ccccc3 | 0.765 | 1 | -30.34 | 0.92 | O=C(NCCN1CC(CC1)C)C=2C(=NOC2C)c3ccccc3 | 0.50 |
| 300 | O=C(NC[C@H]1C[NH+](CCC1)CC)c2c(c(ccc2)C)CC | 0.800 | 1 | -30.34 | 0.95 | O=C(NCC1CN(CCC1)CC)c2c(c(ccc2)C)C | 0.51 |
| 301 | O=C(NCC[NH+](CCC)CC)c1c2NC(=Nc2cnc1)C | 0.857 | 1 | -30.33 | 0.95 | O=C(NCCN(CCC)CC)c1c2N=CNc2cnc1 | 0.51 |
| 302 | S(c1c(ccnc1)C(=O)NC2(C[NH+](CC)CC)CCCC2)CC | 0.813 | 1 | -30.30 | 1.00 | S(c1c(ccnc1)C(=O)NC2(CN(CC)CC)CCCC2)CC | 0.42 |
| 303 | O=C(NC[C@H]1[NH+](CCCC1)CC)C2=C(NC=C2)C3CCCC3 | 0.800 | 1 | -30.30 | 1.00 | O=C(NCC1N(CCCC1)CC)C2=C(NC=C2)C3CCCC3 | 0.57 |
| 304 | Brc1c(OC)cc(nc1)C(=O)NCC[NH+](CCC)CC | 0.750 | 1 | -30.29 | 0.95 | O=C(NCCN(CCC)CC)c1nccc(OC)c1 | 0.53 |
| 305 | O=C(O[C@H](c1ccccc1)C(=O)NCCC[NH+](CC)CC)C | 0.778 | 1 | -30.28 | 1.00 | O=C(OC(c1ccccc1)C(=O)NCCCN(CC)CC)C | 0.48 |
| 306 | O=C(NC[C@H]1C[NH+](CC(C)C)CCC1)c2c(cc(cc2)C)CC | 1.000 | 1 | -30.22 | 0.96 | O=C(NCC1CN(CC(C)C)CCC1)c2c(cc(cc2)C)C | 0.51 |
| 307 | O=C(NC(C[NH+](CCC)C)(C)C)c1nc(OC)c2c(c1)cccc2 | 0.750 | 1 | -30.22 | 1.00 | O=C(NC(CN(CCC)C)(C)C)c1nc(OC)c2c(c1)cccc2 | 0.41 |
| 308 | O=C(NC[C@H]1[NH+](CCC1)CCC)C=2C(=NNC2)C3CCCCC3 | 0.750 | 1 | -30.21 | 1.00 | O=C(NCC1N(CCC1)CCC)C=2C(=NNC2)C3CCCCC3 | 0.57 |
| 309 | O=C(NCC[NH+](C(C)C)CCC)c1c2c(ncc1)ccc(O)c2 | 0.800 | 1 | -30.18 | 1.00 | O=C(NCCN(C(C)C)CCC)c1c2c(ncc1)ccc(O)c2 | 0.45 |
| 310 | Brc1c(cc(nc1)C)C(=O)NCC[NH+](C2CC2)C(C)C | 0.750 | 1 | -30.14 | 0.95 | Brc1c(ccnc1)C(=O)NCCN(C2CC2)C(C)C | 0.40 |
| 311 | O=C(NCC[NH+]1[C@H](CCC1)C)c2c3c(N=C(C3(C)C)C)ccc2 | 0.750 | 1 | -30.10 | 0.96 | O=C(NCCN1CCCC1)c2c3c(N=C(C3(C)C)C)ccc2 | 0.48 |
| 312 | O=C(NC[C@@H]1C[NH+](CCCC1)C)C2(c3ccccc3)CC4(C2)CC4 | 0.857 | 1 | -30.08 | 0.92 | Fc1c(cccc1)C2(C(=O)NCC3CNCCCC3)CC4(C2)CC4 | 0.47 |
| 313 | O=C(NCC[NH+](C1CC1)C(C)C)c2ncccc2c3ncccc3 | 0.813 | 1 | -30.02 | 0.85 | O=C(NCCN(C1CC1)C(C)C)c2c(c3ccccc3)cccc2 | 0.45 |
| 314 | O=C(NC(C[NH+](CCC)C)(C)C)c1c2c(OC(=C2)C)ccc1 | 0.750 | 1 | -30.01 | 1.00 | O=C(NC(CN(CCC)C)(C)C)c1c2c(OC(=C2)C)ccc1 | 0.38 |
| 315 | O=C(NCC[NH+]1[C@H](CCC1)C)c2c(cccc2C)CC | 0.750 | 1 | -30.00 | 0.95 | O=C(NCCN1C(CCC1)C)c2c(cccc2)CC | 0.53 |

[a] Interaction fingerprint similarity to the X-ray pose of eticlopride (all interactions), [b] Interaction fingerprint similarity to the X-ray pose of eticlopride (h-bonds, ionic bonds), [c] HYDE score (kJ/mol), [d] maximum common substructure similarity to the closest REAL space ligand, [e] SMILES string of the closest ligand in REAL space, [f] ECFP4 similarity to the closest DRD2/DRD3 ligands.

**Table S6.** Parameter settings for PLANTS docking

| | |
|---|---|
| # scoring function and search settings | |
| scoring_function | chemplp |
| search_speed | speed1 |
| | |
| # input | |
| protein_file | protein.mol2 *# protein in mol2 file format* |
| ligand_file | bb.mol2 *# reagent to dock in mol2 file format* |
| # output | |
| output_dir | results #output directory |
| write_protein_conformations | 1 |
| | |
| # write single mol2 files | |
| write_multi_mol2 | 0 |
| | |
| # binding site definition | |
| bindingsite_center | 9.15 36.39 -3.34 *# center of mass of the cavity* |
| bindingsite_radius | 10 |
| | |
| # cluster algorithm | |
| cluster_structures | 20 |
| cluster_rmsd | 1.0 |

**Table S7.** Parameter settings for GOLD docking

AUTOMATIC SETTINGS
autoscale = 1

POPULATION
popsiz = auto
select_pressure = auto
n_islands = auto
maxops = auto
niche_siz = auto

GENETIC OPERATORS
pt_crosswt = auto
allele_mutatewt = auto
migratewt = auto

FLOOD FILL
radius =  10
origin = 9.15 36.39 -3.34
do_cavity = 1
floodfill_atom_no = 0
cavity_file =  ligand.mol2
floodfill_center = cavity_from_ligand

DATA FILES
ligand_data_file reagent.mol2 20
param_file = DEFAULT
set_ligand_atom_types = 1
set_protein_atom_types = 1
directory = ./results
tordist_file = DEFAULT
make_subdirs = 0
save_lone_pairs = 0
fit_points_file = fit_pts.mol2
read_fitpts = 0

FLAGS
internal_ligand_h_bonds = 0
flip_free_corners = 0
match_ring_templates = 0
flip_amide_bonds = 0
flip_planar_n = 1 flip_ring_NRR flip_ring_NHR
flip_pyramidal_n = 0
rotate_carboxylic_oh = flip
use_tordist = 1
postprocess_bonds = 1
rotatable_bond_override_file = DEFAULT
solvate_all = 1
diverse_solutions = 1
divsol_cluster_size = 1

divsol_rmsd = 1

TERMINATION
early_termination = 0

CONSTRAINTS
force_constraints = 0

COVALENT BONDING
covalent = 0

SAVE OPTIONS
save_score_in_file = 1
save_protein_torsions = 1

FITNESS FUNCTION SETTINGS
initial_virtual_pt_match_max = 3
relative_ligand_energy = 1
gold_fitfunc_path = **plp # or goldscore, or chemscore**
score_param_file = DEFAULT

PROTEIN DATA
protein_datafile = protein.mol2

**Table S8.** Parameter settings for RDPSOVina docking, Surflex-Dock and FlexX docking

**#RDPS0Vina**

prepare_receptor -r protein.mol2 -o protein.pdbqt *# prepare-receptor available in ADFRsuite-1.0*
prepare_ligand -l reagent.mol2 -o reagent.pdbqt *# prepare_ligand available in ADFRsuite-1.0*
rdpsovina --receptor protein.pdbqt –ligand reagent.pdbqt --center_x 9.15 --center_y 36.39 --center_z -3.34 --size_x 20 --size_y 20 --size_z 20 --out docked.pdbqt --log docked.log --energy_range 8 --exhaustiveness 25

**#SURFLEX-DOCK**

#protomol generation
sfdock proto ligand.mol2 protein.mol2 p
mv p-protomol.mol2 protomol.mol2
mv p-corevox.mol2 corevox.mol2

#Prepare building block
sftools -pgeom forcegen reagent-random.mol2 reagent

#Dock ligand
sfdock -pgeom -ndock_final 20 dock reagent.sfdb protomol.mol2 corevox.mol2 protein.mol2

**#FlexX**

/soft/biosolveit/FlexX/v5.2.0/flexx --input reagent.mol2 --protein protein.pdb --output reagent_docked.sdf --refligand ligand.mol2 --max-nof-conf 20
