# Supplementary material for: Protein Structure-Based Organic Chemistry-Driven Ligand Design from Ultralarge Chemical Spaces
Source: ACS Cent Sci. 2024 Feb 13;10(3):615–27. doi: 10.1021/acscentsci.3c01521 (PMC10979501; doi:10.1021/acscentsci.3c01521)
Supplement: Supplementary file 2 — oc3c01521_si_002.pdf [file oc3c01521_si_002.pdf]

oc-2023-01521a.R1

Name: Peer Review Information for "Protein structure-based organic chemistry-driven ligand design from ultra-large chemical spaces"

First Round of Reviewer Comments

Reviewer: 1

Comments to the Author

This is a very interesting contribution where the authors used a structure-based approach for screening of an ultra-large chemical space. The novelty of the SpaceDock approach lies in the fact that the authors use chemical building blocks as is as fragments for docking instead of docking fragments with exit vectors. On the bases of two use cases (Estrogen receptor beta and dopamine receptor D3) they demonstrate that, after well documented rigorous filtering steps, a considerable number of high quality hits were obtained. Run times are moderate, which allows structure-based enumeration of billions of compounds in reasonable time frames.

Reviewer: 2

Comments to the Author

The paper presents SpaceDock, an interesting and exceptionally efficient approach to provide ultralarge chemically accessible libraries on the fly by linking commercially available reactants by one or two synthetical steps, based on docking of the said reactants. The code is made freely available, which is highly appreciated.I have just minor questions and comments to the authors.

Comments

* I suggest expliciting which receptors have been used to test the method in the abstract.

* Please revise "One the one hand, brute force docking of 1.4 billion compounds".

* Does SpaceDock consider those chemical functions that will be later involved in the linker reaction when the reactants are initially docked? If so, do the authors think that this may compromise the affinity of the final product of the reaction? I would suggest to discuss this, depending on the answer, in the "Preparation of bespoke chemical spaces encoded by 36 robust organic chemistry reactions" section or in the discussion.

* In their application examples, the authors examine the similarity odf the hits to known ligands of the pursued target (E.g., "About half of the hits (30 out 64; all from the benzoxazole space), were considered chemically similar..."). How is such similarity evaluated?

* The authors state that "SpaceDock remains a relatively light computational procedure since browsing a chemical space of 100 million compounds can be achieved within 2 days on a 16-core Intel(R) Xeon(R) Silver 4210 processor". And just after that "Mining the entire 5.5 billion chemical space has been made..". What is the entire chemical space that they are referring to there?

* Revise "Poisening functions" in table S2.

Author's Response to Peer Review Comments:

ACS Central Science

Editorial board

Manuscript oc-2023-01521a

Didier Rognan
Directeur

Laboratoire d'Innovation Thérapeutique
UMR 7200
CNRS-Université de Strasbourg
74, route du Rhin, BP 24
F-67401 Illkirch, France

Tél. : (33) 03 68 85 42 35
Fax : (33) 03 68 85 43 10
rognan@unistra.fr
http://medchem.u-srasbg.fr

Dear Editor,

Please find here a revised version of the manuscript entitled " *Protein structure-based organic chemistry-driven ligand design from ultra-large chemical spaces* ".
We have taken into account all remarks of both reviewers and reformatted references, web sources and the supporting information as requested (see below).

We do hope that the revised version is now suitable for publication in ACS Central Science. Should you have any additional remarks, please let me know.

Kind regards

Dr Didier ROGNAN
Directeur du Laboratoire
d'Innovation Thérapeutique
UMR 7200 - UNISTRA/CNRS

Didier ROGNAN, PhD

Formatting Needs

1) SI PARAGRAPH: If the manuscript is accompanied by any supporting information for publication, a brief description of the supplementary material is required in the manuscript. The appropriate format is: Supporting Information. Brief statement in non-sentence format listing the contents of the material supplied as Supporting Information.

The content of the SI material is now provided page 26 of the revised section

2) GENERAL REF FORMATTING: Periodical references should contain authors' surnames followed by initials, article title, journal abbreviation, year, volume number, and page range. Refs with more than 10 authors should list the first 10 and then be followed by "et al."

Web sources must include access date.

References and web sources have been updated accordingly.

3) SI PG#S: The supporting information pages must be numbered consecutively, starting with page S1.

The pagination of the supporting information has been modified as requested by the editor.

4) typos were corrected pages 4 and 35

Reviewer: 1

This is a very interesting contribution where the authors used a structure-based approach for screening of an ultra-large chemical space. The novelty of the SpaceDock approach lies in the fact that the authors use chemical building blocks as is as fragments for docking instead of docking fragments with exit vectors. On the bases of two use cases (Estrogen receptor beta and dopamine receptor D3) they demonstrate that, after well documented rigorous filtering steps, a considerable number of high-quality hits were obtained. Run times are moderate, which allows structure-based enumeration of billions of compounds in reasonable time frames.

We thank the reviewer for the very nice comments on our manuscript

Reviewer: 2

The paper presents SpaceDock, an interesting and exceptionally efficient approach to provide ultralarge chemically accessible libraries on the fly by linking commercially available reactants by one or two synthetical steps, based on docking of the said reactants. The code is made freely available, which is highly appreciated. I have just minor questions and comments to the authors.

Comments

1) I suggest expliciting which receptors have been used to test the method in the abstract.

The two receptors used in the current study have been explicitly mentioned page 2, in the revised abstract.

2) Please revise "One the one hand, brute force docking of 1.4 billion compounds".

"brute force" has been replaced by "exhaustive" page 3 of the revised manuscript.

3) Does SpaceDock consider those chemical functions that will be later involved in the linker reaction when the reactants are initially docked? If so, do the authors think that this may compromise the affinity of the final product of the reaction? I would suggest to discuss this, depending on the answer, in the "Preparation of bespoke chemical spaces encoded by 36 robust organic chemistry reactions" section or in the discussion.

Of course, the chemical moieties engaged in the organic chemistry reaction are considered during the initial docking step. In case a function is wrongly posed and/or strongly interacting with the target, it might not be available for further linking if topological and chemical compatibility with the second posed reactant is no more verified.

We thank the reviewer for suggesting this clarification that has been added page 18 of the revised version.

4) In their application examples, the authors examine the similarity of the hits to known ligands of the pursued target (E.g., "About half of the hits (30 out 64; all from the benzoxazole space), were considered chemically similar..."). How is such similarity evaluated?

2D pairwise similarity was estimated by the Tanimoto coefficient measured on circular ECFP4 fingerprints. This information was already present in the legend to Fig. S5 to which this section refers to, but has been added in the main text page 11 of the revised manuscript.

5) The authors state that "SpaceDock remains a relatively light computational procedure since browsing a chemical space of 100 million compounds can be achieved within 2 days on a 16-core Intel(R) Xeon(R) Silver 4210 processor". And just after that "Mining the entire 5.5 billion chemical space has been made.". What is the entire chemical space that they are referring to there?
The full chemical space encoded by the 36 reactions we rely on (page 7, Table S2, Figure S2) covers the 5.5 billion compounds.

6) Revise "Poisening functions" in table S2.
Done (to disallowed functions)

We deeply thank this referee for the helpful criticisms that will contribute to enhance the clarity of the revised manuscript.
